# Molecular Polaritons for Chemistry, Photonics and Quantum Technologies

**DOI:** 10.1021/acs.chemrev.3c00662

**Published:** 2024-02-28

**Authors:** Bo Xiang, Wei Xiong

**Affiliations:** † Department of Chemistry, School of Science and Research Center for Industries of the Future, Westlake University, Hangzhou, Zhejiang 310030, China; ‡ Department of Chemistry and Biochemistry, University of California, San Diego, California 92126, United States; § Materials Science and Engineering Program, University of California, San Diego, California 92126, United States; ∥ Department of Electrical and Computer Engineering, University of California, San Diego, California 92126, United States

## Abstract

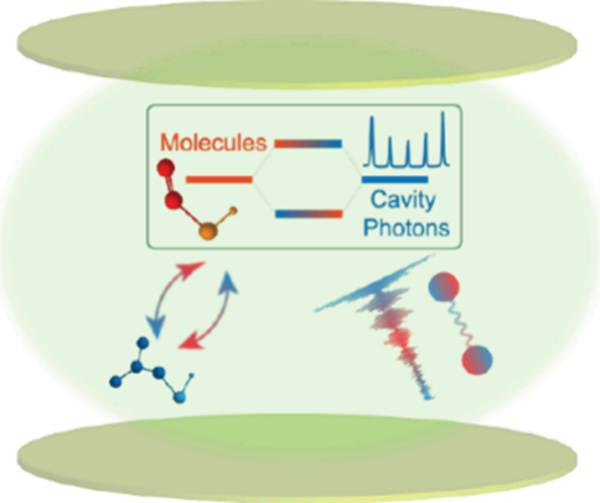

Molecular polaritons are quasiparticles resulting from the hybridization between molecular and photonic modes. These composite entities, bearing characteristics inherited from both constituents, exhibit modified energy levels and wave functions, thereby capturing the attention of chemists in the past decade. The potential to modify chemical reactions has spurred many investigations, alongside efforts to enhance and manipulate optical responses for photonic and quantum applications. This Review centers on the experimental advances in this burgeoning field. Commencing with an introduction of the fundamentals, including theoretical foundations and various cavity architectures, we discuss outcomes of polariton-modified chemical reactions. Furthermore, we navigate through the ongoing debates and uncertainties surrounding the underpinning mechanism of this innovative method of controlling chemistry. Emphasis is placed on gaining a comprehensive understanding of the energy dynamics of molecular polaritons, in particular, vibrational molecular polaritons—a pivotal facet in steering chemical reactions. Additionally, we discuss the unique capability of coherent two-dimensional spectroscopy to dissect polariton and dark mode dynamics, offering insights into the critical components within the cavity that alter chemical reactions. We further expand to the potential utility of molecular polaritons in quantum applications as well as precise manipulation of molecular and photonic polarizations, notably in the context of chiral phenomena. This discussion aspires to ignite deeper curiosity and engagement in revealing the physics underpinning polariton-modified molecular properties, and a broad fascination with harnessing photonic environments to control chemistry.

## 1 Introduction

Polaritons, captivating hybrid quasiparticles, emerge from the strong coupling between matter excitations and virtual photons.^1−3^ Analogous to the formation of a molecular orbital through the hybridization of atomic orbitals, the amalgamation of matter and photon modes generate novel polariton states. These states possess distinct energy and wave functions, characterized by superpositions of both the matter and photonic wave functions. In the time domain, polaritons emerge as new eigenstates when the matter and photon modes exchange energy much faster than each mode dissipates their energy out. Consequently, polaritons inherit properties from both matter and light. For instance, they adopt the nonlinear response of the matter and exhibit large optical nonlinearity in comparison to pure photons,^4−9^ and polaritons inherit the fast velocity of photons and can propagate in media quicker than pure matter excitations.^10−14^

Curiously, the genesis of polaritons does not need external photon injection, as the hybridized photonic modes encompass the virtual photonic state arising from quantum electrodynamics, akin to the zero-point energy inherent in molecular states.^2,15−18^ The light–matter coupling strength of an individual molecule *g*_0_ is determined by , where μ is the transition dipole moment, *E* is the field strength of the electromagnetic field and *n*_*ph*_ is the number of photons. Thus, according to this formula, if the vacuum field strength *E* is very large, even when *n*_*ph*_ = 0; namely, in the absence of external photons, strong coupling can still occur between the molecular modes and vacuum fluctuations. In other words, polariton states inherently exist regardless of photoexcitation. This seemingly unconventional notion stands as a well-established concept in physics, spanning condensed matter physics,^19−32^ and atomic-molecular optics^33−36^ (Rydberg polaritons, single atom polaritons, etc.). This concept holds substantial scientific and technical significance, exemplified by achievements like the realization of room temperature polariton condensates,^37−40^ leveraging polariton propagation to energy or information transfer,^10−12,14^ pushing the boundaries of signal detection thresholds,^8,9^ and engineering single-photon optical transistors through profound polariton nonlinearity.^5−7^

Over the past decade, the polariton concept has significantly expanded its reach into the realms of molecular science and chemistry. Its influence has been profound, demonstrated through achievements like the realization of polariton lasing and condensates employing organic molecules.^3,37−40^ However, the most transformative notion, known as “polariton chemistry” and pioneered by Ebbesen and colleagues,^16−18,41,42^ has emerged as a potential game-changer. This idea, succinctly put, posits that polaritons, characterized by their distinct energy and dual light–matter properties, have the potential to introduce new energy pathways into reactions, thereby exerting a notable influence over reaction rates and selectivity. Initially showcased within the electronic strong-coupling regime on a photochemical reaction,^42^ this concept has now been reported on various photophysical phenomena, including spin dynamics, electron and exciton transports.^43−51^ A more intriguing case, however, is that vibrational strong coupling, namely, strongly coupling molecular vibrational modes with photonic modes, can modify reactions under thermally activated conditions,^41^ without any external photon input. Notably, this idea has found applications in diverse domains related to chemical reactions, energy transportation, phase transition and crystallizations.^52−54^

At the same time, this groundbreaking concept of polariton chemistry has ignited extensive debates concerning its theoretical underpinnings.^55−58^ The crux of the challenge lies in the small light–matter dipole interaction intrinsic to a single molecule, necessitating that 10^6^ to 10^10^ molecules couple to a single photonic mode to engender two polariton states. Consequently, as the byproduct of strong coupling, ∼10^6^ to 10^10^ dark modes coexist with the polaritons, and they are localized, molecular-like states. The presence of these abundant dark modes has prompted theoretical investigations into how the limited population of polaritons can exert pronounced modification to chemical reactions. A comprehension of the role played by dark modes in polariton systems stands as a pivotal pursuit for both electronic and vibrational strong coupling modified phenomena.

For a comprehensive grasp of the interplay between polariton and dark modes, several researchers (including the authors) have harnessed the power of ultrafast spectroscopy and advanced imaging techniques.^12,14,59−61^ These approaches have proven instrumental in unraveling the intricacies of coherence and energy exchanges between the polariton and dark modes. Notably, two-dimensional spectroscopy^59,62−66^ has emerged as a pivotal experimental tool that can uniquely untangle the complex dynamics of polaritons and dark modes that would otherwise remain elusive.

In parallel with the substantial endeavors dedicated to unraveling the mechanisms of polariton chemistry, there exists a concurrent drive to harness the potential of molecular polaritons as a platform for innovative quantum information technology.^4,67−71^ Initial strides have been made in demonstrating delocalized nonlinearity, the initiation of coherences and the strategic design of diverse cavity modalities, all of which contribute to the burgeoning field of applications encompassing quantum information technology and photonic engineering.

While this emerging field of polariton chemistry is in the midst of its evolution across experimental and theoretical fronts, its trajectory underscores the potential for molecular polariton to harness photonic elements in shaping chemical processes and capitalize on molecular characters to modulate photonic responses. Within this context, we will delve into several promising avenues that entail controlling polariton polarizations.

This Review is structured as follows: section 2 provides an introduction to the fundamental theory underpinning polaritons; section 3 explores a variety of cavity types with potential applications in molecular polaritons; section 4 encapsulates the ongoing endeavors aimed at leveraging polaritons for manipulation of reactions and other macroscopic properties; section 5 delves into the pursuit of revealing the intricate interplay between dark modes and polariton states using ultrafast multidimensional spectroscopy; section 6 outlines the latest advancements in harnessing molecular polaritons to establish photonic and quantum technology platforms; last, section 7 elucidates the prospective pathways toward controlling polariton polarization. We would like to point out the wealth of insightful prior reviews^1,3,17,58,67,71−76^ and the current thematic issue.^77−82^ Furthermore, our focus remains intentionally concentrated on experimental works and pertinent theoretical studies. For a more comprehensive overview of theoretical studies of polariton chemistry, we refer the audience to the following reviews from the groups of Yuen-Zhou,^83^ Nitzan,^57^ Rubio,^82,84^ Feist,^85^ Huo,^81^ García-Vidal,^86^ Yelin,^87^ Owrutsky,^75^ and Herrera and Spano.^88^

## 2 Basic Theories of Molecular Polaritons

### 2.1 Hamiltonian of Molecular Strong Coupling

Currently, the predominant focus of the molecular polariton research has been on using the Fabry–Pérot cavity (F–P cavity), constructed by pairing two partial reflective mirrors separated by a distance. In an F–P microcavity, the cavity photon energy can be expressed through the following dispersion form^3,67,70^
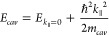
1where *k*_||_ is the wave-vector in the plane parallel to the reflective optics and  is the effective mass related to the refractive index of the media inside the F–P cavity (*n*_*C*_).

The simplest Hamiltonian that encapsulates the light–matter strong coupling is the Jaynes–Cummings model.^89^ This framework was originally developed to describe the coupling between a photonic mode and a single-atomic/molecular mode, which is a rare case in most molecular systems. The Hamiltonian is described by a 2 × 2 matrix, with the two diagonal elements representing a single photon state and a single molecular mode, respectively, and the off-diagonal term representing the single-molecule coupling strength through dipole interactions, denoted as *g*_0_.

**Figure 1:**
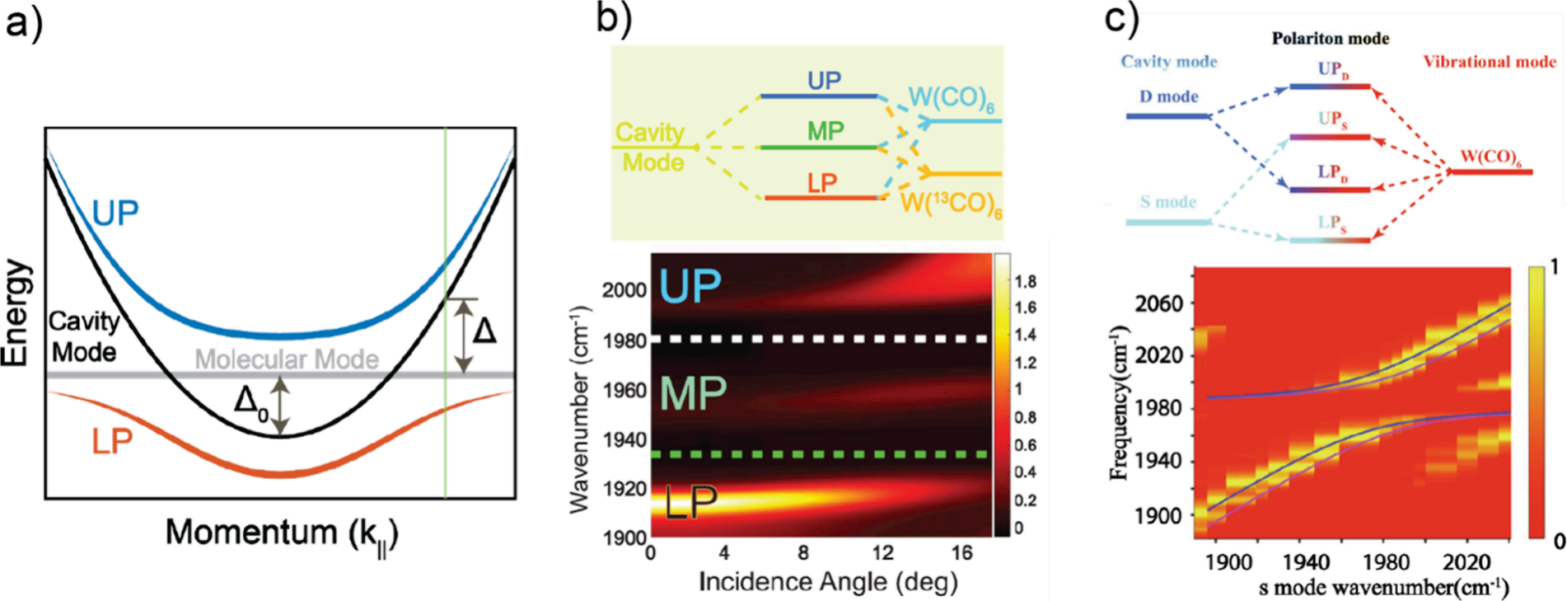
Dispersion curve schematics of various polaritonic systems. (a) Energy–momentum plots of cavity mode (black), molecular mode (gray), UP (blue) and LP (red). The polariton modes are generated by strong coupling between one cavity and one molecular mode, where Δ is the cavity detuning to the molecular mode at a specific *k*_||_, and Δ_0_ indicates the zero-momentum detuning. (b) Polariton dispersion and energy diagram of the strong-coupling system composed of one cavity and the asymmetric C=O stretching modes of two different molecules: W(CO)_6_ and W(^13^CO)_6_.Reprinted with permission from ref (103). Copyright 2020 American Association for the Advancement of Science. (c) Polariton dispersion of the strong-coupling system composed of two cavities and one asymmetric C=O stretching in W(CO)_6_ molecules, where the S and D modes refer to the two orthogonal cavity modes in a confined F–P cavity system. Note that the dispersion shown in (c) was obtained by varying the cavity thickness instead of beam incidence angle, due to the technical complexity of conducting the latter in this confined cavity. Reprinted with permission from ref (70). Copyright 2023 National Academy of Sciences.

After diagonalizing the 2 × 2 Hamiltonian matrix, the two new eigenstates of molecular polaritons can be derived
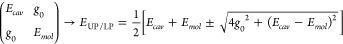
2Because the upper polariton (UP) and lower polariton (LP) are the hybridization of the dispersive cavity photon mode and nondispersive molecular mode, these two eigenstates adopt a dispersive relation. The energies of polaritons undergo modification with a characteristic avoided crossing in the dispersion curve (see Figure 1a). The vacuum Rabi splitting Ω is defined as , which depends on the coupling strength *g*_0_ and cavity detuning (Δ, the energy difference between molecular mode and cavity mode at a specific *k*_||_). Specifically, the detuning at zero wave-vector depends on the cavity longitudinal length, which in the case of the F–P cavity is  for the *m*^th^ order cavity mode, where *n*_*C*_ is the refractive index of the media within the F–P cavity and Δ_0_ is in the unit of cm^–1^.

While the Jaynes–Cummings model accurately describes single-molecule strong coupling, it can also capture many essential features of the so-called collective strong coupling, where many molecules strongly couple to the cavity mode simultaneously. To describe collective strong coupling, the collective coupling strength *g* is used as the off-diagonal matrix element, rather than that of a single molecule. The collective coupling strength arises from the macroscopic polarization of the molecular ensemble and can be described as , assuming that all molecules couple to the cavity at the same single-molecule coupling strength *g*_0_, where *N* represents the total number of molecules engaging in strong coupling with the photonic modes. The Jaynes–Cummings model effectively captures the energetics and wave function compositions of polariton states under the strong-coupling limit.

While convenient for describing the energetics of collective strong coupling, the Jaynes–Cummings model has a severe limitation as it represents the entire matter excitation with a single diagonal element in its Hamiltonian. Consequently, it oversimplifies the physics of the collective strong-coupling regime in all reported polariton chemistry research, such as missing the existence of dark modes.

A more accurate description emerges through the Tavis–Cummings model,^90^ which accommodates the coupling of *N* molecular oscillators to a single cavity mode, described as follows
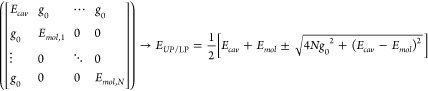
3Employing the Tavis–Cummings model yields several crucial implications: first, in addition to the two optically bright polariton states, there are *N* – 1 optically dark modes that are localized or semilocalized.^57,91−94^ Given that *N*, representing the number of molecules that are strongly coupled with the cavity, typically ranges from 10^6^–10^12^ in a standard F–P cavity,^58,95,96^ the density of state (DOS) of dark modes greatly surpasses that of the polariton modes. Second, the prevalence of inhomogeneity in chemical systems breaks the symmetry of the dark mode wave functions, rendering them “gray”—gaining a small optical brightness.^59,97−99^ Notably, in principle, *g*_0_ and *g* can be increased by the number of photons, *n*_*ph*_. In practice, even in the ultrafast experiments where an intense laser is used to interact with the systems, the Ω (and thereby *g*_0_) was not increased, suggesting that the field strength inside of the cavity is significantly larger than the field strength of the external photons.

With the limitation of the Jaynes–Cummings model in mind, it can still be useful to describe light–matter interactions in more intricate coupling scenarios. Considering the case where two or more nondegenerate molecular modes couple to a single cavity mode,^100−104^ the cavity photon becomes a shared component, modifying the energetics and dynamics of the molecular modes (Figure 1b). The Hamiltonian matrix of one-cavity and two-molecule coupling can be given as
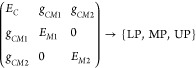
4The polaritonic states can then influence the ultrafast dynamics of intra- and intermolecular energy transfer, and other nonlinear interactions, as discussed in section 5.1.3.

Furthermore, another scenario arises where a single molecular mode couples to multiple-cavity photon modes. In previous studies,^61,69,70^ two cavity modes can strongly couple to the same molecular mode residing within their respective cavity spaces, forming four distinct polaritonic states (e.g., Figure 1c). Within this coupling scheme, the two cavity modes are either weakly coupled (i.e., with a small coupling strength, δ_*C*_) to each other or completely decoupled. A representative Hamiltonian matrix is shown as
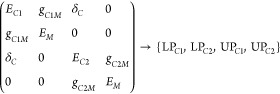
5

A noteworthy difference in this scenario from the one-cavity and two-molecule coupling case lies in the fact that while only a single class of molecular modes are strongly coupled, the localized nature of these modes necessitates their representation through two distinct diagonal matrix elements, each embodying the macroscopic polarization in its own cavity. Notably, even in this framework, molecules remain weakly coupled with the adjacent cavity, effectively serving as a bridge that interlinks the originally noninteracting cavity modes because of energy or spatial separation, thereby manipulating the photonic interactions among cavity modes. In either the one-cavity and two-molecule coupling, or the two-cavity and one-molecule coupling cases, quantum entanglements may be prepared between molecules or polaritons, which is noteworthy for future investigations.

### 2.2 Hopfield Coefficients

The hybridized essence of polaritons bestows significance upon both cavity photon and molecular constituents in shaping the attributes of molecular polaritons, such as energy, momentum, effective mass, and dephasing, among others. The weight of the photonic and matter components, denoted as Hopfield coefficients, can be extracted from the eigenvectors of the Jaynes–Cummings Hamiltonian (eq 2), wherein their squared amplitudes are the Hopfield coefficients, shown as below
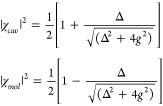
6where the compositions, or Hopfield coefficients, can be adjusted through the cavity detuning, Δ, as well as coupling strength, *g*.

#### 2.2.1 Effective Mass

Given the vast disparity in effective mass between the cavity photon and the molecular mode, the effective mass of hybridized polaritons is comparable to the photon effective mass, as expressed by the following equations^3^
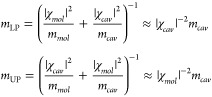
7The substantial decrease in effective mass of molecular polaritons not only heightens the mobility of the quasiparticles, facilitating ballistic and fast diffusive transports, but also implies an extended coherence length determined by the de Broglie wavelength. Consequently, this property facilitates polariton condensates at room temperature.^37,38,105^

#### 2.2.2 Dephasing Lifetime

Another pivotal facet of a molecular polariton, namely, dephasing, is also intrinsically dependent on the photon and molecular constituents. Under the homogeneous limit, the dephasing lifetime of polaritonic modes is inversely proportional to the corresponding spectral line widths. The spectral line width depends the Hopfield coefficients^3^ in the following way:
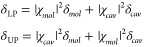
8

Upon strong coupling, the dephasing dynamics can be engineered in several ways, encompassing the fine-tuning of the Hopfield coefficients and the judicious selection of molecular or cavity systems with the desired dephasing characteristics. Such hybridized and tunable characters render molecular polaritons a highly promising template for exploring and simulating various quantum phenomena in molecular systems.

### 2.3 Light–Matter Coupling Regimes

**Figure 2:**
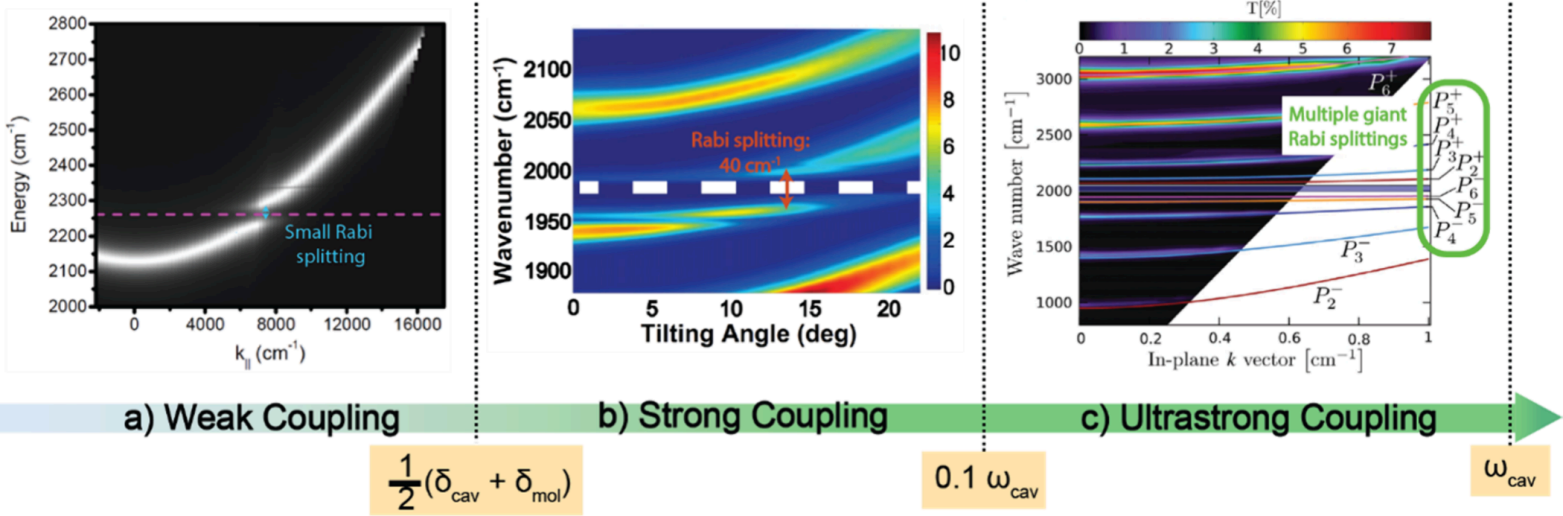
Ranges of coupling strengths and corresponding dispersion curves. (a) Weak-coupling regime. Reprinted with permission from ref (106). Copyright 2015 American Chemical Society. (b) Strong-coupling regime. Reprinted with permission from ref (59). Copyright 2018 National Academy of Sciences and (c) ultrastrong-coupling regime that enables a molecular mode to simultaneously couple to many cavity modes. Reprinted with permission from ref (107). Copyright 2016 American Physical Society.

The coupling strength between cavity photon and molecular modes dictates the regimes of light–matter strong coupling. In the collective coupling regime, the coupling strength scales as
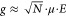
9where μ is the transition dipole moment of the molecular mode, *N* is the number of molecular oscillators in cavity mode volume, and  is the vacuum electric field strength of the cavity mode, where ω_*Cav*_ is the cavity photon frequency, ε_0_ is the vacuum dielectric constant and *V* is the cavity mode volume. Therefore, the coupling strength can be controlled in several avenues: (i) selecting molecular systems with various dipole strengths, (ii) manipulating the molecular density in the cavity volume, and (iii) designing the cavity materials and geometric parameters to control the electromagnetic field strengths. Depending on the coupling strength, the light–matter coupling systems can categorized spanning from weak-coupling to ultrastrong-coupling regimes, as outlined in Figure 2.

#### 2.3.1 Weak-Coupling Regime

The weak coupling between molecular and cavity modes emerges when the coupling strength falls below .^67,108^ Within this context, the exchange between photons and molecules transpires at a rate slower than the average decay rate of cavity photon and molecular modes. Consequently, the essential attributes of photon and molecular modes are preserved. In this regime, the energy-momentum dispersion curves of the hybridized system reveal that the original parabolic shape of the cavity photon mode remains intact^106^ (depicted as the white curve in Figure 2a), albeit with a slight “truncation” in proximity to the nondispersive molecular mode energy (illustrated as the dashed red line in Figure 2a), a distinct departure from the strong-coupling regime (Figure 2b). While the energetics of molecular mode experience minimal alternation, the photoinduced molecular dynamics could be affected.^109−111^ In the context of cavities, when the photon energy is close to the resonant emission frequency of the molecular mode, the Purcell effect^112^ comes into play, altering photobleach^111^ or fluorescence lifetime^109,110^ in the light-harvesting systems.

#### 2.3.2 Strong-Coupling Regime

When the coupling strength is larger than ,^67^ yet remains smaller than 0.1ω_*cav*_, the molecular system enters the realm of strong coupling. Within this domain, the hybridization of these two modes achieves a threshold to form new polaritonic states. For example, Rabi splitting can reach to 40 cm^–1^ in a strongly coupled 40 mM W(CO)_6_/hexane solution at zero cavity detuning, larger than the average line width of molecular and cavity modes, (10 cm^–1^), which results in the characteristic anticrossing features (Figure 2b). Within the polariton lifetime, the Rabi oscillation occurs^4,68,69^ (more details discussed in section 6), showing the rapid energy exchange dynamics between the molecular and cavity modes, which outpace their dissipation dynamics. Studies have highlighted the influence of strong coupling on the relaxation dynamics of the molecular modes^60,103,113^ (see section 5 for more details). It is noteworthy that although most strong couplings were realized in condensed states, a recent work done by Wright, Nelson and Weichman^114^ has realized strong coupling between rovibrational modes and cavities in the gas phase, opening the path to VSC-mediated chemistry or other thermally activated processes involving the gas-phase component. Subsequent sections (sections 4–7) will delve into a comprehensive discussion of the recent advancements in the realm of molecular strong coupling.

#### 2.3.3 Ultrastrong-Coupling Regime

Within the ultrastrong-coupling (USC) regime, the coupling strength is elevated even higher, approaching the range of g≥0.1ω_*cav*_ .^108,115^ Presently, only a few vibrational modes of molecular systems have been claimed to attain the USC limit, such as the O–H mode of water^116^ and the C–O stretch of Fe(CO)_5_^107^ . Alternatively, electronic transitions of the merocyanine (MC)-spiropyran (SPI) systems have been reported to reach the electronic USC limit.^117^ These studies used linear spectroscopy to exhibit extremely large Rabi splitting or achieve multiple Rabi splittings simultaneously among multiple molecular modes and the cavity modes, due to near-degenerate molecular states (Figure 2c). When reaching the USC regime, their energy splitting is significantly enhanced, which can separate the dark modes and polariton states through distinct spectral responses. This advantage sets a contrast to the strong-coupling regime, where the energy difference between polaritonic transitions may be overwhelmed by the line widths if the polariton spectra are broad, resulting in spectral features mixing between polaritons and dark modes. This is particularly important in the multiple-state regime.^118^

Besides, when a single molecular mode couples to multiple orders of adjacent cavity modes in the USC regime^107^ (Figure 2c), the multiple pairs of polaritons can be generated, providing additional degrees of tunability, e.g., creating more polaritonic coherences, enriching the potential pathways of polariton-to-reservoir relaxation, etc. Lastly, the large coupling strength inherent in the USC led to modifications to the ground states, setting it apart from the strong-coupling regime. It thus has been proposed to have the potential to influence chemical reactions happening at ground states.^16,17,76^ Nevertheless, despite its intriguing promise, endeavors to construct or investigate within this realm remain constrained due to the strenuous conditions, such as high molecular concentration^107^ or particular molecular conformation^119^ required to achieve USC, as well as the unresolved intricacies tied to its energetic complexity.

## 3 Photonic Structures for Molecular Strong Coupling

Although the predominant focus of polariton chemistry has been centered on using the F–P cavity, as elucidated in the theory section, alternative photonics structures like nanophotonic cavities hold the potential to augment light–matter coupling, therefore offering new prospects in polariton chemistry. In this section, we provide an overview of diverse cavity designs, evaluating their strength, limitations and associated challenges in relation to their applicability within polariton chemistry research.

### 3.1 Fabry–Pérot Cavity

**Figure 3:**
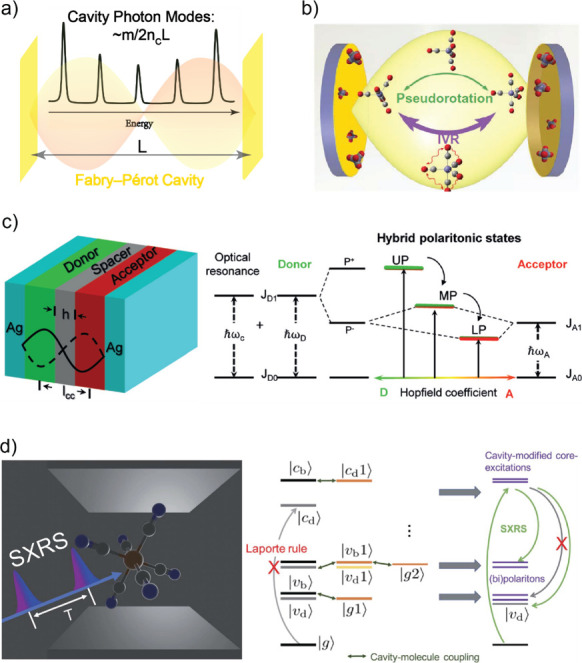
The F–P cavity and schemes of various frequency regimes. (a) Schematic illustration of standing-wave like photon modes (2nd order, as an example here) in a Fabry–Pérot cavity, where the transmission peaks of various F–P cavity modes is shown as the black spectrum. (b) Molecular vibrational dynamics, such as Berry pseudorotation and intramolecular vibrational redistribution (IVR), can be modified by VSC, in the IR-regime cavity. Reprinted with permission from ref (104). Copyright 2022 American Association for the Advancement of Science. (c) Molecular electronic energy transfer mediated by ESC in the visible-regime. Reproduced with permission from ref (44). Copyright 2017 Wiley. (d) Molecular valence and core electronic excitation strongly coupled to cavity mode in the UV/X-ray regime. Reproduced with permission from ref (124). Copyright 2021 Royal Society of Chemistry.

The F–P cavity is the most prevalent type of cavity employed in molecular strong coupling.^4,44,113,120−123,59−61,68,69,98,103,107^ The F–P cavity is composed of two parallel, highly reflective cavity mirrors oriented to face one another (Figure 3a). This design induces standing waves that either transmit or reflect the interfered light, forming an array of cavity modes whose wavelengths are periodically spaced. The cavity (*m*^th^ order) resonance wavelength can be determined based on  where *n*_*C*_ is the refractive index of the media inside the cavity and *L* is the cavity length at normal incidence. To achieve the high reflectivity, either metallic or distributed Bragg reflector (DBR) coating is employed. The metallic coating can achieve a universal broadband reflectivity from ultraviolet/visible to mid-infrared regimes. However, the metallic coating often suffers high absorptivity, additional loss contributing to low quality factor (Q-factor), narrow-ranged penetration depth and heating effects in ultrafast experiments. In contrast, the DBRs provide an alternative solution of highly reflective mirrors. DBRs are based on alternating dielectric-layer coatings of high and low dielectric constants. The reflectivity and penetration depth of the DBR mirrors can be precisely controlled by choosing materials with specific dielectric constants, the thickness of each layer and the number of pairs. With such fabrication approaches, a frequency “stopband” with high reflectivity can be achieved in DBR mirrors.^3^ The combination of two DBR mirrors, separated by a thin gap, forms an F–P cavity with a resonance in the stopband. Alternatively, this cavity gap can be viewed as a void in the periodic dielectric structures, creating a defect—the cavity resonance—in the frequency domain. By capitalizing on the various selections while engineering the F–P cavities, a wide range of applications is unlocked within the realm of strong coupling to molecular excitations.

The F–P cavity can be described by a semiclassical transfer matrix method as follows,^1,60,61,75,106^
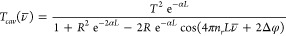
10This equation describes that the transmission of the cavity depends on the frequency-dependent absorption coefficient (α) and refractive index (*n*_*r*_) of the material within the cavity, among other parameters. *T*, *R*, *L*, and Δϕ are the transmission, reflectivity, thickness, and phase shift of the cavity, respectively. The frequency-dependent refractive index, *n*_*r*_, and absorption coefficient, α, can be formulated as
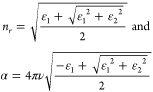
11ε_1_ and ε_2_ are the real and imaginary components of the dielectric function, respectively, which are defined as a sum of multiple Lorentzian oscillators as below
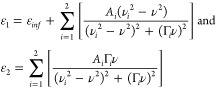
12where *A*_*i*_ is the amplitude, Γ_*i*_ is the full line width associated with the *i*^th^ oscillator inside the cavity, *ε*_*inf*_ is the background dielectric constant at the infinite frequency, υ_*i*_ are the frequencies of the 0 → 1 (*i* = 1) or 1 → 2 (*i* = 2) asymmetric stretch transitions, and υ is the variable of frequencies. The amplitudes of Lorentzian oscillators are directly related to their concentrations. Upon optical excitation, the oscillators are pumped to higher-order excited states, leaving a reduced ground-state concentration, i.e., *A*_0_. The change of amplitude terms could further lead to the differential transmission, i.e., pump–probe signal of the strongly coupled molecular systems.

In the realm of the infrared regime, the F–P cavity mode can be adjusted to align energetically with vibrational modes, thereby engendering molecular vibrational polaritons (MVPs). This interaction brings about a hybridization between cavity photon and molecular vibrational modes. As a result, the hybridization leads to changes in vibrational population relaxation,^60,68,113^ ultrafast molecular isomerization processes (Figure 3b)^104^ and vibrational coherent^68−70^ and incoherent^61,103^ energy transfer. Vibrational strong coupling (VSC) can also induce alterations in chemical reactivity, be it through reduction or enhancement.^41,42,120,125−127^ However, despite its potential, the underlying mechanism remains to be clarified and issues of reproducibility exist within certain reported instances.^56,126,128−130^

In the visible regime, a multitude of molecular electronic excitations has been demonstrated to be amenable to strong coupling (Figure 3c). This capability extends to the control of the Förster resonance energy transfer between donor and acceptor molecules^131,132^ and encompasses photochemical processes, such as isomerization.^42^ Furthermore, the coupling of molecules with chiral cavities offers avenues for the manipulation of molecular chiral responses, such as enhancing the circular dichroism effect.^133^

When the cavity mode wavelength further decreases into the domain of regular or deep UV ranges, the molecular valence-band electronic transitions can be coupled.^124,134^ The UV polariton of the valence band was formed in a strongly coupled prototypical model transition metal complex, ferricyanide ([Fe(III)(CN)_6_]^3–^) aqueous solution, and the core band of the complex at higher frequencies was also modified by the cavity modes. Such a polariton system was studied theoretically using computational stimulated X-ray Raman spectroscopy (SXRS, X-ray pump pulse followed by a delayed X-ray probe pulse, in which a Raman process reveals the transitions between valence and core states, pulse sequence shown in Figure 3d, left panel). Computational SXRS indicates that the UV polariton engenders new pathways for population transfer pathways between the valence and core electronic states. For example, transitions from the cavity-modified core bright mode—the two polaritonic core states near |c_b_>—to the (bi)polaritons near the valence bright mode (|v_b_>) and the cavity-dressed valence dark mode (|v_d_>) become symmetry allowed. Such extra correlation between valence- and core-level states in an optical cavity has been attributed to the energetic shifting alongside the Rabi splitting^124^ (Figure 3d, right panel). In addition, it was demonstrated in the simulation that UV polaritons of a pyrazine molecule can modify electronic dynamics near conical intersections (CI), by creating new polaritonic CI or shifting existing CI in terms of energy and spatial coordinates.^134^ The realm of X-ray regime molecular strong coupling^124,135^ has also garnered theoretical consideration, centered on the proposition of splitting the core electronic state into polaritonic modes without affecting the valence states.

Overall, the remarkable tunability of F–P cavity resonances, achieved through selection of cavity materials and manipulation of geometric parameters, renders it an exceptional photonic platform to modify molecular properties. However, this type of cavity is not exempt from certain limitations, as detailed here. (i) Cavity volume and DOS of dark modes: The requirement that the cavity thickness must be greater than λ/2 results in a relatively large cavity volume. Subsequently, it sets a fundamental limit of the electromagnetic field strength and necessitates a considerable number of molecular oscillators to reach strong coupling, thus resulting in the high DOS of dark modes, as discussed in the Basic Theories of Molecular Polaritons section. (ii) Energetics and dephasing variance: The cavity’s energetics and dephasing are significantly susceptible to environmental factors, such as temperature, cavity length and mirror defects. This sensitivity leads to variations of molecular strong coupling. (iii) Spatial and momentum constraints: Optical investigations within this cavity configuration adhere to classical optics principles, resulting in limitations such as spatial resolution constrained by the diffraction limit and a restricted momentum range dictated by the maximal incidence angle. Addressing these limitations extends beyond mere adjustment in the F–P cavity. Instead, it is necessary to develop and deploy other types of cavity or photonic structures, as elaborated in the following sections.

### 3.2 Plasmonic (Phonon)–Polaritonic and Plasmonic–Photonic Coupled Cavities

Plasmons are the collective oscillations of free electrons within metal or other dielectric materials. These plasmonic modes are commonly induced by the interactions between incidence light fields and plasmonic resonators, whose mode volume is confined to the subwavelength scale. Therefore, the samples that couple to the plasmon mode normally have low dimensionalities.

#### 3.2.1 Surface Plasmon (Phonon) Polariton Resonances

**Figure 4:**
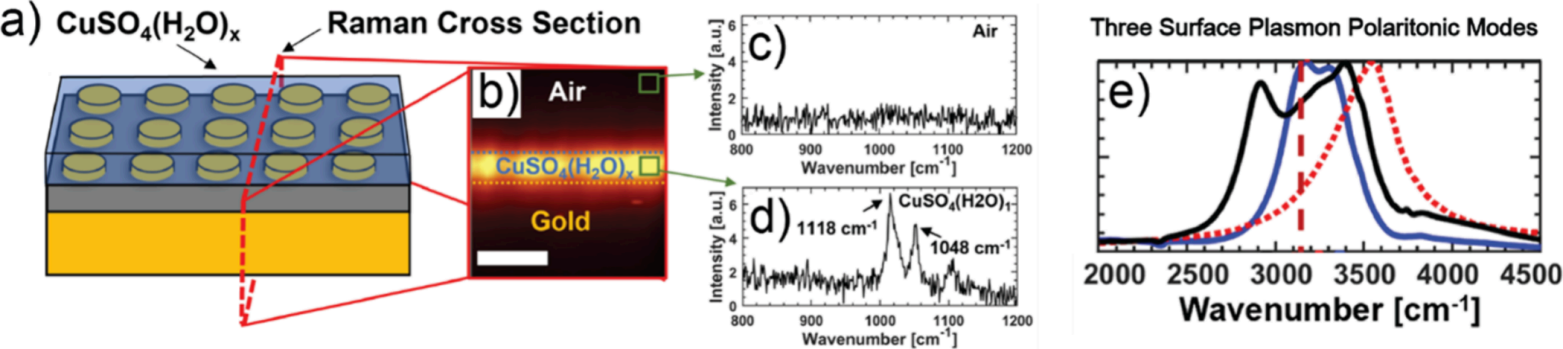
Strong coupling of water OH stretches CuSO_4_(H_2_O)_1_ with surface plasmon polaritons. (a) Schematic of the plasmonic substrate with 2 μm CuSO_4_(H_2_O)_1_ on top. The red dotted lines show the Raman map region. (b) Raman depth map with horizontal dashed lines indicating the boundaries of the different regions. The scale bar is 4 μm. (c) Spectrum obtained by the Raman map above the substrate. (d) Spectrum obtained from the film region showing the prominent peaks of CuSO_4_(H_2_O)_1_ at 1118 and 1048 cm^–1^. (e) Spectral absorbance (normalized) of a bare plasmonic substrate (red dashed), a CuSO_4_(H_2_O)_1_ thin film on Al_2_O_3_ with a gold back reflector (blue), and the spectrum of the CuSO_4_(H_2_O)_1_ thin film on top of the plasmonic substrate composed of a nanodisk (diameter = 680 nm) with a plasmonic resonant frequency of 3534 cm^–1^ (black). The black spectrum shows clear doublet peaks, indicating strong coupling between OH stretches and plasmonic modes. Reproduced with permission from ref (28). Copyright 2021 American Institute of Physics.

One approach involves a surface plasmon polariton (SPP) generated on a metallic or dielectric surface.^28−32^ SPPs exhibit a high degree of localization near the surface, making them amenable to coupling with the samples close to the surface, such as a thin film. Brawley et al.^28^ employed a gold nanodisk structure designed to generate angular dependent surface plasmon mode (Figure 4a). The CuSO_4_(H_2_O)_1_ thin film was deposited onto such a substrate, and the Raman depth cross sections (Figure 4b) showed resonance peaks at 1118 and 1048 cm^–1^, confirming that the copper sulfate monohydrate molecules were distributed near the gold nanodisk (shown in Figure 4d, as compared to the background spectrum of air, far away from the nanodisk region in Figure 4c). In this work, the authors detuned the plasmon modes away from the copper sulfate resonance, via changing the nanodisk diameter. Despite the broad line width of the plasmon mode, both symmetric and asymmetric vibrational modes of water molecules within the copper sulfate monohydrate thin film engaged in strong coupling, each with slightly different but sufficiently large coupling strengths, giving rise to a three-polaritonic system (Figure 4e).

**Figure 5:**
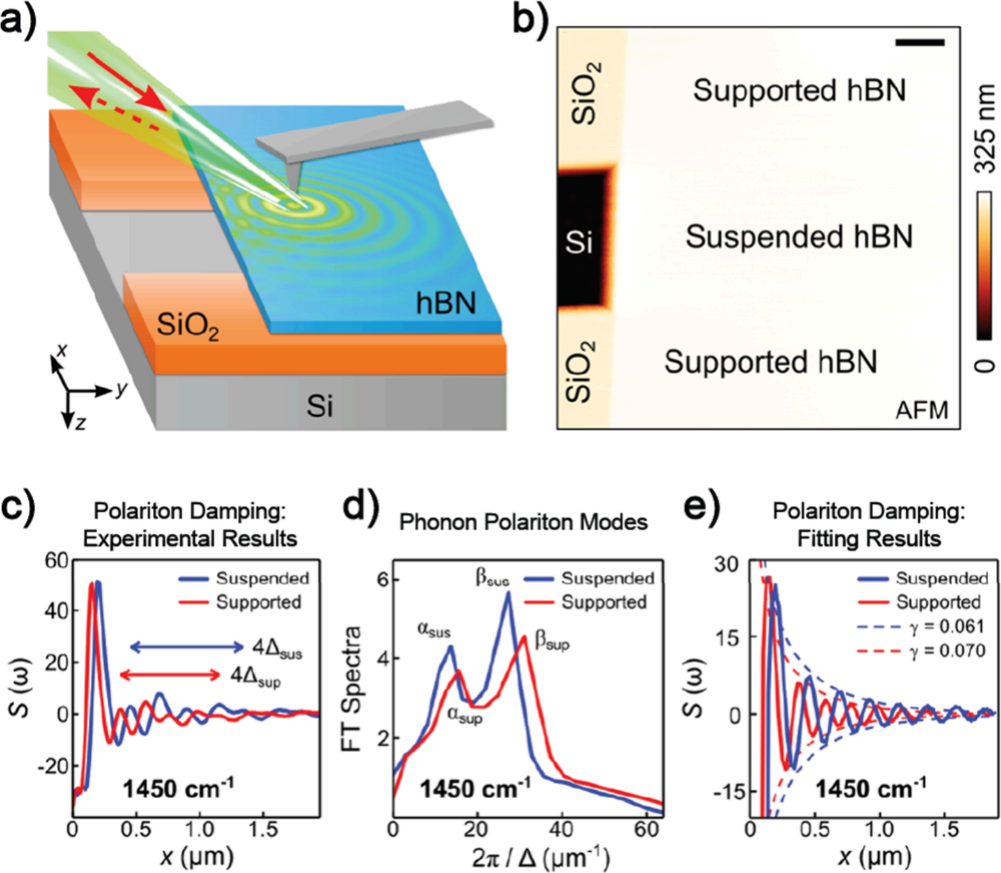
Scattering-type scanning near-field optical microscopy (s-SNOM) images of hyperbolic phonon polaritons in hexagonal boron nitride. (a) Experiment setup. An exfoliated microcrystal of hBN (thin film, tens of layers) was transferred onto the Si/SiO_2_ substrate with an air trench such that part of the hBN was suspended. In the experiment, the AFM tip is illuminated (red solid arrow) by an infrared (IR) beam from a quantum cascade laser (QCL). The backscattered IR signal was then collected (red dashed arrow). (b) AFM image of the suspended and supported hBN studied in this experiment. (c, d) s-SNOM line profiles for hyperbolic phonon polaritons in suspended (blue) and supported (red) hBN. (c) s-SNOM line profiles taken along horizontal axes within supported and suspended hBN regions, the fringe periods are indicated with double arrows (blue and red arrows for Δ_sus_ and Δ_sup_, respectively). (d) Fourier transform spectra of the s-SNOM line profile in (c). α and β indicate phonon polariton peaks in the FT spectra. (e) Line profiles of Δ = λ_p_/2 (λ_p_ is the polariton wavelength and Δ is the typical polariton fringe period close to the hBN edge) and the corresponding damping factor γ. The line profiles were obtained by inverse Fourier transform of the spectra in (c). IR frequency ω = 1450 cm^–1^. Reproduced with permission from ref (23). Copyright 2019 American Chemical Society.

In a quest to exploit the reduced dimension of cavity volumes, Dai et al.^23^ deposited a notably thinner sample—an atomically layered hexagonal boron nitride (hBN)—onto a Si/SiO_2_ open cavity, generating surface phonon polaritons (Figure 5a). The hBN geometry was carefully designed to feature both partial suspension and support regions (Figure 5b), in order to delve into how geometric factors affect the spatial transport of such surface phonon polaritons. The authors obtained the scattering-type scanning near-field optical microscopy (s-SNOM) line profile (Figure 5c) corresponding to the phonon polariton modes of different geometric regions, from which polaritonic peaks can be extracted by Fourier transformation. From Figure 5d, they confirmed both suspended and supported hBN phonon modes strongly couple to the open cavity mode with sufficient Rabi splitting. By fitting the damping rates of these two different polaritonic structures, the dynamics were characterized by a damping factor, defined as the ratio between the imaginary and real components of the complex polariton in-plane momentum, *k* = *k*_1_ + *ik*_2_, where *k*_1_ = 2π/λ_p_ and *ik*_2_ corresponds to the spatial propagation and damping components of the phonon polariton modes, respectively. The suspended mode exhibited a slower polariton damping factor (*k*_2_/*k*_1_), 0.061 versus 0.070, for the supported hBN region, as shown in Figure 5e. This contrast can be understood by the fact that the supported hBN experienced additional environmental loss due to the substrates, compared to the suspended ones. The control over the damping rate could lengthen the polariton coherence propagation length, thereby underscoring the significance of geometric parameters in surface phonon polaritons.

**Figure 6:**
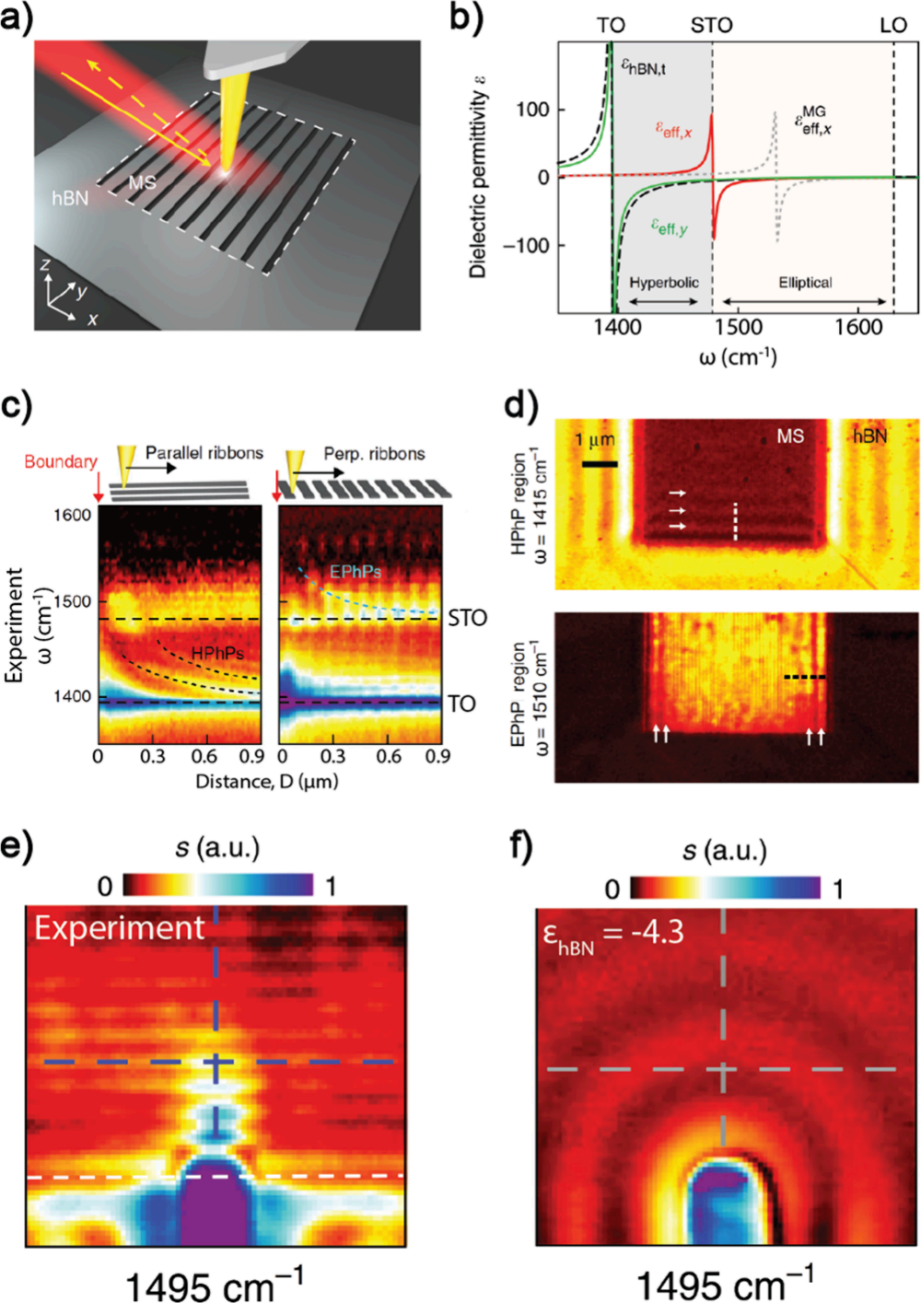
Phonon polariton induced by strong coupling between hBN phonon mode and photonic mode with grating-aggregated hBN nanoribbon structure. (a) Schematic of the near-field nanoimaging experiment. (b) Calculated anisotropic effective dielectric permittivity (real parts) of the metasurface, *ε*_*eff,x*_ (red line) and *ε*_*eff,y*_ (green). Permittivity of unpatterned hBN (*ε*_*hBN*__,*t*_, dashed black line) and effective permittivity of the metasurface based on Maxwell Garnett approximation (ε_*eff,x*_^*MG*^, dashed gray line) are provided for comparison. (c) Near-field spectroscopic line scans taken parallel (left panel) and perpendicular (right panel). The parallel scan shows the hyperbolic phonon polariton whose energy is slightly higher than the TO phonon mode, and the perpendicular scan shows the elliptical phonon polariton with an energy above the STO phonon mode. (d) Near-field images measured at ω = 1415 cm^–1^ (HPhP region, top panel) and ω = 1510 cm^–1^ (EPhP region, bottom panel). White arrows indicate the polariton fringes observed on the metasurface which records the propagation direction. (e) Experimental near-field distribution of antenna-launched elliptical polaritons on the metasurface at ω = 1495 cm^–1^. White dashed lines mark the boundary of the metasurface. The polaritons show collimated propagation, namely, canalization. (f) Experimental near-field image at ω = 1495 cm^–1^ for the case of an antenna located on an unpatterned area of the same hBN flake, which exhibits isotropic propagations. Reproduced with permission from ref (27). Copyright 2020 Nature Publishing Group.

In a particularly elegant design by Li et al.,^27^ they showed the capability of hBN phonons to strongly couple to the photonic modes from a grating structure composed of the same hBN nanoribbons, supporting two types of polariton modes, one of which exhibited a collimated propagation, known as canalization. This grating structure (experimental scheme shown in Figure 6a) allows the strong collective near-field coupling between adjacent individual hBN ribbons, forming a new synthetic transverse optical (STO) phonon mode at 1478 cm^–1^, traveling perpendicular to the ribbons (*x* axis in Figure 6a). This STO mode is about 80 cm^–1^ blue-shifted relative to the natural transverse optical (TO) phonon modes (Figure 6b), traveling along the *y* axis. Notably, in the spectral region between TO and STO modes, the in-plane permittivity had opposite signs, with *ε*_*eff*__,*x*_ > 0 and *ε*_*eff*__,*y*_ < 0. This condition supports the so-called hyperbolic phonon polaritons (HPhPs), a name given by its hyperbolic dispersion relationship. Above the resonant frequency of STO, both permittivities are negative *ε*_*eff*__,*x*_ < 0, *ε*_*eff*__,y_ < 0, and |ε_*eff*,*x*_| > |*ε*_*eff*__,*y*_|. This leads to the elliptical phonon polaritons (EPhPs), with an elliptical dispersion curve, and the modes should be propagating along the *x* axis.

The experimental measurement, using s-SNOM, verified the existence of both polariton modes. By line scanning a metallic tip probe, the spectral signature of TO and STO can be visualized along the parallel or perpendicular direction to the ribbons, respectively (as shown in Figure 6c). Strikingly, when scanning along the parallel direction to the ribbons, only the HPhPs were clearly observed below the STO frequency (Figure 6c, bottom left), while in the perpendicular scanning direction, the EPhPs appeared at a higher frequency region than the STO resonance (Figure 6c, bottom right). This directionality can be clearly visualized by the interference pattern of forward and backward polaritons at the edge of the materials—the HPhPs (Figure 6d, top panel) and EPhPs (Figure 6d, bottom panel) showed the orthogonal fringes near the boundaries, as indicated by the white arrows, providing direct evidence of the orthogonal propagation directions. More interestingly, the real space images of EPhPs clearly showed that the polariton modes propagate in a collimated manner perpendicular to the gratings (Figure 6e), reaching the canalization mode, whereas the control of an unstructured hBN exhibited isotropic propagation (Figure 6f). Such photonic structures provide an innovative avenue for confined photonic modes for strong coupling and manipulate its transport properties.

#### 3.2.2 Plasmonic-Enhanced Cavities through Tip Junctions

**Figure 7:**
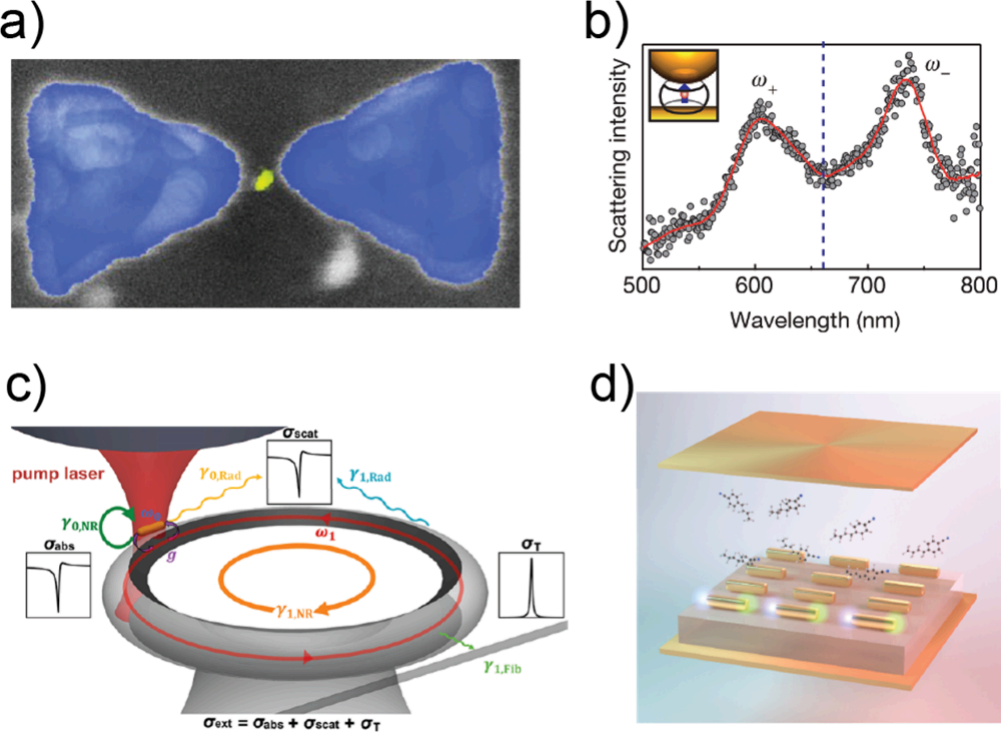
Platforms that can reach single- or few-molecule strong-coupling regime. (a) Plasmonic single-molecule strong-coupling scheme between quantum dots (QDs, e.g., Cd/Se/ZnS) and silver plasmonic bowtie structure. Reproduced with permission from ref (137). Copyright 2022 American Chemical Society. (b) Molecular plasmonic polariton modes, induced by the strong coupling between a single methylene blue dye molecule and gold nanospheres. Reproduced with permission from ref (138). Copyright 2016 Nature Publishing Group. (c) Plasmonic–photonic coupled cavity composed of plasmonic gold nanorods (AuNRs) and a silica toroidal whispering-gallery-mode (WGM) resonator. Reproduced with permission from ref (142). Copyright 2020 American Chemical Society. (d) Molecular (4-butylbenzonitrile and hexanal) strong coupling in gold-coated Fabry–Pérot cavity with plasmonic gold nanorods. Reproduced with permission from ref (143). Copyright 2021 American Chemical Society.

Besides the surface-specific plasmon (phonon) polariton cavities, the bowtie or tip–substrate configurations offer an alternative geometry featuring ultrasmall cavity mode volumes, coupled with plasmon modes. In the surface plasmon (phonon) polariton structures discussed in section 3.2.1, the electromagnetic fields experience a dramatic surface enhancement owing to the mode confinement in direction normal-to-plane (Figure 7a and b), while bowtie or tip–substrate micro- or nanocavities have their mode volumes confined in all three spatial dimensions, leading to an ultrasmall volume. When molecular systems are positioned in close spatial and energetic proximity to such a plasmonic cavity mode, their molecular responses can be enhanced under even weak coupling conditions.^136^ Furthermore, molecular plasmonic polaritons become feasible in the strong-coupling regime.^58,137−141^

The plasmonic cavity mode spans a broad range of frequencies, thereby facilitating coupling with both molecular vibrational^28^ and electronic excitations.^137,138^ Furthermore, the highly localized mode volume enhances electromagnetic field strength, rendering a small number of molecular oscillators adequate for establishing the strong-coupling regime. Notably, the plasmonic cavity has emerged as the sole platform to realize single-molecule polaritons at room temperature.^137,138,140,141^ Using a bowtie (Figure 7a) or nanosphere structure (Figure 7b), the plasmonic cavity can be formed by a close-to-flat metallic surface with its mirror imaged counterpart. In addition, through a tip–substrate junction, a limited number of oscillators such as quantum dots, dye molecules, etc. can strongly couple to the plasmonic cavity mode with such highly localized mode volume. At a single- or few-molecule regime, the molecular orientation in relation to the incident light field takes a crucial role.^138^ The sensitivity of such orientational information not only enhances the tunability of the plasmonic for molecular sensing but also positions it as an ideal platform to delve into the fundamental theories governing single-particle phenomena.

#### 3.2.3 Plasmonic–Photonic Cavity

While plasmonic and photonic cavities distinctly hold their strength within the realms of molecular strong coupling, with the former featuring stronger field strength and the latter possessing high Q and tunability, it is intriguing to build a hybrid cavity combining advantages of both plasmonic and photonic modes,^142,144−150^ offering augmented tunability and signal enhancement. A prevalent approach in creating such plasmonic–photonic coupled cavities involves the hybridization between whispering-gallery mode (WGM, as the photonic component) and metal nanostructure mode (as the plasmonic component).^142,149,150^ As shown in Figure 7c, the localized excitation of metal nanorods can shift the refractive index of the optical microresonator owing to the photothermal effect upon plasmonic excitations. Consequently, the WGM frequency can be flexibly adjusted in response to factors such as the power fluence, plasmonic structure geometries and Q-factors of the WGMs. Another design^143^ aimed at coupling plasmonic and photonic modes entails placing a metal nanostructure on one of the mirrors of an F–P cavity (Figure 7d). This incorporation of a 2D lattice array of plasmonic mode notably elevates the electric field strength near the mirror surfaces, resulting in an order of magnitude increase in its coupling strength with molecular modes situated near the optical surfaces. Conversely, the plasmonic mode experiences spectral line width narrowing, thanks to the plasmonic lattice resonance, culminating in a longer dephasing lifetime of polaritons.

Despite the achievement in plasmonic–photonic hybridization, the strong couplings between molecular modes and either plasmonic or photonic mode remain separated: the former culminates in polaritons arising from highly localized fields and a small number of molecular oscillators, whereas the latter transpires within a delocalized ensemble regime. One potential avenue to propel the advancement of such systems lies in overlapping the plasmonic and photonic mode volumes so that both modes together can reach strong coupling with the molecular modes. Consequently, the polaritonic states possess the nonlinearity from the molecular mode, with a potential of single-molecule regime strong coupling due to the augmented electromagnetic fields of the local plasmon, and at the same time inherit the delocalization from the optical components. This novel realm of molecular strong coupling would lead a fresh paradigm in polariton-mediated molecular dynamics, chemistry and quantum simulation.

### 3.3 Optomechanical Cavity

**Figure 8:**
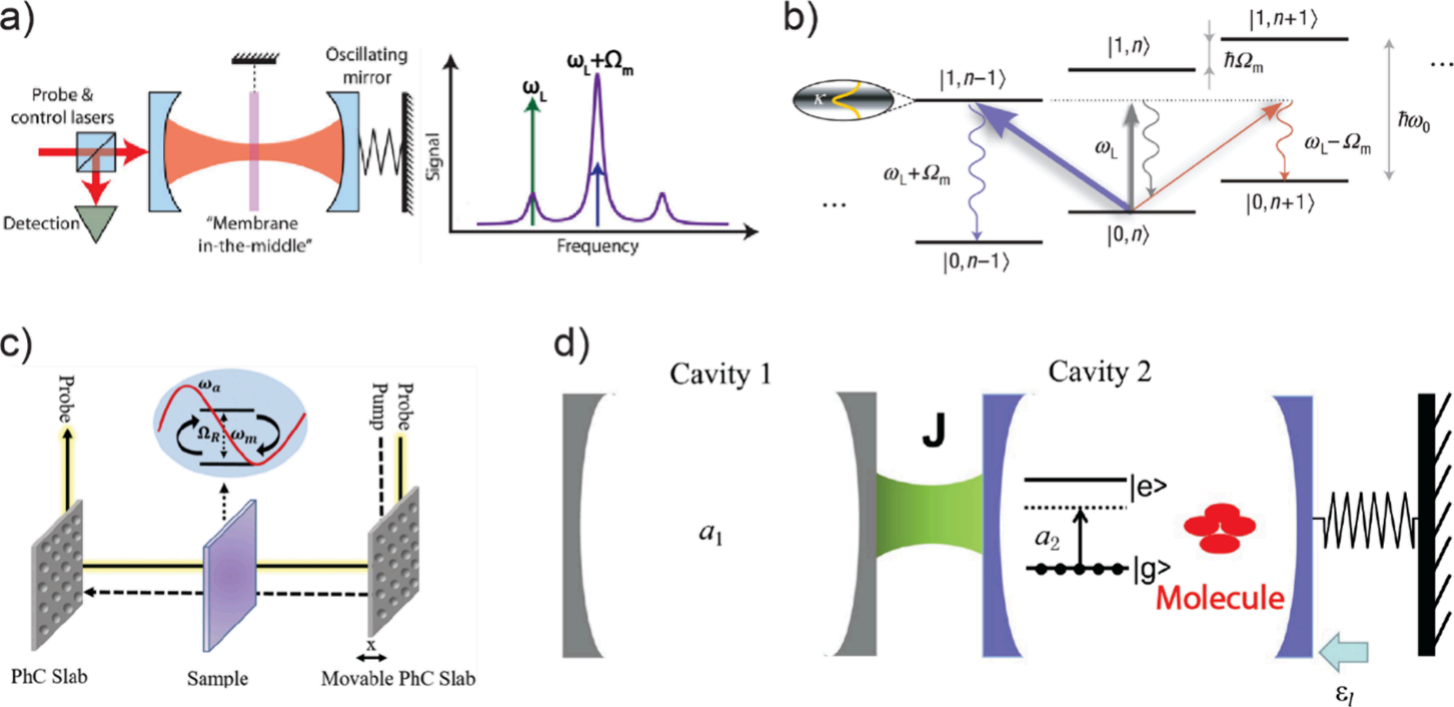
Optomechanical strong-coupling systems. (a) Optomechanical cavity with optical and mechanical strong coupling, where sub-bands in the output signal show the frequency of the mechanical mode. The green arrow indicates that upon pumping the sidebands at the red-detuned frequency, a phonon mode of the mechanical part is added to the photons, leading to a re-emission near the central frequency (natural frequency of the cavity mode, blue arrow). Reproduced with permission from ref (154). Copyright 2014 American Institute of Physics. (b) Energetic picture showing the addition and extraction of a phonon from the mechanical mode upon laser excitation at the red-detuned sideband frequency (*ω*_*L*_). Reproduced with permission from ref (163). Copyright 2008 Nature Publishing Group. (c) Illustration of a double-photonic crystal slab cavity (DPhC) with a mode of frequency ω_a_ and molecular [W(CO)_6_] transition frequency ω_*c*_, coupled to the PhC slab vibrational mode of frequency, ω_*m*_, and the W(CO)_6_-cavity coupling strength Ω_R_. Reproduced with permission from ref (156). Copyright 2022 American Institute of Physics. (d) Proposed scheme of joint optomechanical-optical cavity with molecular strong coupling to enable both optical-optomechanical molecule/atom-optomechanical couplings. Cavity 1 is an optical cavity, and cavity 2 is an optomechanical cavity. J is the coupling strength between the two cavities, *a*_1_ and *a*_2_ correspond to the creation of cavities 1 and 2, and *ε*_*l*_ is the external field applied to cavity 2, e.g., a pump laser. Reproduced with permission from ref (161). Copyright 2018 Nature Publishing Group.

A promising hybrid cavity in light–matter strong coupling is the optomechanical cavity,^151−164^ which involves the interaction between the optical cavity mode and the mechanical mode. The mechanical mode often pertains to the vibrational motion of a membrane within the optical cavity or the mechanical oscillation of a spring mounted on one of the photonic cavity mirrors^151,153,155^ (Figure 8a, left panel). The cavity photons would be modulated by the mechanical oscillation via Stokes and anti-Stokes Raman scattering, which forms two sidebands deviating from the original photon frequency by the amount of the mechanical frequency (Figure 8a, right panel). To reach the optomechanical strong-coupling (SC) regime, the mechanical frequency needs to be larger than the line width of the central cavity photon mode. The formation of such sidebands via SC can trigger the phonon addition (red arrow in Figure 8b) and extraction (blue arrow in Figure 8b) processes from the mechanical oscillators. The latter is more intriguing since the reduction of phonons from the mechanical mode helps “cool” the mechanical ground state with much lower quantum noise, considering the lowering of its thermal fluctuation. To achieve the sideband cooling in the optomechanical cavity, a pump laser with red-detuned frequency (as shown in green arrow in Figure 8a, right panel) should be exerted (*ω*_*L*_), exciting to the energy level of |1,*n*–1> (excited state after one phonon is extracted from the mechanical oscillator), subsequent with a preferential re-emission of cavity photons (*ω*_*L*_ + Ω_*m*_, blue arrow in Figure 8b), causing one quanta of phonon mode, Ω_*m*_, to be extracted from the mechanical mode. The opposite phonon addition can be done when pumping the |1,*n*+1> states. When a “squeezed” pump light, with low energy uncertainty, is used, it enables more precise and efficient additions and extractions of a single phonon from mechanical oscillators to the cavity photon.^151,153,155^ This approach further validates the exceptional sensitivity and tunability of the optomechanical cavities. Thus, it can be rendered to be an invaluable toolkit for sensing and detection of molecules when the molecular motions are coupled with the mechanical or optical modes.

Equally pivotal, theoretical inquiries have proposed the incorporation of mechanical modes alongside molecular light–matter strong coupling. Barbhuiya et al.^156^ proposed a model system featuring W(CO)_6_ within a photonic crystal (PhC) optomechanical cavity (Figure 8c), where the engaged molecular vibrational mode is strongly coupled with the photonic cavity mode, forming polaritons. Meanwhile, mechanical–photonic coupling results in optomechanical induced transparency (OMIT), leading to a narrow frequency window with high transmission, and greatly alters the line shape of the cavity photonic mode. This reshaped cavity modes then strongly couple to the molecular modes, with more intricate polariton energetics. Given that the frequency and intensity of OMIT are contingent on the detuning between cavity resonance and mechanical frequency,^152^ the tripartite coupling of molecular, photonic and mechanical modes assumes significance, in the context of molecule-based quantum information processing, distinguished by heightened sensitivity and low noise.

Moreover, the coupling of optical and optomechanical cavities^160,161^ (Figure 8d) further extends the potential of optomechanics. This innovative theoretical scheme allows three strong-coupling effects: (i) between a molecule/atom and cavity 2, (ii) between mechanical oscillator and cavity 2, and (iii) between cavities 1 and 2. It was proposed that this design can build a new quantum information process platform: when the molecular and mechanical modes both couple to cavity 2, the quantum interference can be then induced between the newly formed states, which can be modulated via the coupling between cavity 1 and 2. For example, the cavity 1 can reshape the light field and “squeeze” it energetically to “cool” the optomechanical state, or cavity 1 can help create more photon reemission channel upon the energetic modulation in coupling to cavity 2. The joint optical–optomechanical cavity design enables high tunability of quantum states within a molecular–optomechanical system, potentially facilitating the development of sophisticated quantum platforms.

### 3.4 Summary

The selection among various types of cavities is ultimately determined by the characteristic of molecular excitations, like energy and momentum of the polariton dispersion curve in the frequency domain or the excited-state dynamics in the time domain. Such properties are strongly related to the frequency-dependent reflectivity, Q-factor and mode lifetime that depend on the cavity material selections and geometric parameters. Furthermore, the dimensionality of molecular systems should be taken into consideration when designing the proper cavity mode volume (either delocalized in three dimensions or confined in one or two dimensions) to realize robust light–matter strong coupling. Additionally, spatial visualization is essential in comprehending the intermolecular interactions, especially in the context of hybrid cavities. Table 1 summarizes and compares attributes of different types of cavities or photonic structures. Notably, the above-mentioned parameters span across multiple dimensions, encompassing energy, time and space. The imperative for multidimensional characterization not only propels the optimization of cavities in terms of geometry and materials, and comprehension of cavity modified reactions as discussed below, but also catalyzes the evolution of the combined spectroscopy and microscopy^61,69,165^ that cooperate synergistically in the study of molecular strong coupling.

**Table 1:** Comparison among Different Cavities or Photonic Structures

Type of Cavity		Geometric Design	Fabrication	Typical Cavity Mode Volume	Typical Cavity Q-Factor	Applications in Molecular Strong Coupling
Fabry–Pérot cavity	Metallic coated	Two parallel cavity mirrors facing each other with proper longitudinal lengths introduced by a spacer or sample layers	Electron-beam evaporation, sputtering, etc.^42,128^	10^4^–10^5^ (μm^3^)^166^	100–2000^42,120^	Strong-coupling modified chemistry, polariton lasing, polariton condensates
	DBR coated		Electron-beam evaporation, sputtering, photolithography, etc.^61,69,167^	10^4^–10^5^ (μm^3^)^4,166^	100 to (>10^4^)^4,59,60,167^	
Plasmonic cavity	Surface plasmon/phonon polariton structure	Two-dimensional photonic/plasmonic structures	Reactive ion etching, electron-beam lithography, epitaxial growth, etc.^23,24,168^	In-plane: spread out and propagating; out-of-plane (normal-to-surface): <200 nm^23,169^	10–100^138^	Modifying surface-specific chemical behaviors, in-plane polaritonic emission and propagation, etc.
	Nanoplasmonic structure	Two nanostructures forming a plasmonic junction	Electron-beam evaporation, chemical vapor deposition, wet chemistry, etc.^170,171^	10^1^–10^8^ (nm^3^)^137,138^		Single-photon emission, cavity-induced coherent interactions between single molecule/emitter, etc.
Plasmonic–photonic cavity		Photonic cavity with plasmonic nanostructure embedded	Electron-beam evaporation, sputtering, thermal growth, etc. for photonic parts; electron-beam lithography, wet chemistry growth, etc. for the plasmonic parts^142,143,172^	Localized plasmonic mode (10^1^–10^8^ nm^3^) in a delocalized photonic environment (10^4^–10^5^ μm^3^)^142,143,166^	(>10^7^)^142,144^	Controllable local energy dissipation in molecular systems, vibropolaritonics, plasmon-based molecular sensing, etc.
Optomechanical cavity		Fabry–Pérot cavity with a mechanical oscillator embedded	Electron-beam evaporation, sputtering, thermal growth, etc. for photonic component; the mechanical parts will be attached to the photonic component^156,163,173^	10^4^–10^5^ (μm^3^)^156,163,166,173^	10^4^ to (>10^7^)^156,163,173^	Not used in molecular strong-coupling experiments but shows potential in developing polaritonic quantum system with lower quantum noise and higher sensitivity

## 4 Molecular Reactions Modified by Strong Coupling

A primary attraction of molecular polaritons lies in the capacity to leverage their hybridized nature and modified energy landscape to influence chemical dynamics and reactions. This concept was initially demonstrated through photoinduced chemical reactions under electronic strong coupling^42^ and subsequently expanded to thermally activated reactions^41^ under the realm of vibrational strong-coupling conditions.

### 4.1 Modification of Photoreactions and Photophysical Properties under Electronic Strong Coupling

#### 4.1.1 Photochemical Reactions

**Figure 9:**
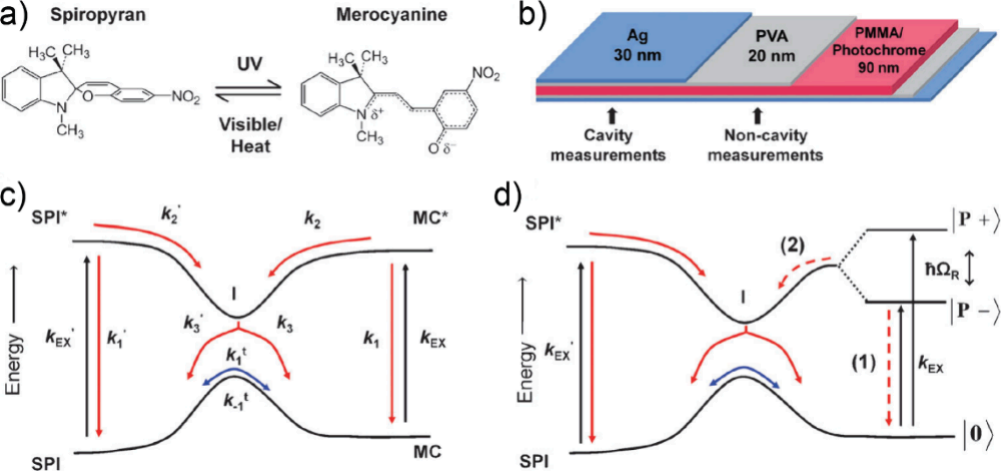
Schematic of photoisomerization reaction modified by ESC. (a) Molecular structures of spiropyran (SPI) and merocyanine (MC) and their isomerization reactions. (b) Structure of the system. Note that cavity and noncoupled measurements were done concurrently on the same film. (c) Diagram of the energy landscape outside of the cavity connecting the two isomers in the ground and first excited state where *k*_EX_ and *k*_EX′_ are the rates of photoexcitation and the others are the rates of the internal pathways. (d) Diagram of the energy landscape under VSC, connecting the two isomers in the ground and first excited state, with modification of the MC states by strong coupling and the appearance of the polariton states |P+> and |P–>, separated by the Rabi splitting *ℏ*Ω_R_. Reproduced with permission from ref (42). Copyright 2012 Wiley.

The inaugural demonstration of the impact of strong coupling on chemical reactions was a photoisomerization reaction between spiropyran (SPI) and merocyanine (MC)^42^ (Figure 9a–b). In 2012, Ebbesen and co-workers observed that when the F–P cavity resonance aligned with the electronic transition of MC, the photoisomerization from MC to SPI experienced deceleration. They further quantified the MC concentration from the UV–vis spectra using the transfer matrix model (TMM). Outside the cavity, the reaction was described as follows: when MC was in the photoexcited state, it encountered a conical intersection, leading to a choice between reverting to the ground state of MC or transition to SPI (Figure 9c). In contrast, under electronic strong coupling (ESC), when MC formed polaritons, these polaritons functioned as a rapid conduit, depleting the photoexcited LP into the ground MC state before relaxation to the conical intersection (Figure 9d). However, within this explanation, the role of dark reservoir modes was not directly considered. This work has been reproduced recently^174^ by a different group independently.

Recently, a few new results have emerged, further substantiating the enhanced photostability within a few other solar materials. Shegai and co-workers^175^ exhibited when strong coupling of J-aggregates with a Ag plasmonic nanoprism conferred greater photostability, a character that was further improved with larger Rabi splitting and red detuning. Their comprehensive analysis included dark reservoir modes, concluding that a swift dark-modes-to-LP relaxation was crucial for achieving photostability. However, to quantify the photostability was challenging, as many factors, such as optical filtering effect, could influence it when the sample was encapsulated in cavities. In a separate study, Noginov et al.^176^ meticulously accounted for all pertinent factors, encompassing radiation strength and local field enhancements, revealing similar improved photostability in a different photopolymer, 2,5-poly(3-hexylthiophene) (P3HT), in an F–P cavity, composed of Ag-coated optics. The author postulated that reversed intersystem crossing played a role, a mechanism related to ESC-modified singlet-to-triplet conversion, as discussed in section 4.1.4. Notably, the quantum yield of another ESC-mediated photoisomerization reaction—norbonadiene to quadricyclane when strongly coupled to an F–P cavity composed of Al-coated optics—was quantified, revealing that the photoisomerization yield remained low only upon exiting the LP states.^177^ This observation implies that the photon leakage rate must outcompete relaxation from LP to dark reservoir modes in order to mitigate the isomerization quantum yield. Another work reported that ESC can promote photodimerization over alternative reaction pathways for TIPS-Tc^178^ in an F–P cavity composed of Ag-coated optics.

Despite realization of the cavity-modified photochemical reactivity, an alternative explaination on that nonpolaritonic factors play a key role remains. Very recently, Thomas et al.^179^ reproduced the pioneering work of cavity-modified isomerization experiments done by Ebbesen and co-workers in 2012.^42^ In their comparative analysis of photochemical reactivity within a metallic cavity versus a control setup where one metallic cavity mirror was removed, the researchers concluded that strong coupling might not be the determining factor that influenced isomerization reactivity. Instead, they noted that reactivity enhancement could be achieved through increased UV absorption in the MC-SPI system in the cavity. Thus, the effects could be photonic, instead of polaritonic. This discovery opens up an alternative approach to investigating the effects of strong coupling on reactivity in cavity photochemistry.

In summary, there have been only a handful of reports on photochemical reactions under ESC, collectively underscoring the notion that explicit photoexcitation of polaritons, especially the LP, could alter the reaction yields and selectivity. Yet, it is crucial to carefully distinguish these effects from nonpolaritonic influences when exploring cavity-modified photochemistry. To achieve this, more extensive and thorough experimental and theoretical research is necessary in the future.

#### 4.1.2 Excitonic Energy Transfer

**Figure 10:**
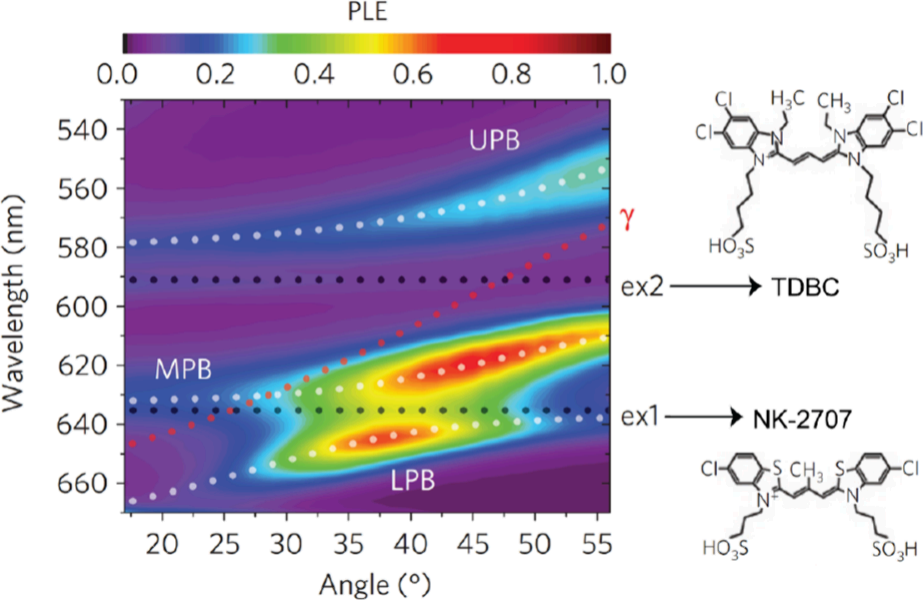
Angle- and wavelength-dependent photoluminescence excitation (PLE) signal recorded at *k*_||_ = 0 on the lower polariton branch (LPB). A significant emission at LPB upon exciting UPB suggested energy transfer between them. Reproduced with permission from ref (43). Copyright 2014 Nature Publishing Group.

Given the inherent delocalized character of polaritons, embraced from the photon properties, polaritons could foster energy transfer between molecules. Lidzey and co-workers^43^ made the pioneering demonstration of polariton-mediated exciton energy transfer between J-aggregates that were strongly coupled to a cavity mode. Their experiment used cyanine dyes 5,6-dichloro-2-[[5,6-dichloro-1-ethyl-3-(4-sulfobutyl)-benzimidazol-2-ylidene]-propenyl]-1-ethyl-3-(4-sulfobutyl)-benzimidazolium hydroxide (TDBC) and 5-chloro-2-[3-[5-chloro-3-(3-sulfopropyl-2(3*H*)-benzothiazolylidene]-2-methyl-1-propenyl]-3-(3-sulfopropyl)benzothiazolium hydroxide (NK-2707). These dyes, both strongly coupled to an F–P cavity mode, composed by two Ag-coated optics, constituted a three-polariton system encompassing LP, MP and UP. In this scenario, UP predominately functioned as the energy donor, while LP was the acceptors. The authors harnessed photoluminescence excitation (PLE) spectroscopy to scrutinize the energy transfer. By fixing the PL detection wavelength at LP emission and scanning the excitation wavelength (Figure 10), they found a non-negligible amount of PL from LP states upon UP excitation. As explained previously, UP and LP shared the photonic component but not the electronic states of the matters. Thus, the presence of PL from LP states while exciting UP distinctly substantiated the notion of the excitonic energy transfer facilitated by polaritons. The MP was considered as a conduit to transfer energy within this intricate arrangement.

The Ebbesen group later further validated the polariton-enabled excitonic energy transfer and its temporal dynamics using transient absorption spectroscopy.^131^ In this system, TDBC was used as the donor and another molecule 1-(3-sulfopropyl)-2-(2-{[1-(3-sulfopropyl)naphtho[1,2-*d*]thiazol-2(1*H*)-ylidene]methyl}-1-butenyl)naphtha[1,2-*d*]thiazolium hydroxide (BRK) served as the acceptor, and they both strongly coupled to an F–P cavity made of Ag coated optics. In another notable contribution, the same group, using the same systems, elegantly showcased that energy transfer could approach a remarkable 37% efficiency, even when the donors and acceptors were separated by a spacer exceeding 100 nm thickness. This compelling finding underscored that the energy transfer can transcend spatial barriers and exhibit long-range capability^44^ (Figure 3c).

**Figure 11:**
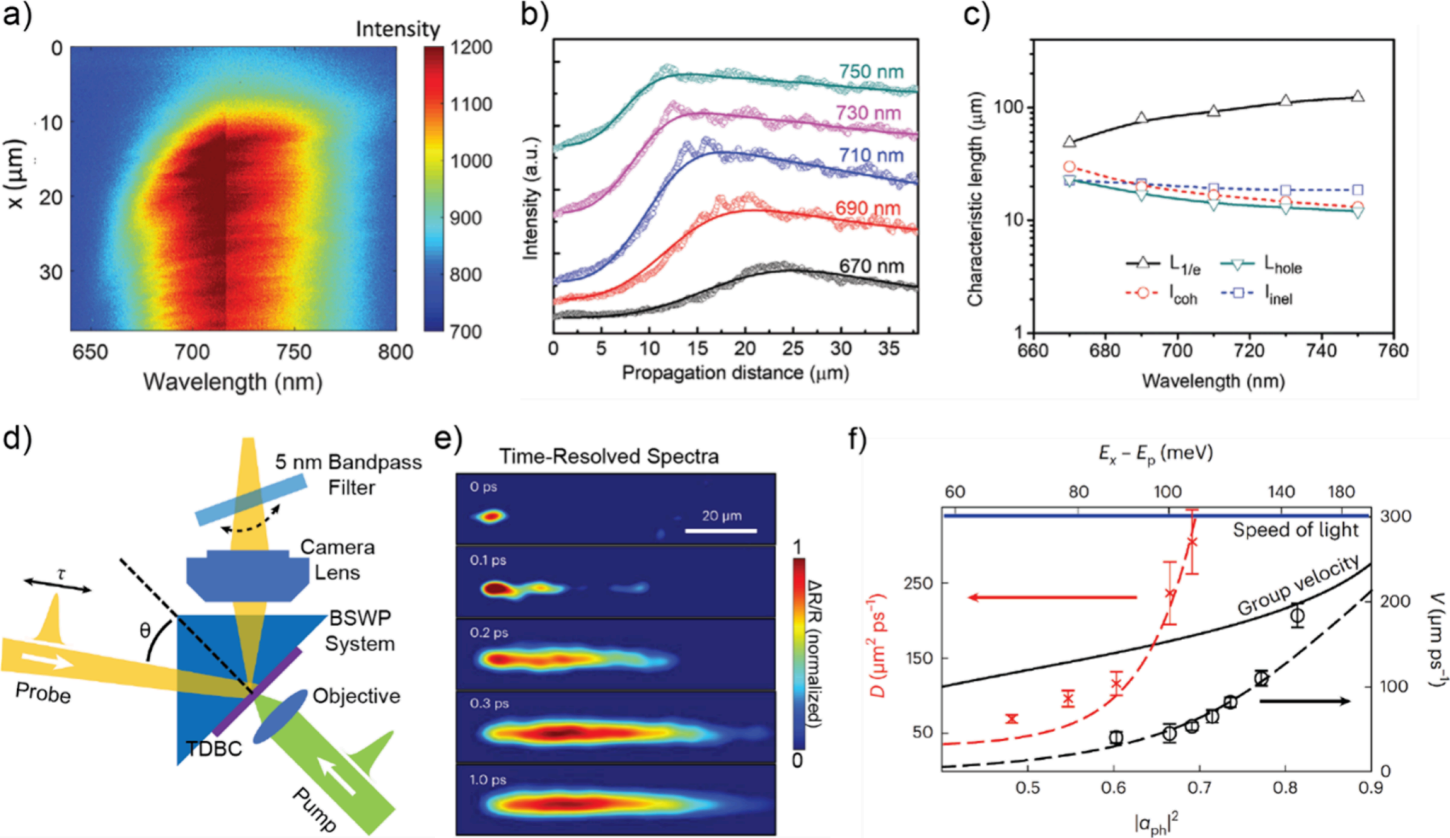
Polariton energy propagation. (a) Energy-resolved propagation image of polariton upon photo-excitation. The vertical break at 720 nm results from combining two images collected in two sequential wavelength ranges. (b) Five propagation profiles at wavelengths of 670, 690, 710, 730, and 750 nm. The calculated distributions (solid lines) are fitted to the measured data (circular dots). (c) Comparison of the coherence length, l_coh_; phase-breaking length, l_inel_; hole size, L_hole_; and total propagation length L_1/e_ versus wavelength. (d) Schematic sketch of the optical configuration used in the pump–probe microscopy experiments on exciton in TDBC molecular layer mixed with Bloch surface waves. (e) Representative snapshots of the time-resolved microscopy, showing the gradual expansion of the polariton cloud. (f) Polariton transport parameter variation as photonic component increases, where the red cross points and black circle points are the experimental diffusion coefficients and ballistic expansion, respectively, compared to the theoretical group velocity (solid black line). (a–c) Reproduced with permission from ref (180). Copyright 2020 Wiley. (d–f) Reproduced with permission from ref (12). Copyright 2023 Nature Publishing Group.

Other photonic modes, such as plasmonic–photonic hybrid cavities, have also emerged as enablers of energy transfer.^45^ In particular, the utilization of Block surface wave (BSW) as the photonic modes can extend the energy transfer reach. Recent advancements even revealed that the BSW supported by a DBR can facilitate energy transport over 100-μm distances^180^ within a single material, tetraphenyldibenzoeriflanthene (DBP), highlighting the immense potential of these systems (Figure 11a–c). Another work done by Balasubrahmaniyam et al.^12^ demonstrated that the transportation of Frenkel exciton in an organic semiconductor (TDBC) can be enhanced via strong coupling (SC) to the BSW (Figure 11d). Their pump–probe microscopy results clearly indicated a mobility transition from diffusive to ballistic mode with two-thirds of the speed of light when its photon component is increased to 0.7 or higher (Figure 11e and f). In that work, the competing mechanisms between disorder and long-range correlations has been discovered during such mobility transition process, depicting a clear pathway to engineer SC-mediated long-range transportation of molecular excitations.

**Figure 12:**
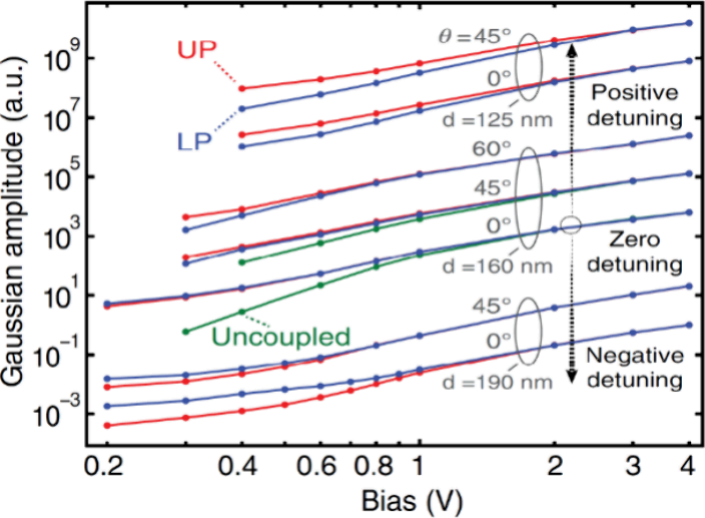
Polariton modification to charge transport. Bias dependence of the UP (red), LP (blue), and uncoupled polaron (green) peak amplitudes, along with analogous data sets obtained for two additional cavities with different thicknesses as indicated in the plot. Each data set is normalized at 4 V and vertically offset for clarity. The uncoupled polaron contribution can only be reliably discerned in the spectra near zero detuning. Reprinted with permission from ref (48). Copyright 2020 American Physical Society.

#### 4.1.3 Charge Transfer and Transport

ESC not only can facilitate energy transfer but also plays a crucial role in charge transfer and transport. Gómez and co-workers showcased that the photocurrent can be enhanced when the TiO_2_ semiconductor waveguide modes and the plasmonic modes of Ag gratings were strongly coupled. They attributed this enhancement to an increased electron injection probability, facilitated by the radiative decay.^47^ More recently, the Giebink group delved into how polaron polaritons influence photoconductivity.^48^ The sample was a sandwich-type device consisting of indium–tin–oxide (140 nm)/30 vol % MoO_3_:TAPC (4,4′-cyclohexylidene-bis[*N*,*N*-bis(4-methylphenyl)benzenamine) (160 nm)/Ag (20 nm). They revealed a modest enhancement of photoconductivity under low bias conditions when exciting polaron polaritons, whereas no discernible differences were observed between polariton-mediated and uncoupled polaron systems under high bias (Figure 12). This outcome was explained by the fact that at low bias, the polaron polaritons can undergo spatial delocalization, thereby effectively sampling a broader range of sites. The enhanced charge transfer process outcompetes the dephasing dynamics, resulting in an extended thermalization length of the Onsager theory. However, under high bias, the gains conferred by polariton delocalization were outweighed by the improved intrinsic photoconductivity. Similar trends of enhanced photoconductivity were also reported in p-type semiconductors, P3HT,^181^ deposited on a Ag hole array. These investigations collectively highlight the multifaceted role of ESC in influencing charge transfer and conductivity properties.

**Figure 13:**
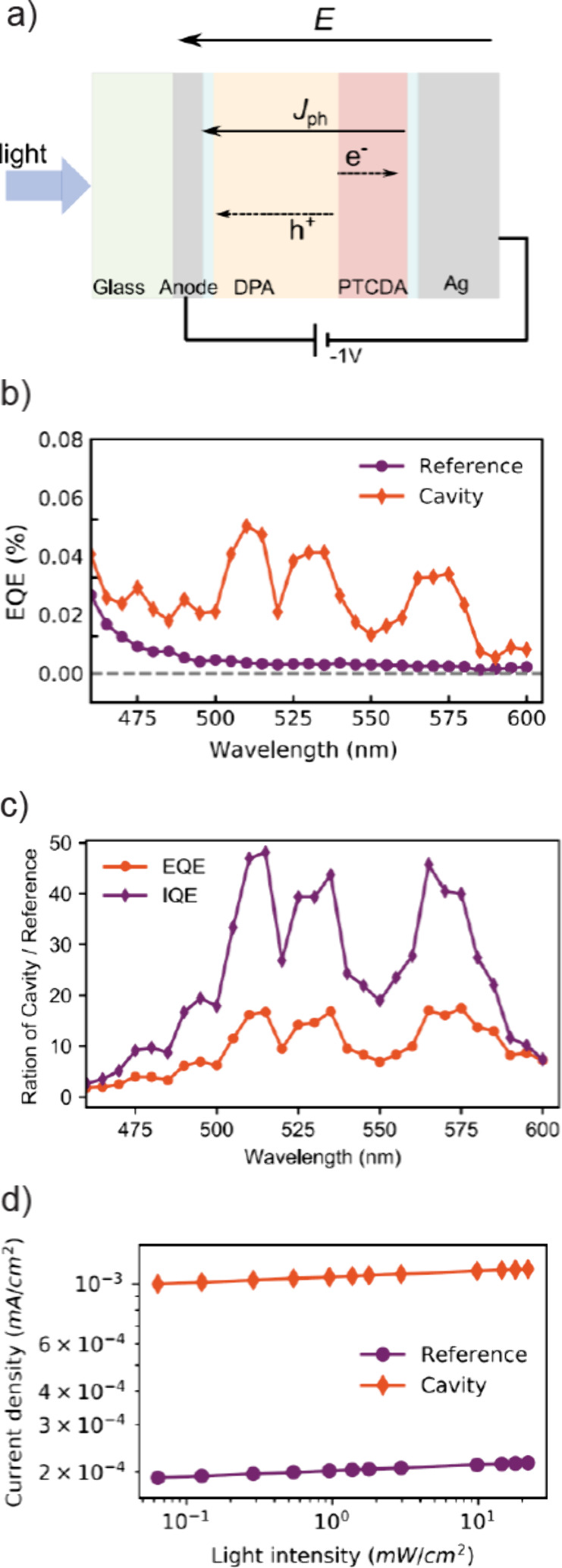
Enhanced photocurrent under ESC. (a) Device structures under electronic strong coupling (ESC). The anode for the cavity device is 20 nm Ag and 110 nm ITO for the reference device. Spacer layers of mCP and TmPyPB were 5 and 10 nm, respectively. (b) External quantum efficiency (EQE) of the photodiode under −1 V bias at short circuit condition. (c) Ratio of EQE and internal quantum efficiency (IQE) of the cavity and reference devices. (d) Log–log plot of light intensity (475 nm) dependent photocurrent in cavity and reference devices. Reprinted with permission from ref (182). Copyright 2021 Nature Publishing Group.

Deeper insights into the enhanced charge transfer have emerged through studying type-II heterojunctions under ESC condition.^182^ The researchers designed an ESC system using type-II heterojunctions composed of 2,6-diphenylanthracene (DPA) as the donor and perylene-3,4,9,10-tetracarboxylic dianhydride (PTCDA) as the acceptor, in a Ag-coated F–P cavity, where charge transfer occurs (Figure 13a). Notably, the authors observed an enhancement in exciton harvesting, as evidenced by the increased photocurrent quantum efficiency (Figure 13b–d). They attributed this enhancement to an improved conversion efficiency from excitons to charge transfer exciton states, because other factors, such as free charge career mobility, should be decoupled from the cavity.

**Figure 14:**
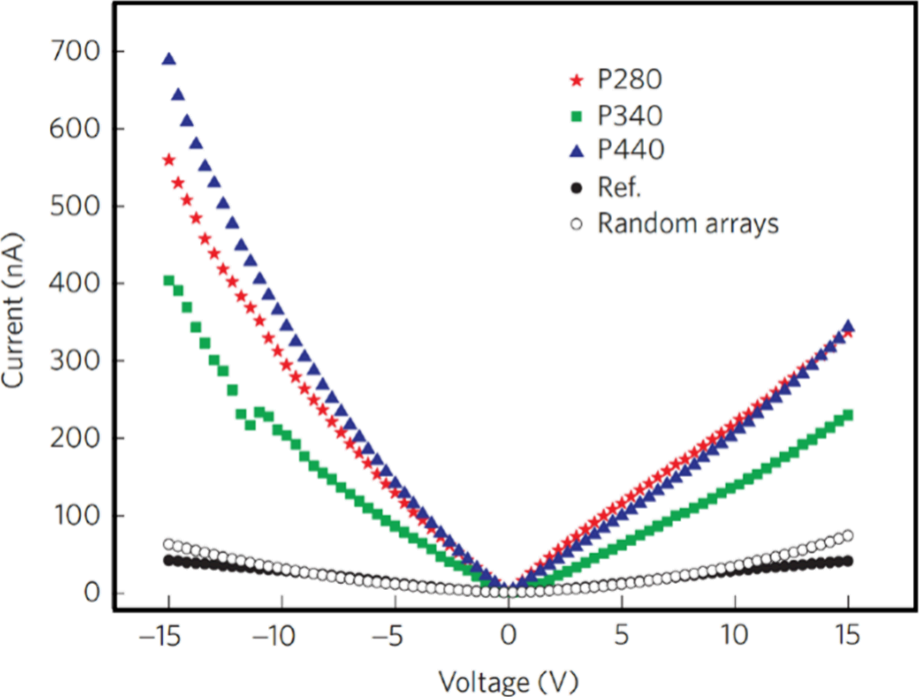
Enhanced conductivity by ESC in the absence of photoexcitation. I–V curves as a function of the hexagonal array at selected periods for the configuration used to measure conductivity using surface plasmon resonances generated by the hexagonal array milled in a 100-nm-thick Ag film, 50-μm wide, deposited on a glass substrate. Reprinted with permission from ref (46). Copyright 2015 Nature Publishing Group.

While the polariton enhanced photocurrent is intuitive, it is intriguing to note that there have been reports of enhanced conductivities in semiconductors under ESC without any photoexcitation, which comes as a more surprising revelation.^46^ By achieving strong coupling between an n-type organic semiconductor of three aromatic diimide molecules and plasmonic metal (Ag and Al) hole arrays with various geometric parameters (P280, P340, P440, etc.), the authors conducted IV curve measurements (Figure 14) that unequivocally showed accelerated charge transport. Subsequent investigations showed similar acceleration in p-type semiconductor,^181^ transition metal dichalcogenide (TMD) systems^183^ and even magneto systems.^184^

**Figure 15:**
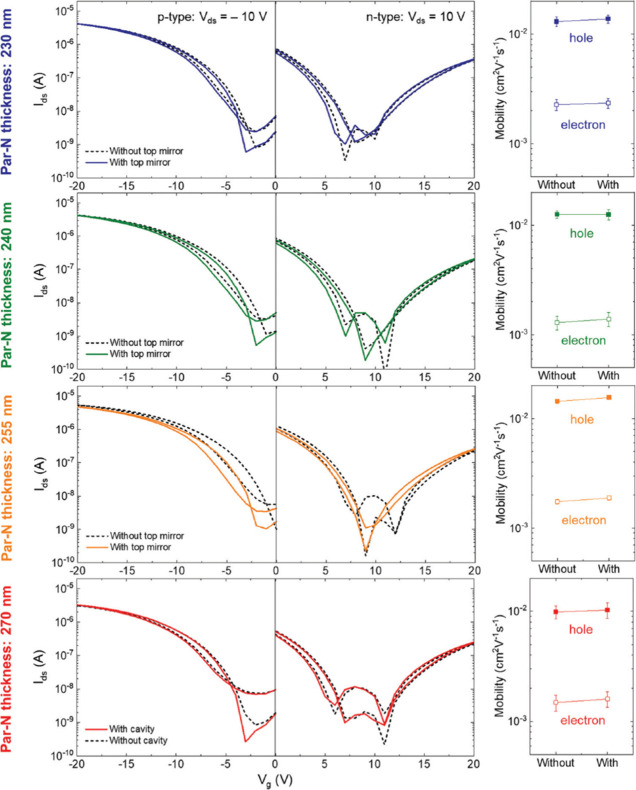
Negligible modification of I–V curve under ESC without photoexcitation. Transfer curves in p-type and n-type operation modes for the transistors with and without top mirror (left panel). The different rows correspond to different parylene–N (Par-N, top spacer) thicknesses, as indicated in the figure. Hole and electron mobility values extracted from the transfer curves (right panels). Error bars represent the standard deviation of the mobility values for multiple devices. Reprinted with permission from ref (185). Copyright 2021 Royal Society of Chemistry.

However, it is important to acknowledge a counterpoint in the form of a negative result, where the anticipated enhancement in charge transport was not observed under ESC (Figure 15). Such an example is the metal-free phthalocyanine strongly coupled to a metal-coated F–P cavity. In this instance, the authors attributed the absence of enhancement to the fact that the coupling strength (between 70 to 150 meV) had not reached a level considered sufficiently high,^185^ compared to the 700 meV coupling strength in the plasmonic structure results.^46^

The mechanism behind the conductivity enhancement under ESC without photoexcitation remains enigmatic, as it involves numerous unaltered dark reservoir modes that could seemingly preserve conductivity levels. In this respect, it resembles the thermally activated VSC-modified reactions, which we will delve into in section 4.2. One possible avenue could be a connection to the altered work function under ESC.^186^ Another compelling explanation might stem from recent studies on polariton transport, suggesting a rate of transfer from dark reservoir mode to polariton that surpasses conventional assumptions.^14^ This heightened transfer rate potentially prompts dark reservoir modes to more readily transition back to polariton states, which are delocalized and could elevate conductivity. Despite these theoretical insights, direct measurement of the transfer rate from dark reservoir to polaritons remains notably absent.

#### 4.1.4 Spin Dynamics

**Figure 16:**
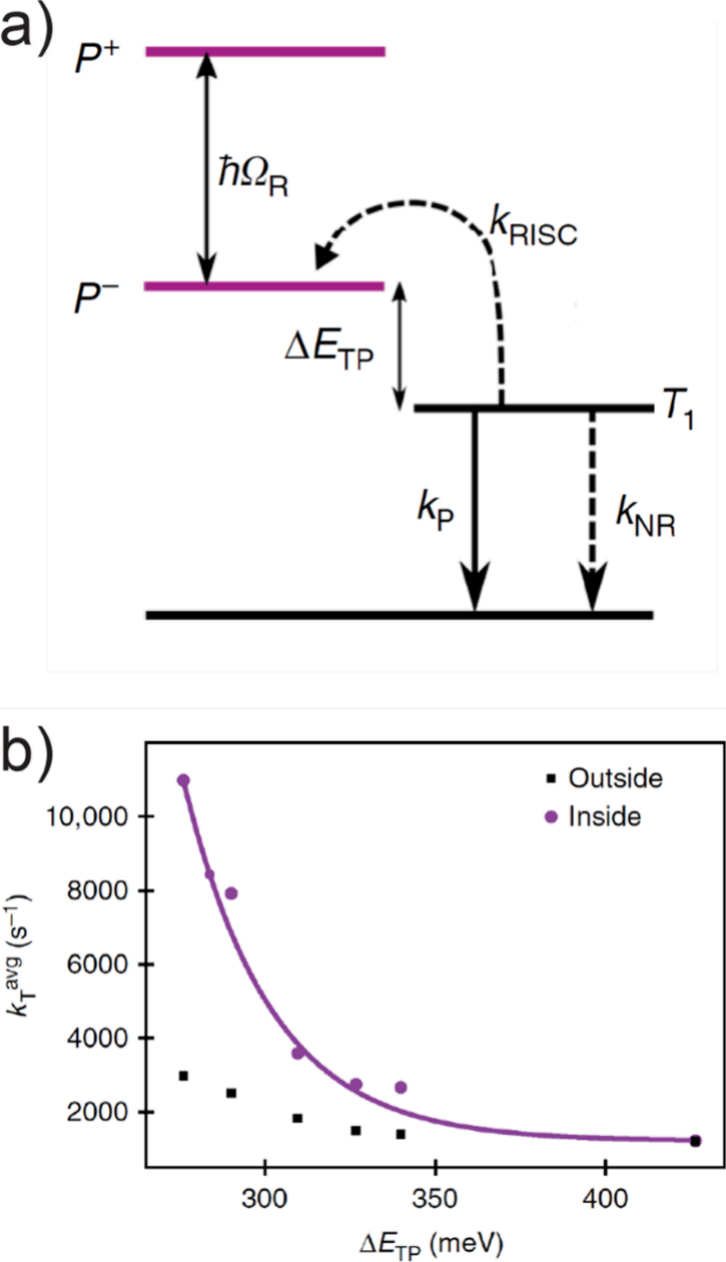
Modified reversed intersystem crossing by ESC. (a) Energy diagram describing the kinetics of the triplet-state depopulation pathways inside a cavity. *k*_p_, *k*_NR_, and *k*_RISC_ are the rates of phosphorescence, nonradiative decay, and reverse intersystem crossing, respectively. Δ*E*_TP_ is the energy difference between T_1_ and P^–^. (b) Increase of the average total and the fitted rate constant of the triplet-state depopulation (*k*^avg^_T_) inside the cavity as a function of the energy difference between T_1_ and P^–^ (Δ*E*_TP_). The rate constants outside the cavity with equivalent concentration are shown for comparison. Reprinted with permission from ref (49). Copyright 2018 Nature Publishing Group.

Another captivating area that ESC has been reported to exert control is in spin dynamics, particularly the conversion between singlet and triplet states, as well as triplet annihilation. Börjesson and co-workers^49^ pioneered the observation of delayed PL emission in their time-resolved measurements of a sandwiched structure of Ag (300 nm)/erythrosine B/Ag (20 nm), which they attributed to an expedited reversed intersystem crossing (RISC). The rationale was that LP diminished the energy gap between the singlet and triplet states, thereby lowering the energy barriers associated with RISC (Figure 16).

**Figure 17:**
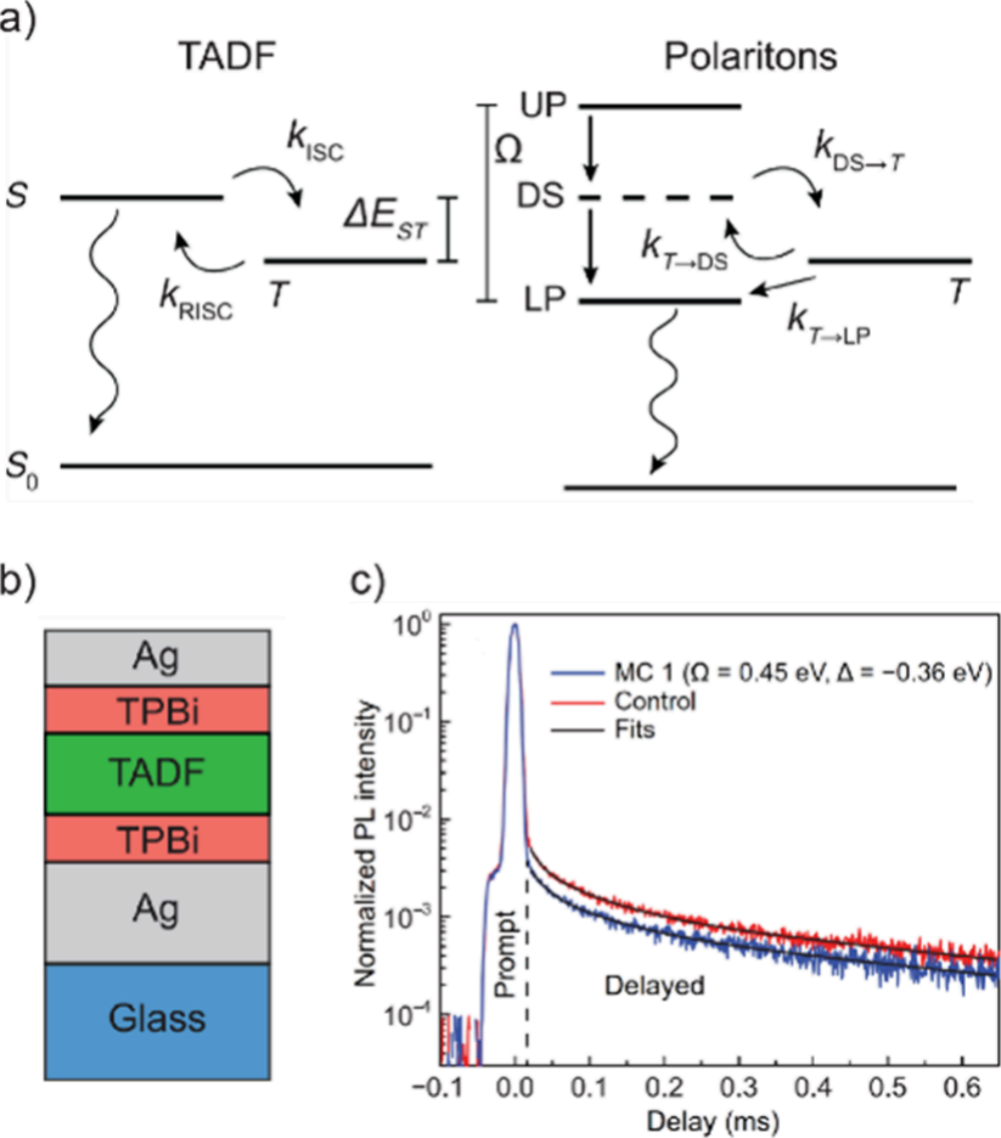
Lack of modification of reversed intersystem crossing by ESC. (a) Electronic energy levels and rate constants for the TADF material, and polaritons and dark modes. (b) Microcavity structure consisting of a Ag bottom mirror (100 nm), a Ag top mirror (30 nm), TBPi buffer layers (10 nm each), and a TADF layer consisting of either neat 3DPA3CN with a thickness of 70 nm (MC Neat) or codeposited TPBi-3DPA3CN (55 to 45% by volume) with a thickness of 64 nm (MC 1) or 81 nm (MC 2). (c) Transient PL decays for the LP (blue line) and control film singlet (red line) at a collection angle of 0° for MC 1. Reprinted with permission from ref (50). Copyright 2019 American Association for the Advancement of Science.

Kéna-Cohen and co-workers explored a different singlet–triplet system, composed of 1,3,4-tris(4-(diphenylamino)phenyl)-2,4,6-tricyanobenzene (3DPA3CN, ∼70 nm) sandwiched between two Ag-coated optics, yet did not observe delayed PL emission or alternations in RISC^50^ (Figure 17). They proposed that while the energy barrier was reduced, the polariton wave functions spanning *N* molecules introduced a  entropic factor that hindered the acceleration of the RISC rate constant. They also underscored that the PL dynamics are highly sensitive to the sample preparation conditions, noting that residual molecular oxygen in microcavity optics could also influence the dynamics. This view gained support through ultrafast transient absorption measurements on an ESC system composed of triisopropysilylacetylene pentacene (TIPS-Pc) sandwiched between Ag-coated mirrors, as reported by Menon, Sfeir and co-workers.^187^ In their work, after meticulous consideration of spectral features using the Rabi splitting contraction model, the authors concluded that, owing to the relatively slow transition from the S_1_ state to LP (∼100 ns)^−1^, compared to the singlet fission rate (∼50 ps^–1^), the existence of polaritons had minimal impact on singlet fission dynamics. Furthermore, the authors also highlighted that unlike from transient spectra of systems without a cavity, certain spectral features near polariton resonances might be attributed to alternative phenomena, such as Rabi splitting contraction, instead of polariton features, i.e., changes in polariton population.

**Figure 18:**
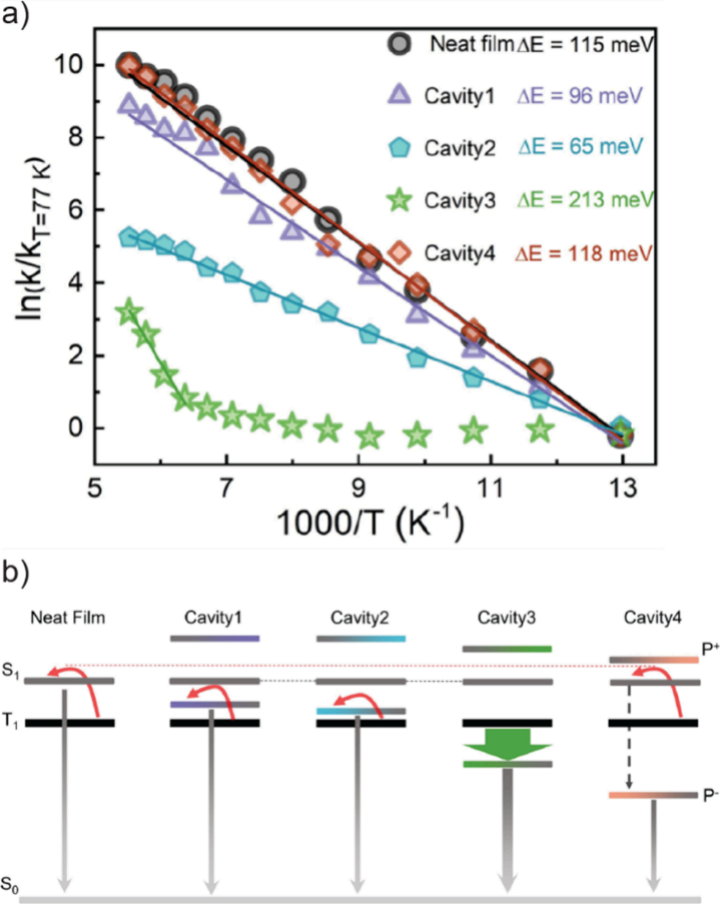
Subtle detuning conditions for ESC modifying reversed intersystem crossing. (a) Arrhenius analysis with calculated activation energies for delayed P^–^ (or singlet) emission inside and outside cavities. (b) Schematic illustration of the dynamics of the triplet to polariton (or singlet) transition at low temperatures. Red arrows represent thermally activated, and green represents barrier-free RISC. The dashed black arrow indicates a relaxation from S_1_ to P^–^, and the gray one represents delayed emission from S_1_ or P^–^. Reprinted with permission from ref (188). Copyright 2021 Nature Publishing Group.

In a very recent investigation, Börjesson and co-workers reported another case of barrier-less RISC modified by ESC.^188^ The sample is DABNA-2 sandwiched between two Ag-coated optics. They demonstrated that achieving the condition of barrier-less RISC was very intricate, as it was only accomplished within a specific detuning condition (Figure 18). This observation highlights the delicate nature of the conditions required to employ ESC for accelerating RISC. Importantly, the theoretical underpinnings of these phenomena remain to be further clarified and unified.

Beyond RISC, other spin dynamics, such as triplet annihilation, could also be influenced by ESC. Notably, Musser, Clark, and co-workers reported a distinct phenomenon in their study involving a film of diphenylanthracene/Pt-porphyrin/polystyrene blends (ratio of 50:1:15) under ESC condition.^51^ They observed the emergence of a new PL signal at longer time delays, while the early time dynamics remain unchanged. Their findings are suggestive of a novel pathway for triplet annihilation, where a triplet dimer (TT) is converted into an LP. A similar occurrence of triplet annihilation was reported a year later by a different group, focusing on a polariton system composed by DPP(PhCl)_2_ and triplet sensitizer PtTBTP.^189^ Considering the significance of triplet annihilation in energy and photonic applications in recent years, leveraging ESC to enable or modify this process could have a transformative impact, contingent upon a comprehensive and mechanistic understanding that can be attained in the near future.

Recent theoretical investigations have shed light on the optimal condition for singlet fission. Climent and co-workers surveyed the conditions that can make polariton promote this process.^190^ They concluded that in cases where singlet fission is inherently endothermic, the energetics of polaritons can reverse the process and render it thermodynamically favorable. They pointed out that the triplet–triplet (TT) states were also a dark state, sharing a similar dependence on the scale of the number of entities participating in the delocalized state (*N*) as singlet dark modes. Thus, relaxation to singlet dark modes and TT states can occur at similar time scales and compete with each other. Moreover, the presence of a large Rabi splitting can suppress the impact of dark singlet modes, even with their large density of states, in comparison to polaritons. Lastly, the researchers examined the influence of disorder and noted that when the Rabi splitting was not large enough, disorder could make the dynamics of polariton-enabled singlet fission similar to those of the uncoupled case, potentially explaining certain experimental results. However, predictions from the theoretical framework still require experimental validation.

#### 4.1.5 Challenges and Opportunities

The body of experimental evidence has supported the notion that ESC holds the potential to modify photophysics, yielding changes that can range from subtle to orders of magnitude. This prospect casts a promising future on the application of molecular polaritons for controlling and fine-tuning photophysical and photochemical properties. However, amidst the positive outcomes, instances of null results warrant equal consideration. These cases underscore the subtleties inherent in the conditions necessary for ESC to influence photodynamics. A closer examination of these results, complemented by detailed reports and comparisons from different groups, would certainly enhance our understanding.

Furthermore, the advancement of this nascent field could greatly benefit from continuous theoretical development, specifically geared toward simulating the collective coupling regime rather than the ideal single-molecule coupling regime. This difference, epitomized by the Tavis–Cummings versus Jaynes–Cummings models, hinges on the distinction that the former accurately portrays the energetics of dark modes, which become prevalent in the collective coupling regime. In most photophysics and photochemistry experiments, selective pumping onto the polariton modes serves to initialize the system in the delocalized modes. Nonetheless, an intriguing question remains: do these delocalized modes quickly relax to the dark modes before the anticipated modified photophysical or photochemical phenomena occur?^50^ If so, the role of polariton modes becomes mysterious. It is conceivable that the DOS of dark modes are not as large as the prediction from the Tavis–Cummings model, or alternatively, the rates of transition from dark modes to polaritons might surpass theoretical expectations, as some experimental results have intimated.^14^ A comprehensive theoretical framework that can offer predictive insights into the influence of ESC on photochemical and photophysical activities necessitates further rigorous experimental and theoretical investigations.

### 4.2 Modifying Thermally Activated Chemical Reactions Using Vibrational Strong Coupling

#### 4.2.1 Thermally Activated Reactions under VSC Conditions

Although introduced at a later stage, the notion of using VSC to manipulate the energy levels of specific chemical bonds, thereby impacting chemical reactions under thermally activated conditions, has generated considerable disruption and ignited both enthusiasm and debates within the field.^16,17,76,191^ Comparable to the effects of ESC on photochemical reactions, VSC-modified reactions also hinge on the creation of polariton states that emerge from the hybridization between molecular vibrations and photon cavity modes. However, a remarkable difference between these two phenomena lies in that the reactions modified by VSC necessitate no external photon perturbations!

**Figure 19:**
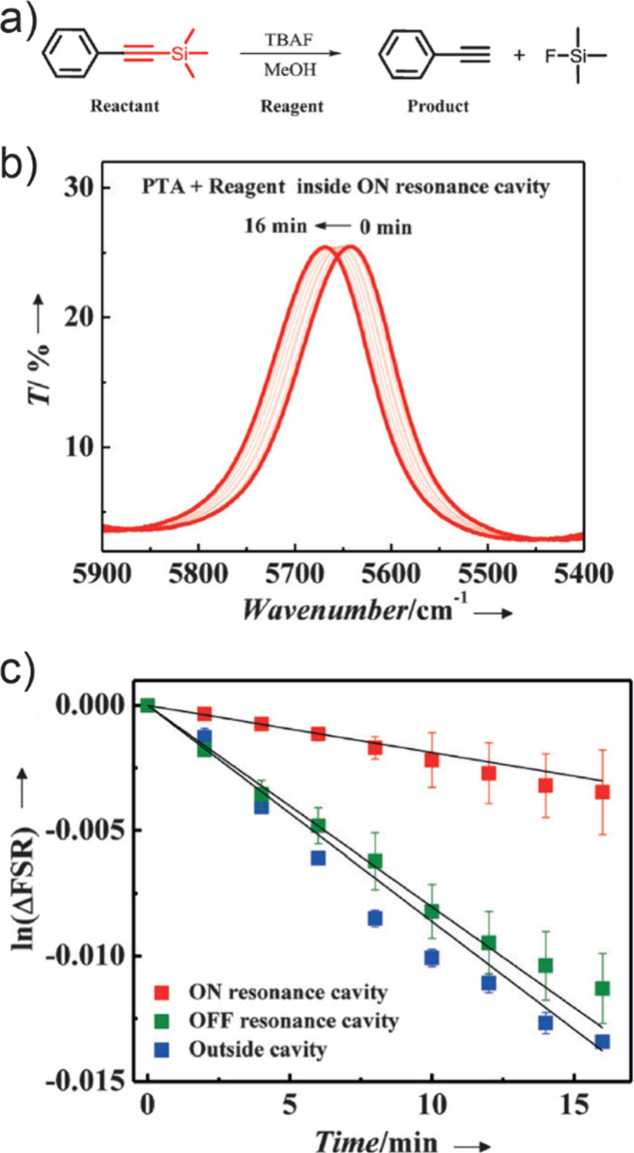
Deceleration of reaction rate under VSC. (a) Silane deprotection reaction of 1-phenyl-2-trimethylsilylacetylene. (b) Reaction rate monitored by the spectral shift of the higher-order cavity modes during the reaction (0 to 16 min). (c) Kinetics of the reactions in an on-resonance cavity (red squares), outside the cavity (blue squares), and in an off-resonance cavity (green squares), extracted from the shifts of the higher-order cavity modes. Reprinted with permission from ref (41). Copyright 2016 Wiley.

In 2016, a groundbreaking advancement was made by Ebbesen and co-workers^41^ in the realm of VSC-modified chemistry. This study focused on a silane deprotection reaction of an alkynylsilane, 1-phenyl-2-trimethylsilylacetylene (PTA) (Figure 19a) and utilized the shift of cavity peaks in FTIR spectroscopy as a signature for tracking the reactions (Figure 19b). The underlying rationale was that as reactions occurred after the premixed reactant solution was guided into the liquid flow cell with highly reflective cavity mirrors, corresponding changes in refractive indices can cause shifts in cavity resonant frequencies. Notably, the reaction rate constant was reduced by 5.5 times when the Si–C stretch modes around 860 cm^–1^ were strongly coupled (Figure 19c). As the Rabi splitting increased, the reaction rates decelerated further. Temperature-dependent studies and Eyring analysis indicated that the activation enthalpy increased from 39 to 96 kJ/mol, accompanied by a dramatic elevation in the activation entropy from −171 to 7.4 J/K/mol. Based on these results, the authors concluded that the reactions shifted from an associative transition state initialized by fluorine attacking on the silicon atom to form an intermediate with pentavalent coordination, to a dissociative transition state wherein the S–C bond starts breaking before fluorine attaches to the silicon center.

**Figure 20:**
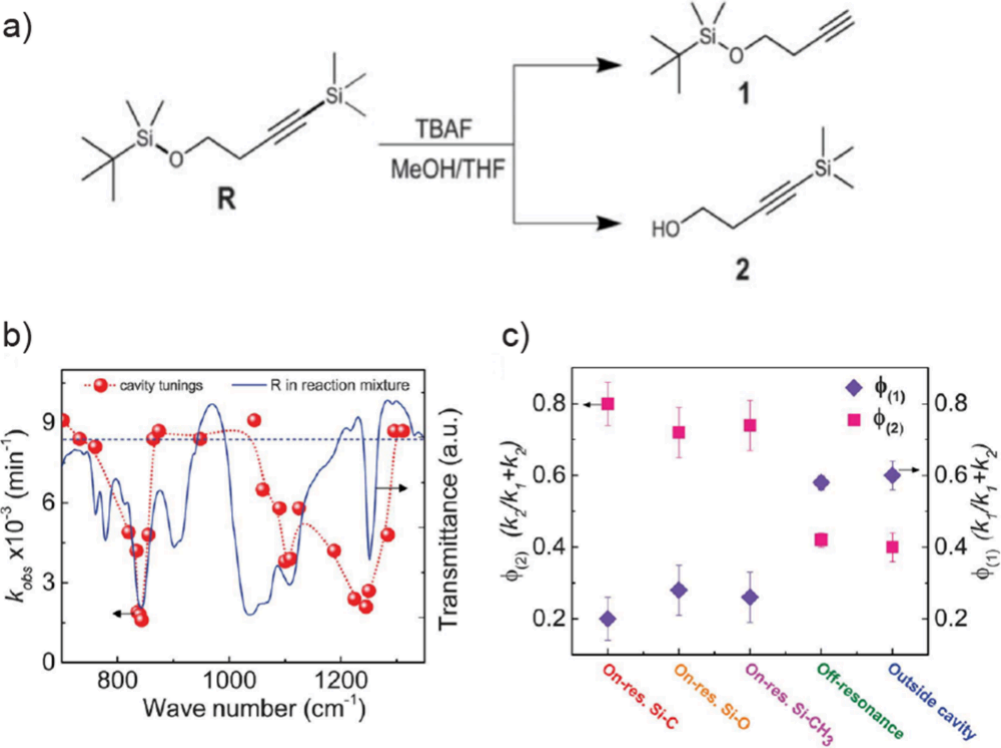
Modulation of reaction selectivity under VSC. (a) Two major silyl cleavage pathways (Si–C scission to form 1, Si–O scission to form 2) for the reaction of R with TBAF in a room-temperature mixture of methanol and THF. (b) Overall reaction rate as a function of cavity tuning for reactions inside the cavity (red spheres). The blue solid line shows the IR absorption spectrum of R in the reaction medium. The red dotted line connecting the spheres is a guide for the eye. The blue dashed line represents the average rate of the reaction outside the cavity. (c) Plot showing the yields of products 1 (ϕ_1_; violet diamonds) and 2 (ϕ_2_; pink squares) under VSC of various vibrational modes of R, together with the off-resonance and outside cavity conditions. The error margin was determined from the standard deviation of a minimum of five experiments in each case. Reprinted with permission from ref (120). Copyright 2019 American Association for the Advancement of Science.

Another pivotal work^120^ was also conducted on a desilylation reaction, wherein the selectivity of cleavage of *tert*-butyldimethyl{[4-(trimethylsilyl)but-3-yn-1-yl]oxy}silane was reported to be controlled by VSC (Figure 20). The authors found that outside the cavity, the reactions favored Si–C bond breaking, with the Si–C and Si–O bond breaking ratio standing at 1.5. A similar ratio was observed when the reactions were conducted within the cavity but detuned from any of the vibrational modes. In stark contrast, under VSC conditions, the reactions preferred Si–O bond cleavage, leading to an overall reduction in reaction rate by 2.5 times. As observed in the early work, these reactions also displayed a high detuning dependence. This study was the first instance in which VSC demonstrated its ability to switch reaction selectivity.

Beyond the initial desilylation reactions, many other reactions have been reported to be influenced by VSC under thermally activated conditions. Importantly, these studies have leveraged diverse analytical techniques, including UV–vis absorption,^126,192^ GC-MS^120^ and NMR,^193^ to meticulously track the progression of reactions. Among these studies is the modification of Prins cyclization between aldehyde/ketone and 3-buten-1-ol,^194^ and charge transfer in the complex formed between trimethylated benzene and iodine. In the later case,^192^ the authors used UV–vis absorption to monitor the equilibrium constants between mesitylene and its iodine complex. They found that the equilibrium shifted toward either reactants or products depending on whether the symmetric or asymmetric methyl groups were under VSC. This work highlighted the significance of molecular mode symmetry in VSC-modified reactions. However, they also reported that the UV–vis absorption cross section changed.

Another notable exploration^193^ delved into the modification of selectivity of the famous Woodward–Hoffman reactions of *cis*-3,4-disubstituted cyclobutene, tracked through NMR spectroscopy. The findings indicated that when CO modes were strongly coupled, the symmetry-allowed cis–trans conformation was promoted. Conversely, when CH bending modes were strongly coupled, the symmetry-forbidden trans–trans products were favored. This outcome once again underscored the pivotal role of symmetry in VSC-modified chemistry.

**Figure 21:**
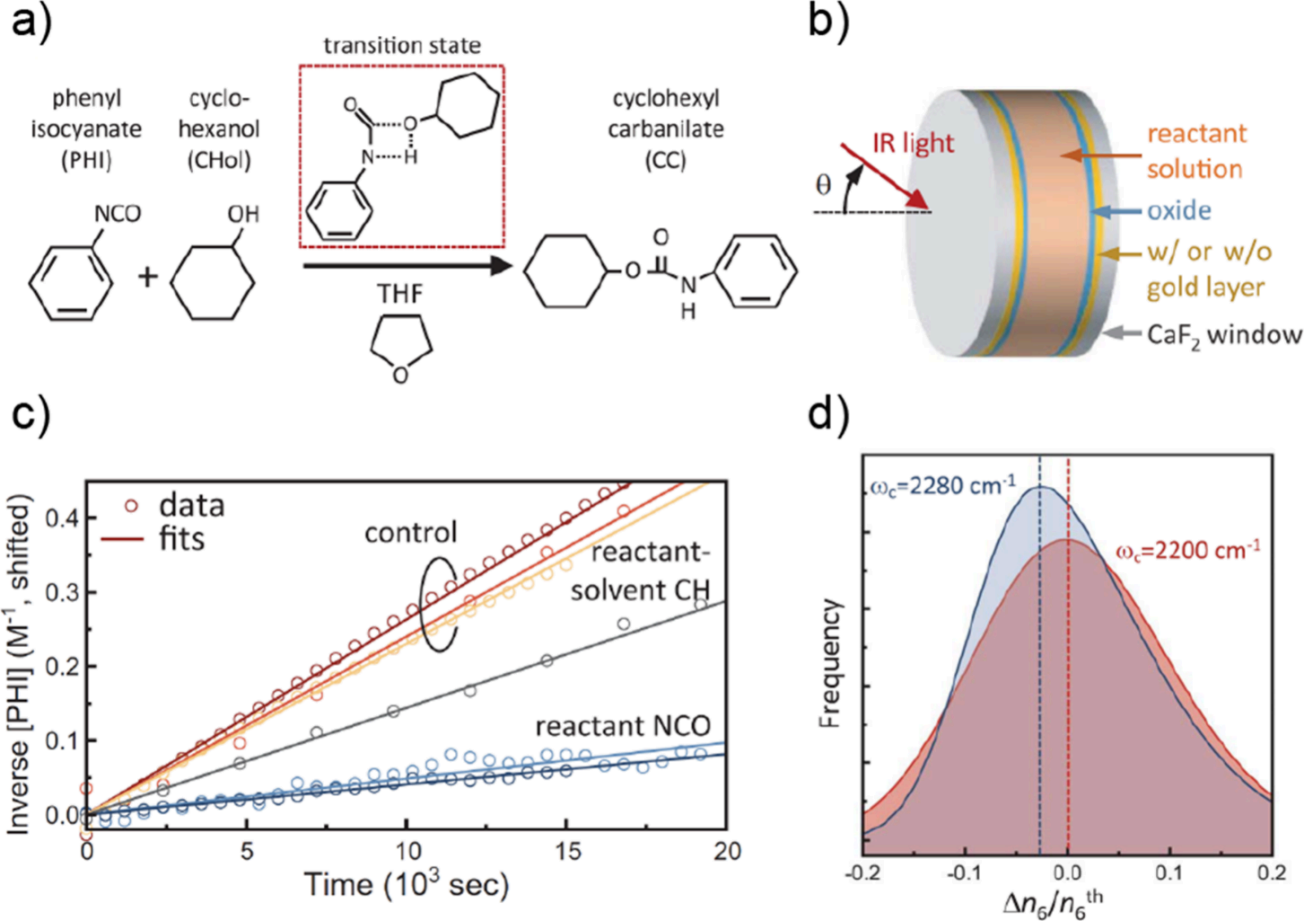
VSC-induced chemical reactivity of an alcoholysis reaction, described by an open quantum mechanical model. (a) The reactants phenyl isocyanate (PHI) and cyclohexanol (CHol) were combined in tetrahydrofuran (THF) to form cyclohexyl carbanilate (CC). (b) A solution was contained between two CaF_2_ windows that were either transparent (for control measurements) or coated with Au/SiO_2_ (for cavity-coupled experiments). (c) Reaction suppression was observed when the cavity was tuned to prominent vibrational modes (CH and NCO modes) compared to the control experimental results, where reaction rate constants were extracted from linear fitting. (d) Cavity-detuning-dependent vibrational population redistribution predicted by the cavity quantum mechanical model. Reprinted with permission from ref (125). Copyright 2023 American Association for the Advancement of Science.

A recent crucial work done by a group of researchers from Naval Research Laboratory^125^ showed that the VSC of the NCO stretch mode could decelerate the alcoholysis of phenyl isocyanate with cyclohexanol that yielded urethane monomers (Figure 21a). Using FTIR to monitor the reaction progress in an F–P cavity (Figure 21b), they found that by tuning the cavity resonance to the CH mode of reactant/solvent or the reactant NCO mode will lead to a chemical reactivity suppression at different levels (Figure 21c). They further developed an open quantum system model to predict the cavity modification of the chemical reactivity. In their model, the cavity detuning dependence on the vibrational distribution of the reactants has been investigated, revealing the deviation of vibrational occupations from canonical Boltzmann statistics induced by stationary light–matter coherence (Figure 21d). They further related such a mechanism to the chemical bonds breaking through a two-body process, providing a comprehensive quantum mechanical insight on VSC-mediated modification of chemical reactivity.

**Figure 22:**
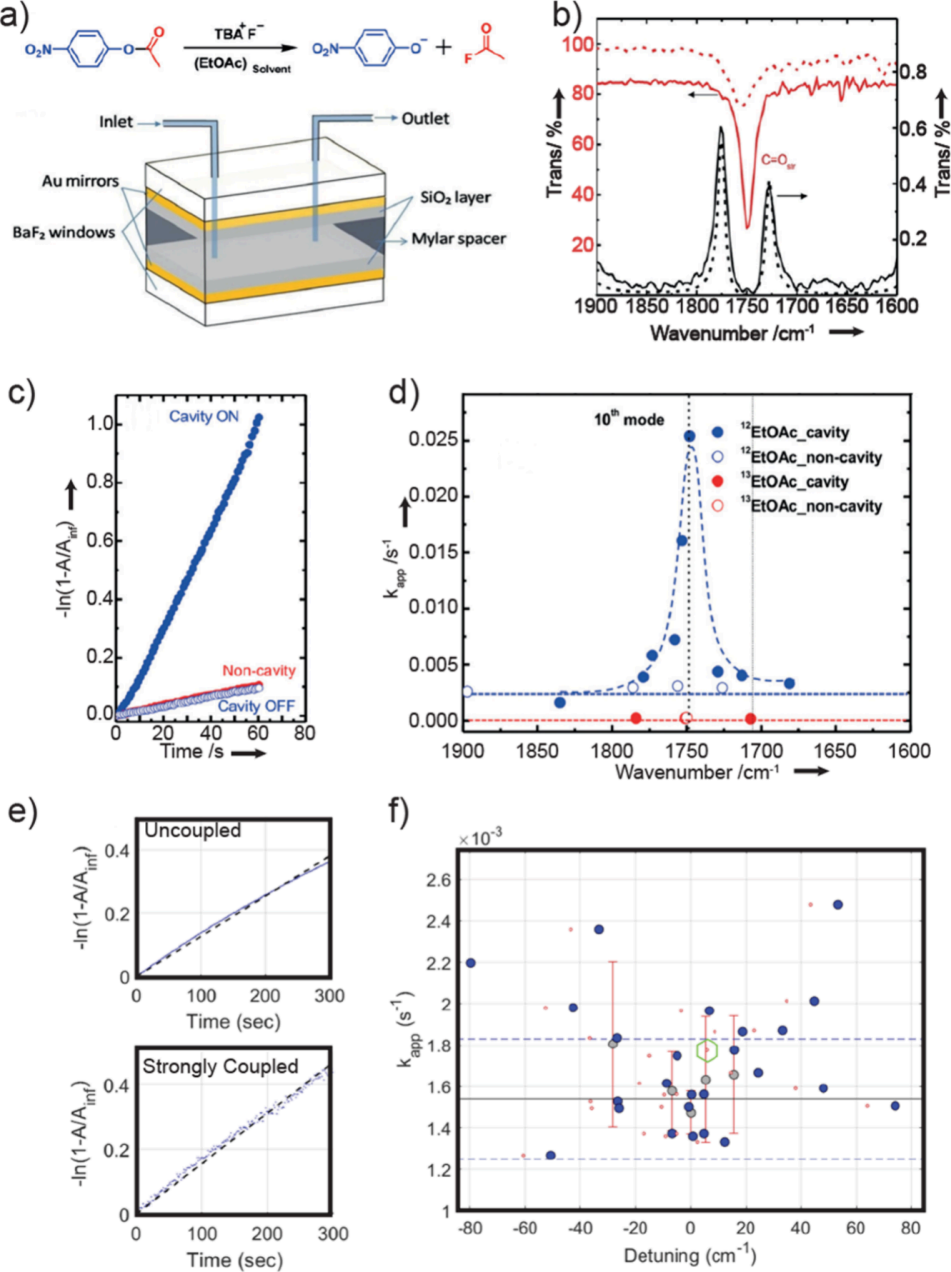
Modulation of hydrolysis under the cooperative VSC regime. (a) Parts of a flow-cell microcavity QED reactor for PNPA hydrolysis in ethyl acetate (EtOAc). (b) IR transmission spectra of 10% EtOAc (red trace) and 0.1 M PNPA (dotted red trace; magnified by factor 100) in hexane. Polaritonic states P+ and P– formed by coupling to the tenth cavity mode (black trace; path length is ,approximately 18 mm) with a TMM simulation (dotted black trace). (c) Pseudo-first-order kinetic traces measured at 407 nm for cavity on-resonance (blue circle; 1.6 × 10^–2^ s^–1^), cavity off-resonance (blue hollow circle; 0.16 × 10^–2^ s^–1^), and noncavity (red circle; 0.18 × 10^–2^ s^–1^) for ^12^EtOAc. (d) Kinetic rates as a function of detuning the cavity (^12^EtOAc, blue filled circles; ^13^EtOAc, red filled circles) and noncavity (^12^EtOAc, blue empty circles; ^13^EtOAc red empty circles). The tenth mode of the cavity overlapped with the carbonyl stretching mode of ^12^EtOAc and PNPA. The dashed curves are guides to the eye. (e) Early time scale reaction trace zoomed into *t* = 0–300 s obtained by Wiesehan et al.^128^ showing first-order kinetics using *A*_inf_ (blue plot) and accompanying linear fitting (black dashes) for uncoupled (top) and strongly coupled systems (bottom). (f) Reaction summary showing reaction rate vs detuning where no enhancement was observed under a similar condition to that of (a–d). (a–d) Reproduced with permission from ref (126). Copyright 2019 Wiley. (e–f) Reproduced with permission from ref (128). Copyright 2021 American Institute of Physics.

VSC, often requiring sufficiently high concentrations of reactants or products, can also manifest when their vibrational mode frequencies overlap with those of solvents, allowing for lower concentrations in the “cooperative” strong-coupling regime. This concept was initially introduced in 2019, when the authors studied the hydrolysis of PNPA in ethyl acetate,^126^ where the premixed reactants and solvents were guided into the liquid flow cell simultaneously (Figure 22a). The overlapping CO modes of PNPA and solvents led to a notable 10-fold increase in the reaction rate constants, an effect not observed when the CO modes of the reactants were isotope labeled with ^13^C (Figure 22b–d). However, subsequent research^128^ by a different group could not reproduce the same enhancement, despite achieving similar strong-coupling conditions (Figure 22e–f). Nonetheless, analogous approaches have been extended to enzymatic reactions.^127,195^ For example, VSC was reported to decelerate peptide bond-cleavage reactions, where OH modes of pepsin and water—reactants and reaction media—attained the ultrastrong-coupling regime together, further enriching the impact of VSC-modified reactions.

In addition to homogeneous reactions, the concept of cooperative strong coupling has also been extended to heterogeneous reactions. By achieving strong coupling with the solvents of these reactions, it becomes possible to influence the self-assembly morphology and kinetics of conjugated polymers,^52^ as well as the crystallization process of MOF.^53^ However, the underlying mechanisms driving these modifications appear to be complicated and multifaceted.

#### 4.2.2 Other Thermally Activated Processes Under VSC Conditions

Currently, the influence of VSC has been extended beyond chemical reactions to encompass various solid-state phenomena. In one work, the phonon modes of a ferromagnetic material were strongly coupled to a cavity, resulting in a 700-fold enhancement of its ferromagnetic properties.^196^ In another study, the proton conductivity experienced a 10-fold increase when the OH modes of water were strongly coupled, a phenomenon that was further influenced by Rabi splitting and detuning.^54^ These pioneering efforts have illuminated the broader potential of using VSC to control physical process, shedding light on the phenomenological relationships between the degrees of freedom being strongly coupled and the consequent alterations in material behaviors. To advance this field, it is imperative to develop robust theoretical frameworks that can both explain and predict how VSC modulates these transport phenomena and the strongly correlated behaviors.

#### 4.2.3 Challenges and Opportunities

While the realm of VSC-enabled chemistry holds immense promise, it simultaneously presents puzzles that necessitate a concerted exploration between theoretical insights and experimental investigations. The paramount question revolves around the juxtaposition of the relatively limited DOS in polariton modes against the more abundant dark reservoir states. In contrast to the photoexcited reactions discussed in section 4.1, here the reactions are thermally activated. Consequently, considering the energy difference between polaritons and dark modes, often in the range of tens to hundreds of cm^–1^, coupled with the significantly higher DOS in dark reservoir modes, it is intuitively anticipated that any thermally activated reactions would predominately involve dark modes rather than polariton states. This argument impels the central debate of comprehending why and how the distinct attributes of polariton modes dominate the chemical reaction mechanisms. Resolving this question demands the development of theoretical frameworks capable of addressing VSC in the collective regime, alongside direct experimental exploration of the DOS of dark modes as well as the population transfer rate from dark and polariton modes.

Another enigmatic phenomenon lies in why and how the VSC-modified reactions depend on detuning, because many other modes coexist at *k*_||_ > 0 alongside with those at *k*_||_ = 0. The precise reasons underlying the selective influence on reaction kinetics only when the energy matches between the *k*_||_ = 0 cavity modes and the molecular modes, known as zero-detuning, remain a mystery.

It is also pivotal that the documented results can be reproduced across multiple laboratories. To facilitate this, we recommend that when disseminating novel observations of VSC-modified phenomena, comprehensive statistical analysis of the data, including but not limited to error bars, number of successful versus unsuccessful attempts, and criteria for identifying outliers, and the detailed data processing procedures, such as how reaction rates are calculated and whether they are normalized to factors such as reflectivity, cavity volumes, etc., should be provided. Equally critical are the reports of null results, accompanied by hypotheses regarding potential explanations and future tests. Lastly, employing a variety of standard analytical techniques to corroborate the results could be invaluable.

At its core, chemical reactions inherently occur on a local scale, such as breaking or forming a chemical bond. In contrast, the polariton modes exhibit delocalization. This implies that uncovering many of these intricacies hinges upon understanding how energy redistributes among the delocalized polaritons and localized dark modes. In this endeavor, ultrafast spectroscopy becomes an indispensable tool for shedding light on these multifaceted questions.

## 5 Ultrafast Dynamics of Molecular Polariton Systems via Coherent Multidimensional Spectroscopy

Considering the prevailing focus on using electronic and excitonic polaritons to modify photophysics and photochemical attributes, it has become intuitive to apply established ultrafast spectroscopic techniques on these systems. This approach enlightens the insights into how strong coupling of electronic transitions to photonic environments can reshape the ultrafast dynamics of electrons and/or excitons. Indeed, these techniques are widely applied to study ultrafast dynamics of strongly coupled inorganic polaritons.^62,197−202^ We will highlight one recent work as an example to establish key concepts for interpreting ultrafast polariton spectra. In their investigation, Vasa and co-workers^197,203^ studied surface plasmon polaritons formed by hybridization between a gold nanoslit array and the coating of a J-aggregated dye thin layer (cyanine dye 2,2-dimethyl-8-phenyl-5,6,5,6-dibenzothiacarbocyanine chloride). Upon photoexcitation, they observed a reduction in Rabi splitting, attributed to the bleaching of exciton absorptions. This phenomenon, termed “Rabi splitting contraction”, manifested as a derivative feature in the pump probe spectra, accentuating the differences between spectra with and without optical pump pulses. Thus, the Rabi splitting contraction is ubiquitous in polaritons and should be considered as a baseline before postulating novel phenomena. Yet, in some systems,^204^ transient heating of the cavity optics could potentially confound results, warranting a thorough exclusion of this artifact. Alternatively, cavity optics can be designed such that the absorptivity and nonlinearity of the cavity materials is much lower than the molecular systems, to mitigate or avoid transient heating and other parasite nonlinear effects. For example, in the ultrafast dynamics of vibrational polaritons at 5 μm discussed below, there were not any nonlinear signals (including transient heating) from the cavity optics. Musser and co-workers have discussed potential artifacts arising from changes in refractive indices and transient heating in ref (204). We refer interested readers to this reference for more detailed information.

Moving forward, we delve into the realm of ultrafast studies on molecular polaritons, mostly using coherent multidimensional spectroscopy, such as two-dimensional infrared (2D IR) and two-dimensional electronic (2D E) spectroscopy, organized chronologically. We put an emphasis on this technique because it is important to separately measure the dynamics involving UP, LP and dark modes—a strength of 2D spectroscopy. While the first ultrafast studies on molecular polaritons were conducted on electronic polariton systems,^131^ a systematic exploration of ultrafast molecular vibrational polariton dynamics was catalyzed by the seminal work from Naval Research Laboratory in 2016,^60^ followed by a series of investigations implementing 2D IR spectroscopy on vibrational polaritons.^4,59,67^ Thus, we will first discuss molecular vibrational polaritons and then navigate toward the burgeoning realm of 2D E spectroscopy of molecular electronic/exciton polaritons which have garnered their own momentum recently.

### 5.1 Ultrafast Dynamics of Vibrational Polariton Systems via 2D IR Spectroscopy

#### 5.1.1 Nonlinear Ultrafast Spectroscopy Due to Polariton-Dark Mode Incoherent Interactions

**Figure 23:**
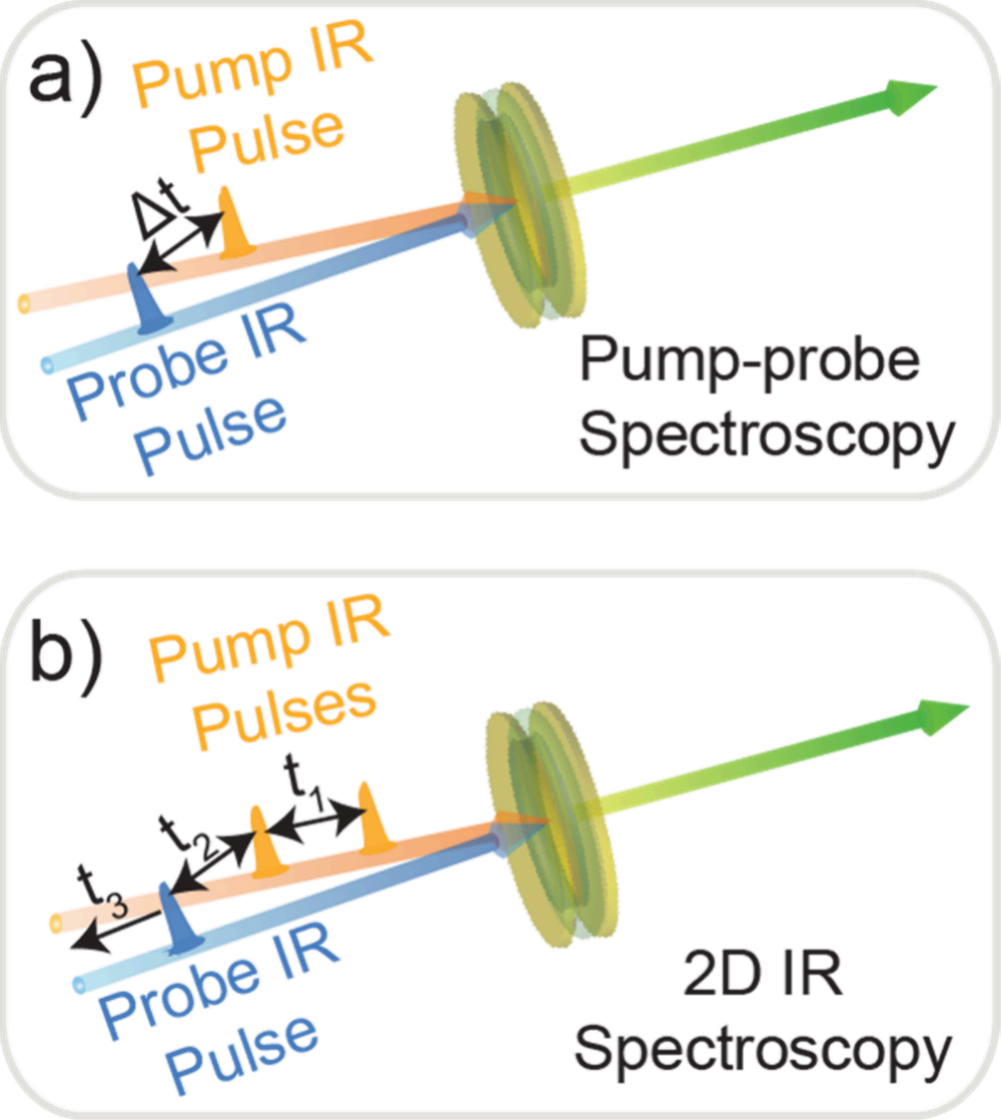
Schematic illustration of ultrafast measurements. (a) Pump–probe with a time delay (Δt) between pump and probe pulses, and (b) two-dimensional infrared (2D IR) spectroscopy, where t_1_ is the time delay between two pump pulses that characterize the initial coherence, t_2_ is the time delay between the second pump and probe pulses that could characterize either a coherence or population dynamic, and t_3_ is the free induction decay time following the probe pulse interacting with the sample. The Fourier transform of t_1_ (numerical) and t_3_ (instrumental) will generate the two frequency axes (ω_1_, excitation frequency axis; and ω_3_, detection frequency axis) in the 2D IR spectrum, while the scanning of t_2_ gives rise to the dynamic features of the system.

**Figure 24:**
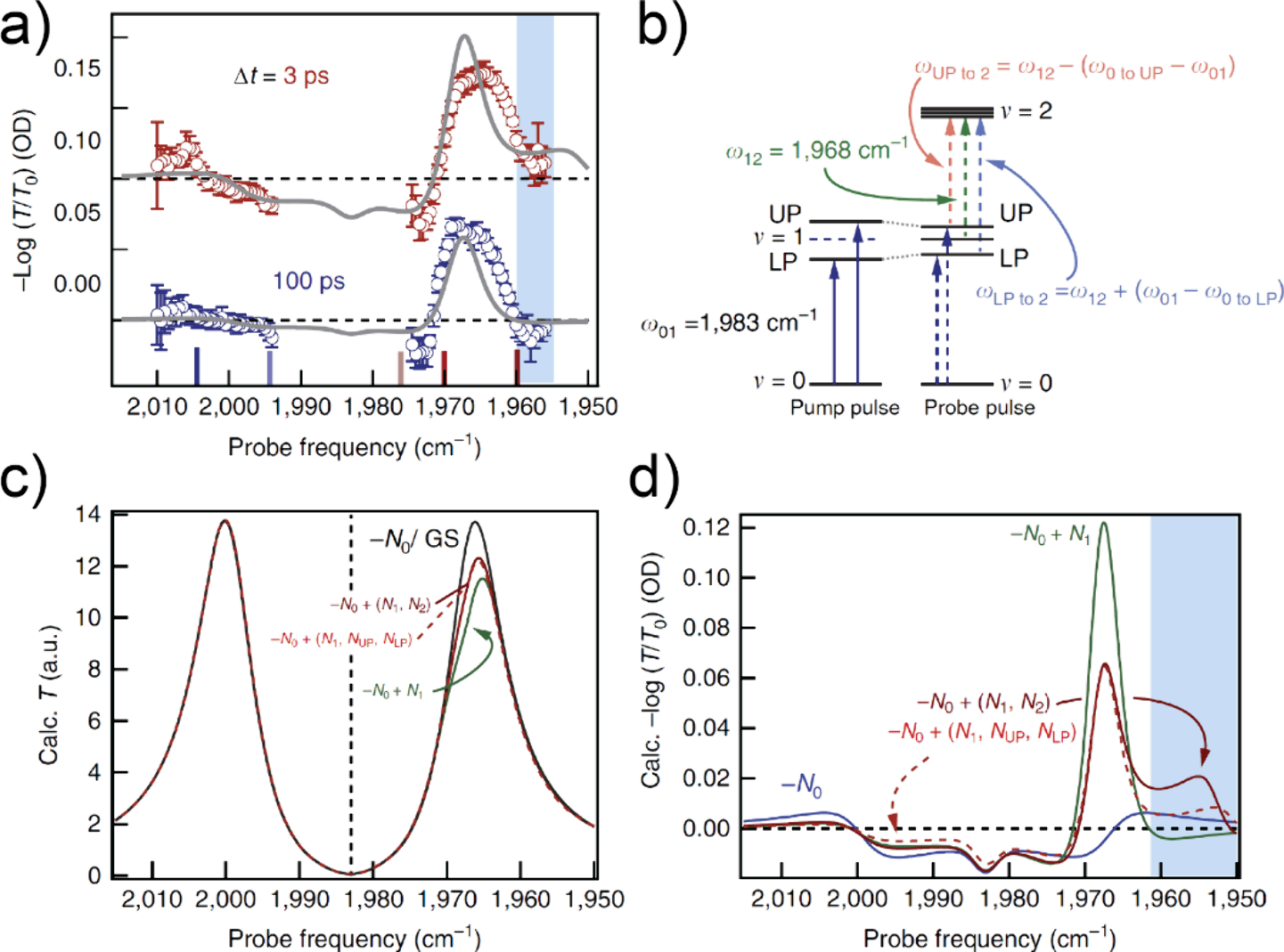
Ultrafast pump probe dynamics of molecular vibrational polaritons. (a) Transient spectra of cavity-coupled W(CO)_6_ (20 mM in hexane) measured 3 (red) and 100 ps (blue) after excitation. Traces are offset for clarity. The blue shaded area highlights the low-frequency response that indicates UP population. (b) Schematic representation of energy levels and transitions involved in the cavity-coupled transient absorption experiment. (c) Calculated transmission spectra for varying amplitude (population) of oscillators at the frequencies described in (b). (d) Calculated differential absorption spectra for the same population distributions used in (c). The blue shaded area highlighted the low-frequency response that preliminarily was assigned to UP population. Reprinted with permission from ref (60). Copyright 2016 Nature Publishing Group.

Dunkelberger, Owrutsky, Simpkins and co-workers used IR pump–probe spectroscopy to study a molecular vibrational polariton first.^60^ They prepared the system by encapsulating a solution of W(CO)_6_ in F–P cavities, composed by two DBR optics. The cavity modes were tuned to strongly couple to the asymmetric stretch of CO in W(CO)_6_, denoted as the T_1u_ mode. Employing a broadband ultrashort IR pulse near 5 μm, which resonantly pumps all states in the cavity systems, they initiated the nonequilibrium polariton dynamics. Subsequently, they probed the spectral changes using another femtosecond IR pulse (Figure 23a). At long t_2_, akin to the exciton polariton scenarios, a derivative spectral signature appeared in proximity to ω_probe_ = ω_UP_. Intriguingly, when ω_probe_ = ω_LP_, a substantial absorptive feature emerged (Figure 24a). The authors attributed the derivative feature to the Rabi splitting contraction (Figure 24b) and pointed out that a distinguishing aspect from exciton polariton systems was that molecular vibrations strongly coupled to the cavity modes were not two-level systems. Rather, transitions from υ = 1 to 2 overlapped with the LP transition due to anharmonicity, contributing to the pronounced absorptive peak near ω_probe_ = ω_LP_. These theoretical explanations were supported through numerical simulations employing TMM models (Figure 24c–d). The authors also observed secondary subtle features initially designated as transitions to double excitation of polaritons, but the assignments were revised later through subsequent investigations.^113,122^ This work is particularly groundbreaking as it is the first of its kind, and building on top of the Rabi splitting contraction mechanisms, the authors pointed out that anharmonicity could contribute to the polariton transient absorption spectra, which lays the foundation of future ultrafast polariton spectra interpretation that is intrinsically different from its counterparts of molecular species.

**Figure 25:**
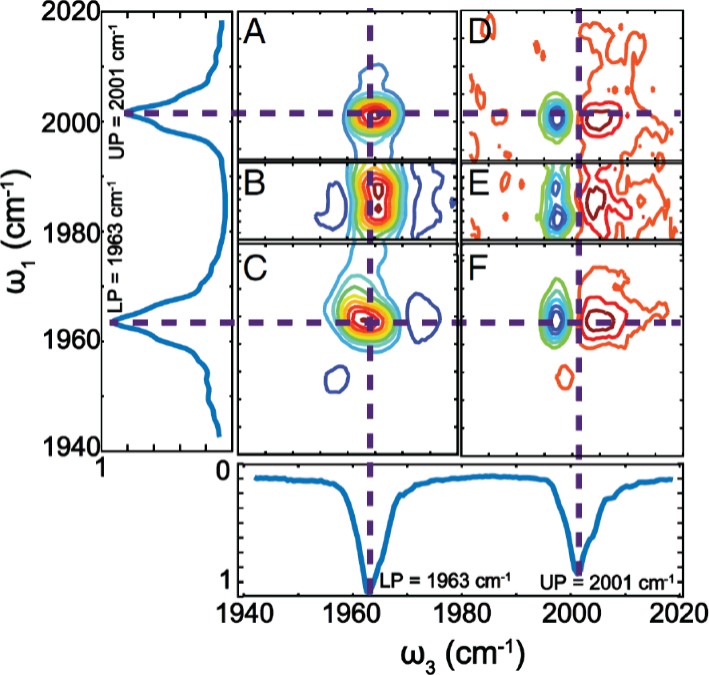
Two-dimensional IR spectrum of W(CO)_6_/cavity–polariton system at 25 ps delay with −2 cm^–1^ detuning. Each spectral region is scaled to its own intensity maximum and minimum. Spectra of the pump (ω_1_) and probe (ω_3_) pulses are shown on their respective axes. Color map: red is positive, blue is negative. Reprinted with permission from ref (59). Copyright 2018 National Academy of Sciences.

Inspired by these groundbreaking efforts, Xiang, Xiong and their colleagues at UCSD along with the NRL team conducted the first 2D IR spectroscopy (Figure 23b) of the same polariton systems.^59^ At long t_2_, the 2D IR spectra (Figure 25) resembled the pump–probe spectra along the ω_probe_ axis. The distinction lays in the ω_pump_ axis, where the excitation states were discernibly resolved. This allowed the distinct appearance of the UP and LP resonances at their respective characteristic frequencies along the ω_pump_ axis, facilitating tracking of polariton dynamics individually. Notably, the cross-peak features arising from pumping polaritons and probing the υ = 1 to 2 transitions through the LP window provided an informative signature for monitoring population transfer from polaritons to υ = 1 excited states of dark modes. The resolution against excitation frequency proved valuable, particular as the 2D IR spectra revealed modest yet non-negligible peaks at ω_pump_ = ω_dark_, hinting at the optical characters of dark modes, a divergence from the ideal case. This deviation had been predicted by theory,^97^ where disorder could disrupt the symmetry of dark modes, bringing them optical brightness. Realistically, the dark mode features new avenues for tracking their dynamics, permitting insightful comparisons with polariton states. Later, a theoretical model of 2D IR of polaritons was introduced by Ribeiro, Yuen-Zhou and co-workers,^99^ further corroborating the assignment of Rabi splitting contraction and accentuating the significance of considering both mechanical and electronic anharmonicity in the molecular vibrational polariton systems.

Recognizing the importance of distinguishing optical responses from dark and polariton modes, Grafton and co-workers at NRL devised a subtraction scheme.^122^ They measured 2D IR at long t_2_ and then calculated the difference between the spectral cuts at ω_1_ = ω_LP/UP_ and ω_1_ = ω_dark_. The underpinning assumption was that the excitation of dark modes would yield the same nonlinear signals across any of these spectral cuts (albeit different amplitudes). By subtracting this contribution from the spectral cuts at ω_1_ = ω_LP/UP_, the remaining signals were inferred to be exclusively from polaritons. Notably, they uncovered long lasting signals that were attributed to incoherent polariton nonlinear responses. The authors further assigned these peaks based on a quantum mechanical model encompassing a small number of molecules within strong-coupling regimes. While these treatments and assignments are thought-provoking, caution is advised as the nonlinear optical signal might not be directly subtractable, as pointed out by the same authors later.^98,205^ Furthermore, in cases where the chemical systems are inhomogeneously broadened, the spectral heterogeneity and spectral diffusion could also contribute to the observed 2D IR polariton features.^98,205,206^

#### 5.1.2 Vibrational Dynamics of Polaritons Composed of a Single Vibrational Mode

**Figure 26:**
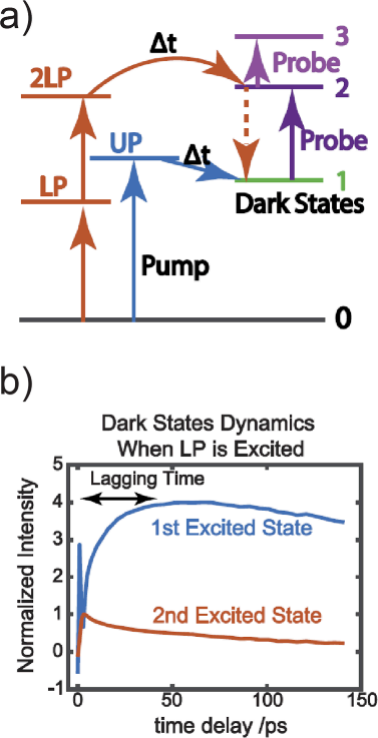
Ultrafast dynamics of molecular polaritons under selective pumping using 2D IR spectroscopy. (a) Dynamics of strongly coupled W(CO)_6_ when LP is excited show a delayed relaxation of the first excited state of the dark mode and a corresponding large second excited-state population. (b) LP first relaxes to second excited states through polariton–polariton scattering before relaxing to first excited dark modes. Reprinted with permission from ref (67). Copyright 2021 American Institute of Physics.

Building upon the spectral interpretations established in these works, Xiang and his colleagues conducted further analysis on the time–dependent dynamics of 2D IR spectra of vibrational polaritons, comprised of the same W(CO)_6_ in an F–P cavity system. They revealed that the excited LP states could selectively pump the dark modes to their second excited states. This phenomenon was attributed to the resonance between 2LP and ω_02_ transitions^67,113^ (Figure 26)—because the doubling of LP transition frequency matches with the frequency of the *v* = 0 → 2 transition, it enhances the excitation of the second excited states of dark modes when the LP scatters with each other. This insight was also supported by both analytical^207^ and numerical simulation^208^ investigations. Additionally, they observed that this transition was influenced by the solvent environment, with a preference for nonpolar solvents. Recently, a follow-up study from the same group found additional evidence to propose the involvement of Raman active E_g_ modes to assist the LP relaxation to the dark modes, remaining to be further verified by a direct IR pump and Raman probe experiment.^209^ The authors reasoned that the larger energy gap between the LP and E_g_ modes makes a solvent-assisted Raman transition become more favorable than the one outside of the cavity. These results indicate that under VSC, new eigenstates are prepared, thereby amplifying nonlinear processes that would otherwise remain insignificant in the absence of cavities.

#### 5.1.3 Vibrational Energy Transfer and Isomerization Dynamics in Polariton Systems

The distinctive capability of 2D IR spectroscopy to monitor energy transfer between different polariton modes and dark reservoir states enables new insights into how these polariton states influence vibrational energy distribution within and among molecules. Notably, two significant examples illustrate this phenomenon: (1) polaritons facilitate vibrational energy transfer among distinct molecules;^103^ (2) they expedite intramolecular energy redistribution while simultaneously slowing down competing dynamics. Importantly, the same effect was not observed on the dark modes.^104^

**Figure 27:**
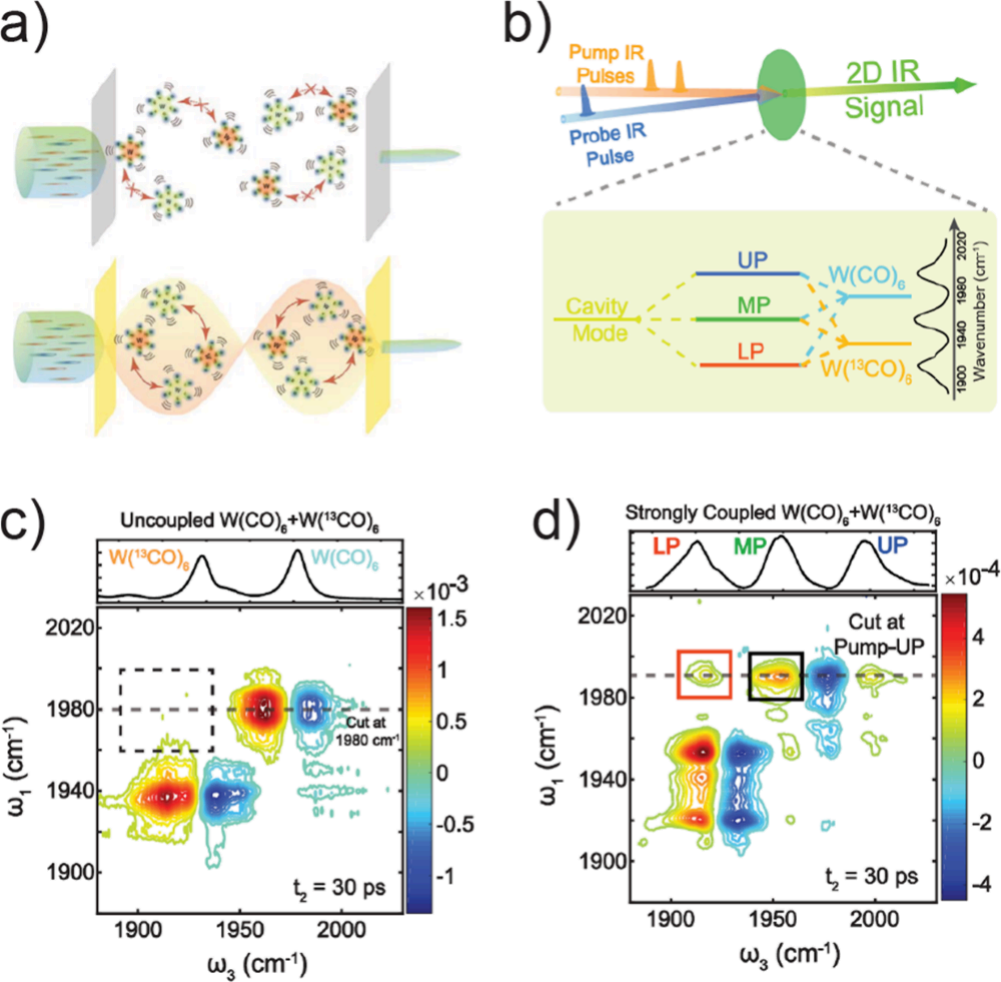
VSC-enabled ultrafast intermolecular vibrational energy transfer. (a) Schematic illustration showing that VET between vibrational modes of W(CO)_6_ and W(^13^CO)_6_ molecules is unfavorable in solution phase (top) but is enabled by strong coupling of the molecular system to an infrared cavity mode (bottom). (b) Diagram of the 2D IR pulse sequence along with the IR spectrum and energy diagram of the system. 2D IR spectra of (c) uncoupled and (d) strongly coupled W(CO)_6_/W(^13^CO)_6_ with a total concentration of 105 mM in binary solvent (hexane/DCM), along with the corresponding linear spectra of the two systems (top panels). Reprinted with permission from ref (103). Copyright 2020 American Association for the Advancement of Science.

Xiang and colleagues engineered a three-polariton system by strongly coupling the asymmetric modes of W(CO)_6_ and W(^13^CO)_6_ to an F–P cavity mode (Figure 27a and b). In scenarios where the molecules were outside of the cavity, no energy transfer occurred between them (Figure 27c). Interestingly, under VSC condition, a large cross-peak emerged at the [ω_UP_, ω_LP_] position, signifying that by resonantly exciting the UP state, a substantial population was directed toward the dark modes of LP—the asymmetric mode of W(^13^CO)_6_ (Figure 27d). Through comprehensive quantitative analysis, it was shown that when exciting the UP state, the relative excited population between the W(CO)_6_ and W(^13^CO)_6_ was about 2.5:1. Remarkably, this ratio deviated from the 14:1 ratio defined by the Hopfield coefficient, indicating a higher population of the W(^13^CO)_6_ excited states. This observation supported the notion of an energy transfer channel that facilitated the extra excitation in W(^13^CO)_6_. Additional vibrational dynamics substantiated the existence of the new energy transfer channel, as indicated by the swifter emergence of the W(CO)_6_ excited state through direct relaxation from UP, versus the relatively delayed appearance of the W(^13^CO)_6_ excited state via the energy transfer channel. Moreover, a recent study by Cao and co-workers^210^ introduced a generalized resonant energy transfer scheme, revealing that the delocalization of polaritons can expedite the energy transfer rates.

**Figure 28:**
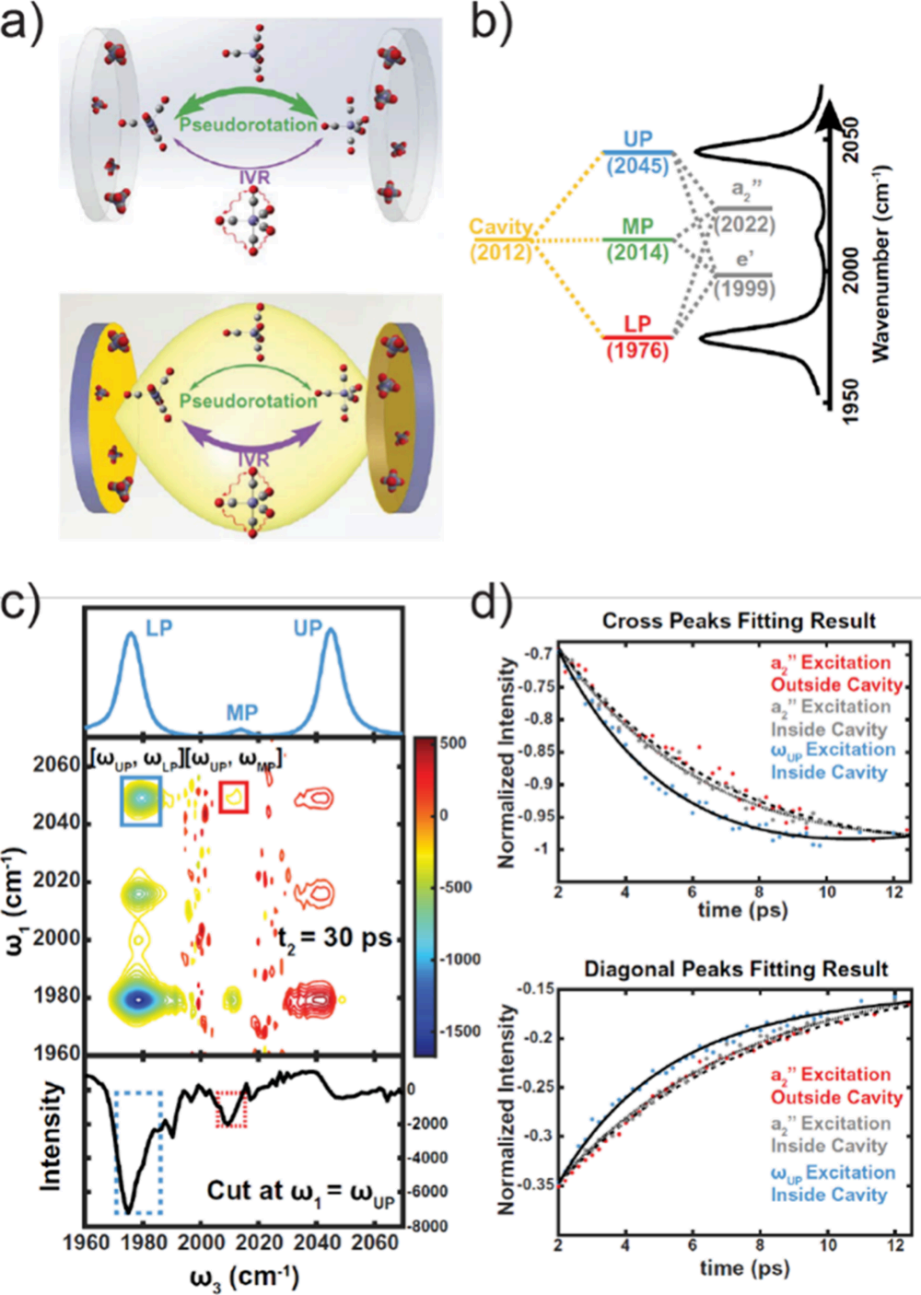
VSC-modified ultrafast molecular dynamics: decelerating pseudorotation and speeding up IVR. (a) Schematic drawing showing that when Fe(CO)_5_ is outside of the cavity, pseudorotation is the dominating channel (top). when the molecule is placed in an optical cavity, IVR becomes the dominant energy-exchange process and pseudorotation is suppressed (bottom). (b) Strong-coupling diagram and IR spectrum of Fe(CO)_5_ inside the cavity. (c) Normalized 2D IR spectrum using the linear spectrum of strongly coupled Fe(CO)_5_ at waiting time (t_2_) = 30 ps in dodecane (blue and red boxes represent [ω_UP_, ω_LP_] and [ω_UP_, ω_MP_] peaks, respectively), along with the corresponding linear spectrum (top) and normalized narrowband pump probe spectrum at ω_1_ = ω_UP_ (bottom). (d) Experimental dynamics of cross-peaks (top) and diagonal peaks (bottom) for Fe(CO)_5_ outside the cavity upon pumping of the a_2_″ modes (red dots) and inside the cavity upon pumping of the UP (blue dots) and the a_2_″ dark modes (gray dots). The black dashed, dotted, and solid lines are the corresponding fits, respectively. Energy is exchanged at a faster rate when pumping the UP, whereas pumping the a_2_″ dark modes leads to a rate similar to that outside the cavity. Reprinted with permission from ref (104). Copyright 2022 American Association for the Advancement of Science.

A notable advancement in comprehending the impact of polaritons on molecular reaction dynamics was recently achieved by Chen, Du, Yang and co-workers.^104^ The authors targeted a well-studied ultrafast isomerization process that was a single barrier process of Fe(CO)_5_ in solution, recognized as Berry’s pseudorotation^211,212^ (Figure 28a and b). Given its resemblance to elementary reactions due to its single barrier transition, this process served as an ideal model. The pseudorotation involved the exchange of two of the CO ligands, axial (A_2g_) and equatorial (E_g_) modes, resulting in an apparent 90° rotation of the molecules, though actual rotation was absent, and only ligand rearrangement transpired. By strongly coupling both A_2g_ and E_g_ modes with the F–P cavity modes, the researchers observed an accelerated overall energy exchange compared to the scenarios of outside of cavity.

However, the enhanced energy exchange could be attributed to either pseudorotation or intramolecular energy redistribution (IVR).^213−215^ To elucidate these two cases, anisotropy dynamics were measured, considering that pseudorotation entails energy exchange between two parallel dipoles, whereas IVR involves two modes perpendicular to each other. The anisotropy results suggested the coexistence of pseudorotation and IVR under both VSC and out-of-cavity conditions. Surprisingly, VSC expedited IVR and decelerated pseudorotation dynamics, altering the energy exchange dynamics from pseudorotation-dominant to IVR-dominant, which manifested as a change of initial anisotropy sign. Notably, only polaritons can modify the molecular energy exchange dynamics, as exciting dark modes failed to evoke changes (Figure 28c and d). Similar insights were garnered from QM-MM cavity simulations by Li and Hammes-Schiffer, confirming the role of polaritons and dark modes in reshaping energy transfer and molecular dynamics.^216^

In a recent breakthrough, the Chuntonov group^217^ introduced a novel approach to achieve strong coupling of vibrational modes of (poly)methyl methacrylate (PMMA) with surface lattice resonance (SLR). Unlike F–P cavities, SLR forms an open cavity structure, enabling simultaneous resonant excitations of polaritons and dark modes. This configuration offers the advantage of reaching vibrational strong coupling with a smaller number of molecules within the cavity volume compared to the F–P cavities. A notable feature is the capacity for probing energy transfer between dark modes and polaritons. Intriguingly, they identified a cross-peak at [ω_dark_, ω_LP_]. Instead of attributing this peak to the dark 1–2 transition, the authors assigned it as a direct excitation of the LP population. The assignment was determined by the fact that the 1–2 transition was relatively red-shifted compared to the LP peak frequency. Notably, the spectral position could also involve contributions from transitions of higher-level vibrational modes. Based on their assignments, the authors derived a rapid population transfer time of ∼200 fs from the dark to the LP state. This fast transfer rate challenged the convention argument of the entropy penalty (i.e., *k*_D→LP_ = 1/*N* × *k*_LP→D_) underscored by the low DOS of LP. Nevertheless, this outcome aligned with the polariton transport experiments^12,14,218^ and certain spin dynamics,^49,51^ offering intriguing implications for revisiting the DOS arguments.

#### 5.1.4 Applications in IR Photonics

Approaching the concept from a different perspective, Dunkelberger and co-workers focused on exploring ultrafast Rabi splitting contraction for ultrafast all-optical switches. At the intermediate coupling regime, they revealed that up to 40% of molecules could be excited, in the W(CO)_6_ polariton system, while the excited-state dynamics remain similar to those observed outside of the cavity.^123^ Subsequently, the researchers harnessed a single pulse to saturate the vibrational mode of W(CO)_6_, which relaxed in hundreds of ps. This research laid the groundwork for a promising avenue toward ultrafast optical switch capabilities.^121^

### 5.2 Ultrafast Dynamics of Electronic and Excitonic Polariton Systems via 2D Electronic Spectroscopy

Within the realm of ultrafast dynamic studies of ESC systems, a coherent consensus regarding the interpretation of results remains to be fully established. Pioneering efforts by Vasa and co-workers^197^ have showcased the occurrence of Rabi splitting contraction upon photoexcitation, as discussed above. This contraction gives rise to derivative-like lineshapes in both pump–probe and multidimensional spectroscopy.

Rury and co-workers undertook visible pump–probe spectroscopy to delve into energy relaxation processes of metalloporphyrin-based polariton systems.^219,220^ They observed altered internal conversion dynamics contingent upon the magnitude of the Rabi splitting. Furthermore, they proposed the emergence of a new nonradiative channel to the ground states as a consequence of polariton formation. This new pathway ran in parallel to the pre-existing relaxation route of molecules outside of cavities.

Several studies have employed 2D E spectroscopy to investigate exciton polariton systems and have asserted their ability to follow polariton dynamics, such as polariton relaxation and the transfer rate from dark mode to polaritons. However, while a couple of works consider the substantial impact of the Rabi splitting contraction to the 2D Espectra (as discussed in section 5.2.1), a few works^63,65^ concluded other novel effects while omitting this ubiquitous effect in VSC and ESC systems.

#### 5.2.1 Remote Energy Transfer between Strongly Coupled Carbon Nanotubes

**Figure 29:**
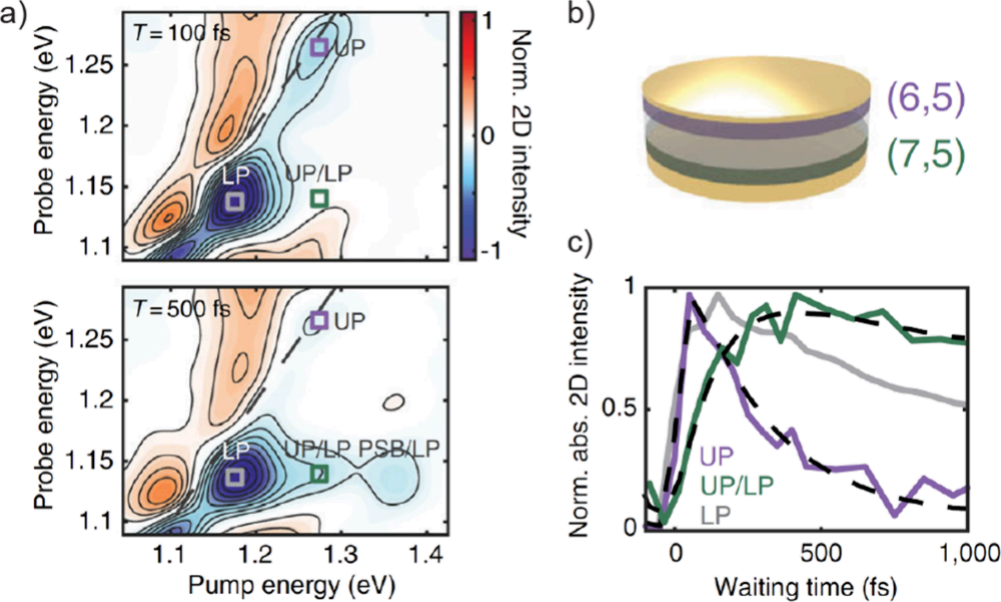
Evidence of ultrafast excitonic energy transfer via exciton polaritons (a) 2D white light spectra of the microcavities with (6,5)/(7,5) CNTs at waiting times (T) of 100 fs (top) and 500 fs (bottom). (b) Cartoon illustrations of the mixed (6,5)/(7,5) microcavities. (c) Normalized waiting time traces (colored solid lines) generated at the peak positions labeled with open squares in (a). Reprinted with permission from ref (221). Copyright 2022 Nature Publishing Group.

A recent 2D E spectroscopy work has reported a long-distance energy transfer mediated by polaritons in carbon nanotube systems. In this work,^64,221^ Son, Arnold, Zanni and co-workers prepared a three-polariton system by interleaving layers of (6,5) and (7,5) carbon nanotubes between cavity optics, with a 150 nm polymer spacer separating the carbon nanotube layers (Figure 29b). Their findings showed unambiguously a growing cross-peak between UP and LP, indicating downhill energy transfer from (6,5) to (7,5) nanotubes (Figure 29a). The researchers further explained their observations using Redfield theory. They attributed the energy transfer dynamics (Figure 29c) to interactions between polaritons and a manifold of dark modes that possess a degree of delocalization and optical brightness owing to disorder.

#### 5.2.2 Reduced Thermal Bath Fluctuation Due to Polaron Decoupling

Another unique insight provided by 2D E spectroscopy is the system–bath coupling. It has been proposed that polariton formation can enable a mechanism known as polaron decoupling.^222^ In essence, the rapid transitions of matters between their ground and exited electronic potential energy surfaces left little time for coupling to the low frequency modes, such as phonon or thermal fluctuations. However, experimental evidence of this phenomenon has been missing until the 2D E spectroscopy work by Takahashi and Watanabe.^223^ In their study, the authors created exciton polaritons using the exciton transition near 2 eV in tetraphenyldibenzoperiflanthene (DBP) within a metal-coated F–P cavity. Subsequently, they quantified the nodal-line slope (NLS) of the 2D E spectra of polaritons. The NLS measures the frequency–frequency correlation function^224^ of the systems at a waiting time (t_2_), where a value of 1 indicates complete correlation, while a value of 0 suggests a complete lack of correlation. Intriguingly, in their experiment, NLS manifested a zero slope under a large strong-coupling condition, in sharp contrast to the slope of 0.5 observed in systems without strong coupling. This result indicated that, while DBP films exhibited inhomogeneous broadening and spectral diffusion indicative of coupling to bath fluctuations, under strong-coupling conditions, the system–bath coupling weakens, thereby supporting the concept of polaron decoupling. Notably, the authors used the traditional 2D spectroscopic features, i.e., ground-state bleach/stimulated emission and excited-state absorption, to describe the 2D E spectra of exciton polaritons, without considering the Rabi splitting contraction. However, the NLS analysis remains robust, as the frequency–frequency correlation persists even when using the Rabi splitting contraction to interpret the 2D E spectral data.

#### 5.2.3 Alternative Theoretical Model

A recent work has proposed an intriguing alternative explanation of the visible transient absorption, which could also be extended to 2D E spectroscopy.^118^ Rather than attributing the derivative feature to Rabi splitting contraction, the authors put forth a quantum-based description. In this view, the 2D E spectra were described to probe the energy difference between the first and second rungs of polariton levels, stemming from anharmonicity. While this quantum description aligns well at single-particle strong-coupling regime^9^ at the collective coupling regime, the polariton potential energies are harmonic. This leads to equal energy spacings between the first and second rungs of polariton states. The evidence for the quantum interpretation hinges on the fact that the transient spectral features only changed intensities rather than peak position with the increase of pump power. However, this observation can also be well explained by Rabi splitting contraction when considering that the spectroscopic studies were conducted within a perturbative regime.^67^

### 5.3 Challenges and Opportunities

While the ultrafast dynamics of polaritons offer a unique window into energy dynamics in molecular polariton systems, a few challenges persist. First, like in any cases when new technologies are applied toward understanding new phenomena, there are diverse explanations to the experimental observables, sometimes leading to difficulties in interpreting experimental data. To resolve it, it is necessary for the community to form a unified theoretical picture that can comprehensively describe the ultrafast spectral features of polaritons, so that the community can build upon this theory, further developing and using it to describe ultrafast dynamics of polaritons. This entails determining whether a semiclassical model, namely, Rabi splitting contraction and excited-state absorption of dark modes, is sufficient to account for the spectral features, or if a quantum description is needed. This aspect is particularly critical for measuring population transfer rates between polariton states and between polaritons and dark modes.

Second, while significant progress has been made in understanding chemical processes involving single-barrier crossing events, the influence of photoexcited polaritons on more complicated chemical process, such as elementary and complex reactions, remains to be thoroughly examined.

Lastly, in ultrafast experiments, specific polaritonic modes are photoexcited. Bridging the gap between the photoexcited measurements and thermally activated polariton-modified process is essential for establishing a robust theoretical foundation for polariton chemistry. In terms of exploration, an intriguing question yet to be fully answered is how polaritons with electronic degrees of freedom influence the dynamics of vibrational degrees of freedom, and vice versa, thus expanding the scope of ultrafast polariton photochemistry.

## 6 Molecular Coherence in Strong-Coupling Regime

**Figure 30:**
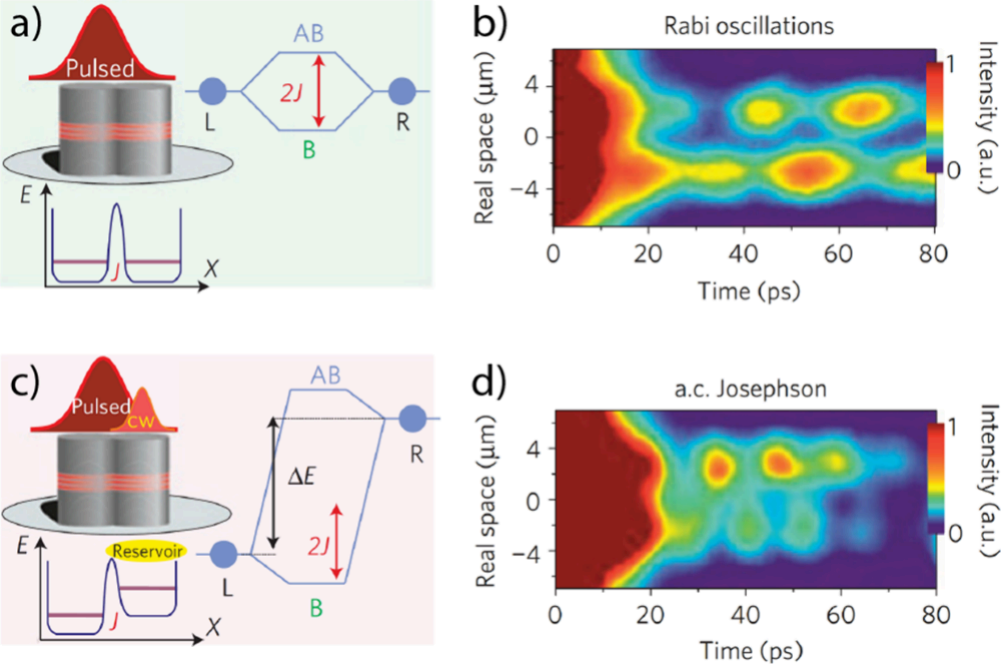
Rabi oscillations and AC Josephson effect in exciton polariton condensates. (a) Polaritonic molecule. The coupling J (0.1 meV) between the lowest energy state (ground state) of each micropillar (L, R) gives rise to bonding (B) and antibonding (AB) modes. (b) Emitted intensity when an off-centered Gaussian pulse at low power (2.5 mW) excites the system. (c) The AC Josephson regime is achieved by adding a CW beam on top of the right micropillar, which creates a reservoir (shown in yellow) inducing a static blue-shift of its ground-state energy. (d) The larger bonding–antibonding splitting results in faster intensity oscillations. Reprinted with permission from ref (167). Copyright 2013 Nature Publishing Group.

Quantum phenomena in condensed-phase molecular systems have been extensively explored and simulated within solid state^225^ or Bose–Einstein condensates^226^ while circumventing the requirement of cryogenic temperature. A few important criteria of the quantum technology platform include long-lived coherence, well-defined initial states, nonlinear interactions and scalability. In exciton polariton systems, coherence is achievable in coupled quantum states.^167,227−230^ Abbarchi et al.,^167^ for example, created quantum coherences by combining two coupled polariton condensates in two overlapping Al_0.95_Ga_0.01_As/Al_0.20_Ga_0.80_As λ/4-layered micropillar cavities that strongly coupled to the exciton mode in GaAs quantum wells (Figure 30a). These coupled polariton condensates exhibited correlated Rabi oscillations (Figure 30b) with a phase difference of π and an oscillation frequency matching with the Rabi splitting. They further exerted a CW excitation on one of the micropillar cavities to generate a blue-shifted reservoir mode that could interact with ground-state polariton condensate in the other micropillar cavity, reaching the AC Josephson regime—a coherence, which serves as the alternating current (AC), flowing back and forth through the junction created between the left and right pillars with a barrier. (Figure 30c). Tuning the energetic parameters enabled the acceleration of the oscillation (Figure 30d) due to a larger energy gap between the condensate and reservoir. In such exciton polariton systems, the strong interactions between excitons and cavity photons and ordered orientation in solid-state samples offer advantages to create and manipulate long-distance quantum coherences.

Unlike the exciton systems in condensed matter, molecular excitations often exhibit weak dipole–dipole interaction^83^ and are randomly distributed in their environments. The emergence of molecular polaritons amalgamates the large nonlinearity and structural tunability inherent to molecular modes, with the delocalized and reconfigurable characteristics of cavity modes. Consequentially, they inherit a delocalized nonlinearity, surpassing that of their parental molecular states. Furthermore, the distinctive attributes of cavities interplay synergistically with molecular polaritons, rendering these systems highly amenable to design and engineering. Importantly, the initialization of molecular polariton systems can be reliably executed by external laser fields. This section summarizes the ongoing endeavors directed toward harnessing the strongly coupled molecular systems as platforms for quantum simulation or information processing encompassing energy, temporal and spatial tunability.

### 6.1 Establishing Quantum States under Vibrational Strong Coupling

To delve into the vibrational coherence in the strong-coupling regime, Xiang, Ribeiro and co-workers embarked on an exploration of the early time dynamics of vibrational polaritons. These polaritons were prepared by encapsulating a nearly saturated solution of W(CO)_6_ in hexane within a Fabry–Pérot microcavity.^4^ The newly established UP and LP modes effectively merge the delocalization characteristic of photon cavity modes with the intrinsic nonlinearity of molecular modes.

**Figure 31:**
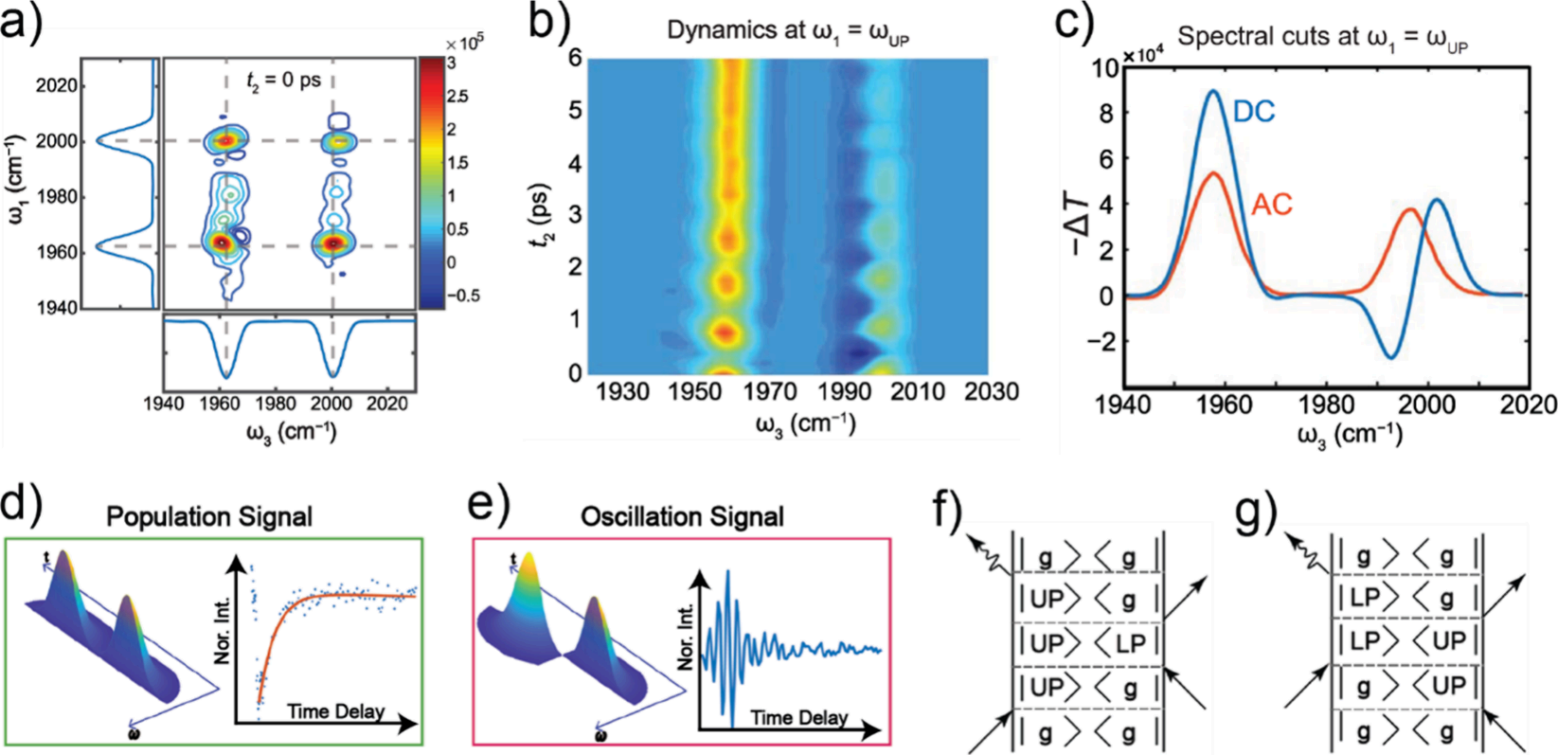
Polariton coherence in strongly coupled near-saturated W(CO)_6_/hexane solution. (a) 2D IR spectrum at t_2_ = 0 ps. (b) 2D IR dynamics at pump frequency ω_1_ = ω_UP_. (c) Spectral cuts for oscillating (AC) and nonoscillating (DC) components disentangled by Fourier transform along the t_2_ axis. Pump pulse sequences of 2D IR signal of (d) polariton population and (e) polariton coherent oscillation. Representative Feynman diagrams showing the creation of (f) |UP><LP| and (g) |LP><UP| coherences, shown in double-sided Feynman diagrams with 2D IR pulse sequence. (a–c), (f–g) Reprinted with permission from ref (4). Copyright 2019 American Association for the Advancement of Science. (d–e) Reprinted with permission from ref (68). Copyright 2020 American Chemical Society.

Xiang and colleagues found that at early time,^4^ before polariton decaying into the dark modes, the polariton–polariton nonlinear interaction led to an effectively accelerated dephasing phenomenon.^1^ This phenomenon induced a transient spectrum with absorptive lineshapes, referred to as polariton bleach, for both peaks at ω_LP_ and ω_UP_. Figure 31a shows the early time absorptive 2D IR feature dominated by the coherent polariton nonlinear interactions. The diagonal and antidiagonal absorptive peaks showcased the strong correlation between UP and LP states. The accelerated dephasing mirrored analogous observations reported in plasmonic excitations—excitations of collective charge motions.^1^ While drawing parallels, it is noteworthy that a meticulous theoretical model to describe the nonlinear interaction that originated from polariton dephasing is still lacking for molecular vibrational polaritons and could offer invaluable insights, not only for characterizing vibration polariton interactions but also for developing new quantum systems.

The dynamics revealed by 2D IR spectra (Figure 31b) at pump frequency ω_1_ = ω_UP_ further elucidated the coherent oscillation between UP and LP states. This oscillation stems from the energy exchange between cavity electromagnetic field and the collective molecular vibrations, and it exhibited a period of ∼0.8 ps, indicating an oscillation frequency of 40 cm^–1^ that corresponds to the Rabi splitting frequency. The Fourier transform along the t_2_ axis further disentangled the distinct signatures of polariton coherence (AC spectrum, red in Figure 31c) and population transfer from polariton to dark modes^59,99,113^ (DC spectrum, blue in Figure 31c). These two parallel dynamics came into play immediately following the excitation of polaritons. A subsequent work by Yang and co-workers employed tailored pump pulses in 2D IR experiments where the spectral features of the two pump IR pulses could be tuned conveniently using a pulse shaper, directly disentangling the signals corresponding to polariton population and coherence dynamics (Figure 31d and e).^68^ These disentangled polariton coherence responses showcased the ability in establishing arbitrary coherence |UP><LP| (Figure 31d) and |LP><UP| (Figure 31e) through a three-pulse 2D IR pulse sequence, thereby paying the way for their utility as quantum bits (qubits). In this molecular vibrational polariton system, the polariton coherence embodied the delocalization property from the cavity photons. This, in turn, served as the cornerstone for the propagation of long-distance vibrational coherences.

### 6.2 Spatial Propagation of Polariton Coherence

**Figure 32:**
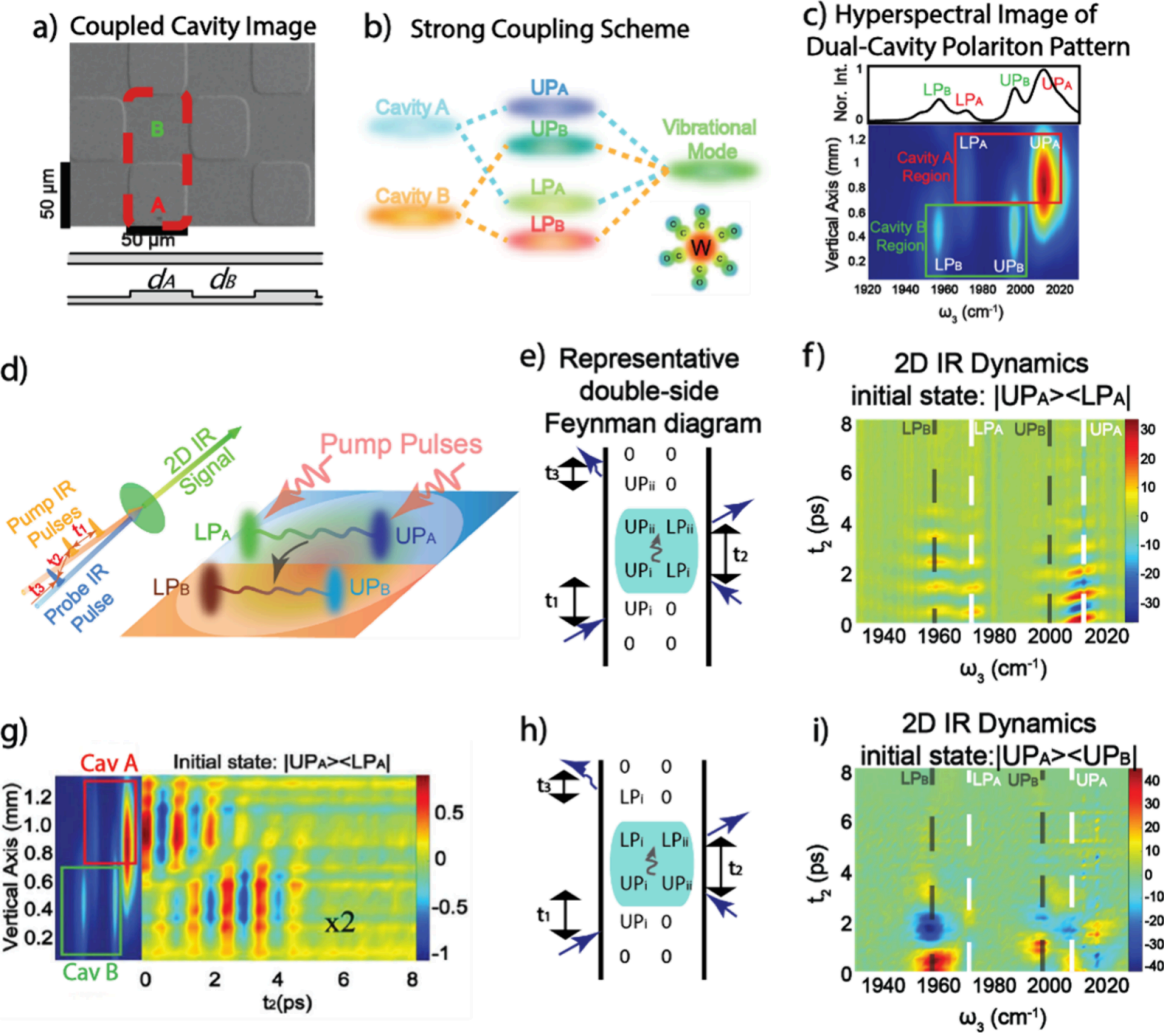
Polariton coherence in dual-cavity coupled molecular systems. (a) SEM image of dual-cavity mirror (top) with illustration of its intersection (bottom). (b) Energy diagram of molecular vibrations coupled to dual-cavity modes. (c) Hyperspectral image of dual-cavity polariton transmission. Depiction of (d) intracavity polariton coherence propagation with 2D IR pulse sequence and (e) corresponding Feynman diagram. (f) 2D IR dynamics at ω_1_ = ω_UPA_ with initial states of |UP_A_><LP_A_|, showing coherence transfer to |UP_B_><LP_B_|. (g) This coherence transfer is visualized spatially. The dual cavity can also facilitate intercavity coherence, as depicted in (h) the Feynman diagrams. (i) The existence of intercavity coherence |UP_A_><UP_B_| is evidenced by the beating pattern between the UP_A_ and UP_B_ states. (a–f), (h–i) Reprinted with permission from ref (69). Copyright 2022 Wiley. (g) Reprinted with permission from ref (71). Copyright 2022 Wiley.

While the polariton bleach can serve as a basis for ultrafast optical switches, the creation of pure polariton coherence within the optical cavity^4,68^ opens up intriguing possibilities for using the molecular vibrational polaritons as a platform to investigate or emulate quantum phenomena. To test the tunability of such polaritonic quantum states, a checkerboard dual cavity with ZnO/Ge paired DBRs was designed and deployed to strongly couple to the W(CO)_6_ molecules^69^ (Figure 32a). Within each cavity, the vibrational mode of W(CO)_6_ was strongly coupled to the respective cavity modes in which they were situated, giving rise to two distinct sets of polaritons characterized by different energies (Figure 32b) and spatial distributions (Figure 32c). The initial spectroscopic study indicated that at long t_2_, the excited polaritons in cavity A could influence optical transitions of those in cavity B via incoherent interactions of the dark modes, resulting in Rabi splitting contraction. A modified TMM model showcased that achieving delocalized nonlinearity in polaritons required both photon delocalization and molecular nonlinearity.^61^ As a result, the transmission of the dual-cavity system becomes
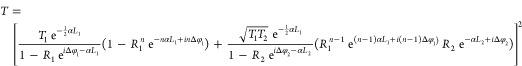
13where *T*, *R*_*i*_, *L*_*i*_ and Δφ_i_ are the transmission, reflection, cavity longitudinal length and phase shift of corresponding cavity area (A: *i* = 1; B: *i* = 2), α is the absorptive coefficient of molecules and *N*_*R*_ represents the number of round trips (in A area) before photon hopping to the adjacent cavity. This modified model better described the transmission of two sets of polaritons.

Subsequently, employing a tailored pump 2D IR scheme, Xiang and co-workers^69^ achieved precise control over various types of polariton coherences. These included intracavity coherences confined to the same cavity as well as intracavity coherences between polaritons residing in different cavities. When intracavity polariton coherence was formed in cavity A (e.g., |UP_A_><LP_A_|, Figure 32d), a similar coherence pattern emerged in cavity B (e.g., |UP_B_><LP_B_|, Figure 32e–f). Using a home-built 2D IR imaging setup, the authors clearly showed the initial coherence in cavity A transferred to cavity B over space (Figure 32g). An intriguing observation during both the coherence transfer at early t_2_ and population transfer at later t_2_ was the unidirectional nature of these processes. Specifically, only coherences (or populations) from cavity A could transfer to B, not vice versa. This non-Hermitian behavior stemmed from the energy offset between the dispersion curve of cavities A and B. Because the experiments were conducted at low *k*_||_, cavity A consistently had higher energy than B at the same *k*_||_, making it unfavorable for photons from B to scatter to A while conserving energy and momentum. Conversely, photons scattering in the opposite direction were always feasible, facilitating both coherence and population transfers. This restriction was lifted at high *k*_||_, which was also observed in the same study.

In contrast, intercavity coherences are more susceptible to spatial environmental fluctuations. Coherences at low frequencies (e.g., 10 cm^–1^) formed between polaritons that were energetically close (|UP_A_><UP_B_| or |LP_A_><LP_B_|) could be successfully built (Figure 32h–i). However, the inability to create robust |UP_A_><LP_B_| or |LP_A_><UP_B_| at higher frequencies, even at 30 cm^–1^, highlighted the challenge of simultaneously overcoming spatial and energetic fluctuations within this artificial system.

### 6.3 Robust Polaritonic Multiqubit Systems

**Figure 33:**
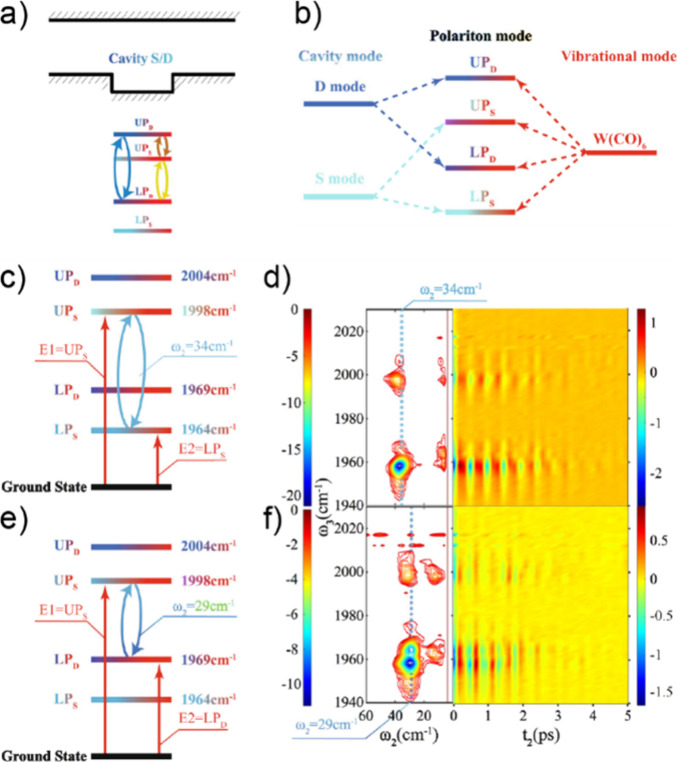
Multiqubit polaritonic system enabled by quantum confinements. (a) Schematic illustration of laterally confined cavity and the polariton coherences. (b) Energy diagram showing the strong coupling between photonic mode S/D and W(CO)_6_ vibrational mode. (c) The coherence that originated from the same modes of the confined cavity shows a strong signal in (d). (e) The coherence between UP_S_ and LP_D_ is composed of different optical cavity modes. Because they still reside in the same physical locations, their coherences also show strong oscillating signals in (f). Reprinted with permission from ref (70). Copyright 2023 National Academy of Sciences.

In an effort to mitigate vibrational decoherence caused by environmental fluctuations in VSC systems, Yang and co-workers designed a laterally confined F–P cavity with 25-μm confinement in one lateral dimension (1-μm depth, Figure 33a).^70^ This design, inspired by the “particle in a box” concept, used the lateral confinements to disrupt the continuous dispersion curve of the photonic modes, yielding discrete quantized dispersions. Consequently, at certain values of *k*_||_, two cavity modes (S and D modes) with closely matched energies coexist. These two cavity modes individually strongly coupled to the C=O vibrational mode of W(CO)_6_, forming two pairs of polaritons (Figure 33b). Notably, these two pairs of polaritons shared the same spatial domain, in contrast to the dual-cavity system.^69^ Because molecular vibrational components underwent identical spatial fluctuations, even when strongly coupled to different cavity modes, polariton coherences were observed not only between states associated with the same cavity mode (|UP_S_><LP_S_|, Figure 33c–d) but also between those of orthogonal cavity modes (|UP_S_><LP_D_|, Figure 33e–f).

From the analytical solution of the Lindblad equations, it became evident that the decoherence term of polariton coherence |UP_S_><LP_D_| accounts for both the energetic (γ_1_^2^(*E*_UP_S__ – *E*_LP_D__)^2^) and spatial (γ_2_^2^(*x*_S_ – *x*_D_)^2^) fluctuations, but the latter remained negligible because of the spatial overlap between S and D modes. This was a departure from the intercavity coherence in the dual-cavity system,^69^ where spatial fluctuations in decoherence were large due to the distributed nature of cavity modes. In the lateral confined cavity, the substantial spatial overlap of S and D cavity modes gives rise to a larger tunability of the energetics, resulting in resilient coherence between the polaritons with 29 cm^–1^ splitting. By controlling the cavity design, a broader range of energetics tuning is achievable. In combination with confined and dual-cavity modalities, these advancements pave the way for more complicated platforms aimed at simulating coherence dynamics.

### 6.4 Challenges and Opportunities

These pioneering investigations and innovative cavity configurations have illuminated the potential of using molecular polaritons as platforms for quantum information technology.^4,69^ One promising avenue is the integration of diverse cavity structures to create intricate patterns, akin to the Hamiltonian found in light harvesting complexes,^231^ thereby facilitating the spatial visualization of coherence and energy transfers. Of course, the challenge to fulfill this potential is also significant, as listed below.

(i) Given that molecular systems are mostly homogeneous without an external field, the heterostructures on cavity mirrors become pivotal in crafting Hamiltonians for simulating quantum phenomena or multifunctional information processing toolboxes. However, the spatial resolution of IR-regime vibrational coherence is constrained to a few microns due to the IR diffraction limit. One potential solution entails pushing VSC to the single-molecule regime^138^ and distributing these units at a distance of a few microns. Nevertheless, this design requires researchers to not only amplify coherent responses beyond current instrumental sensitivity but also to establish coherent connections between neighboring units.

(ii) Prolonging the polariton decoherence lifetime^71^ will preserve more resilient polariton coherences in the time domain, allowing quantum information to propagate further within the system. Luckily, the vibrational oscillation period is considerably shorter than that of its spin counterparts. Thus, similar figure of merits could be achieved with a much shorter coherence lifetime. Since the dephasing lifetime of polariton modes inherently hinges on both the line width of molecular vibrational modes and the optical cavity modes, optical cavities characterized by higher Q-factors will favor the robustness of polariton coherence in the time domain. However, the line widths of molecular vibrational modes must also be controlled by choice of solvents,^113^ temperature,^232,233^ purity and more. It may become imperative to eliminate solvent interactions by grafting vibrational chromophores to molecular frameworks, subsequently lowering the temperature to remove decoherence channels due to low frequency vibrational modes.

(iii) Lastly, a theoretical underpinning on the origins of the polariton bleach signal, especially its connection to the famous photon blockage effects,^8,9^ could be useful to systematically design and harness this unique phenomenon for photonic signal transduction and quantum simulation developments.

## 7 Polarization Properties under Strong-Coupling Regime

Polarizations, an inherent characteristic of molecules, offer a unique avenue for governing molecules through polarization specific interactions and for manipulating the polarization of photons emitted by these molecules. The introduction of strong coupling with cavity modes has provided a new dimension to augment the efficacy of polarization-specific light–matter interactions. Specifically, a promising frontier is emerging in chirality control through polaritons.

In hybridized molecular polariton systems, the resultant polaritonic states integrate the polarization attributes of both molecular and cavity modes. As elaborated in section 2, the polariton Hamiltonian matrix can be diagonalized to yield multiple eigenenergies accompanied by corresponding eigenvectors. The former describes the energy landscape and momentum dispersion, while the latter represents the polarization characteristics to the emergent polaritonic states. In principle, tuning the phase information pertaining to molecular orientations or cavity structures empowers the precise engineering of polariton polarization inherited from both molecular and photonic constituents.^93,234^ Building upon such strategies, the polarized molecular responses under strong coupling can be achieved by harnessing diverse micro- or nanostructured cavities and preparing the molecular systems with specific orientations.^133,134,235−248^ Taking the polarization into consideration, the strong-coupling phenomenon within molecular systems finds utility in structural detection with ultrahigh sensitivity and the effective modulation of molecular orientation in various ultrafast processes under strong-coupling condition.

### 7.1 Theoretical Development of Polarized Polaritonic Effect

Within the framework of a solution-based strong-coupling system composed of linear molecules, the predominant polarizations of polaritons stem from the cavity photonic mode, owing to the inherent randomized molecular orientations. To effectively incorporate the orientational insights originating from the molecular part into polaritons, it becomes imperative to align the molecular dipoles through solid-state^243,245^ or liquid crystal molecules.^242^ Conversely, it is noteworthy that the polarization properties of molecular modes which strongly coupled with the cavity mode can be preserved and modify the polarizations of cavity photons.^133,240,249^

**Figure 34:**
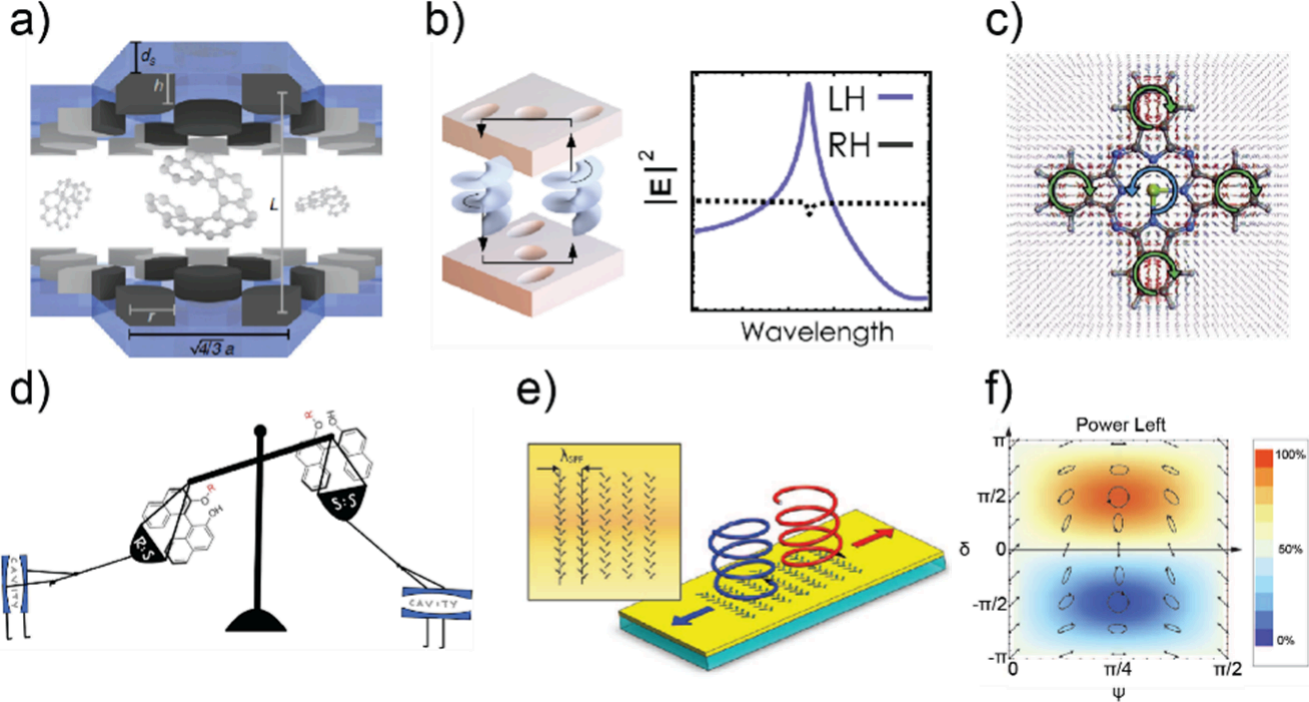
Schemes of theoretical designs of highly polarized polaritonic systems. (a) Chiral polaritons created by strong coupling between the cavity with chiral structure and molecules with chirality. Reprinted with permission from ref (247). Copyright 2020 American Institute of Physics. (b) Circular dichroism induced by the strongly coupled DNA molecules. Reprinted with permission from ref (236). Copyright 2022 American Chemical Society. (c) Coherent ring-current migration of Mg-phthalocyanine in the X-ray cavity. Reprinted with permission from ref (250). Copyright 2022 Royal Society of Chemistry. (d) Enhanced diastereoselectivity in enantioenrichment of (S)-Boc-Proline functionalized BINOL via different circularly polarized cavity modes. Reprinted with permission from ref (235). Copyright 2022 American Chemical Society. (e) Multicolumn pairs of plasmonic couplers induced SPPs. (f) Incident polarization enables tuning of SPP directionality. (e–f) Reprinted with permission from ref (248). Copyright 2013 American Association for the Advancement of Science.

Scott et al.^247^ introduced a theoretical design of various chiral cavities incorporating microresonators, photonic crystals, and Si-based plasmonic structures (schematic shown in Figure 34a). Typically, a chiral cavity mode can be viewed as a combination of two orthogonal linear polarized modes and typically requires TE-TM degeneracy (two degenerate cavity modes with orthogonal linear polarizations). The precise control over materials and cavity geometric parameters can fulfill such a requirement in creating the chiral cavity modes. Such designs can significantly enhance the sensitivity and selectivity of chiral molecular responses via light–matter strong coupling, particularly vibrational circular dichroism. Similar theoretical blueprints of chiral polaritons have been reported in more specific molecular systems. Baranov and colleagues,^236^ for instance, proposed a single-handed chiral cavity design selectively allowing light of a specific chirality to propagate within the cavity. Hence, biomolecules, such as DNA or drug compounds sharing the same chiral structure, can strongly couple to the same cavity, leading to strong emissions of polaritonic signals (Figure 34b). This renders the cavity an exceptionally sensitive chiral molecular sensor.

Concurrently, Mukamel et al.^133,134^ theoretically explored the utility of chiral optical cavity designs to magnesium-porphyrin compounds, known for their coherent ring currents that exhibit variable X-ray circular dichroism signals due to their aromatic structure^250,251^ (Figure 34c). The ring current of the Mg-porphyrin was intricately linked to the molecular electronic structure, characterized by a distinctive time scale of 150 fs.^250^ Consequently, the strong coupling between Mg-porphyrin electronic transition and chiral optical cavity mode holds the potential to significantly amplify the X-ray circular dichroism effect.^133,134^

Vu and colleagues^235^ harnessed an optical cavity to enhance the diastereocontrol in the context of the excited-state proton transfer (ESPT)-driven diastereomeric enrichment between a chiral diene and a chiral dienophile, based on their quantum electrodynamics (QED) generalization of time-dependent density functional theory. By exploring the distinct interactions of circularly polarized cavity modes with the different diastereomers of the reacting molecules, this approach enables selective coupling of reactant molecules with the appropriate handedness to the chiral cavity mode, thereby shifting the balance of the enrichment toward the desirable direction (Figure 34d). This theoretical innovation presents the prospect of a chirality-based optical switch for precise control over chemical or biochemical reactivity through the chiral specific molecular strong coupling.

Given the predominant focus on circular polarization in the above-mentioned reports on molecular polaritons, it has become imperative to establish a comprehensive understanding of the chirality of cavity modes. So far, two approaches have been proposed: one involves leveraging helically polarized cavity modes, while the other hinges upon the design and fabrication of micro- or nanometal-dielectric structures, such as polarization-sensitive apertures crafted through dual nanogratings^248^ (Figure 34e). These apertures serve to selectively scatter incident light with specific linear polarizations. When multiple sets of the nanograting columns are etched onto a metal surface, interferences between linearly polarized light fields occur. Precise adjustments to the geometric parameters of a multicolumn nanostructure grant control over both the phase and intensity of the light fields. As a result, intricate polarization patterns of the SPP can be engineered in this innovative way (Figure 34f).

### 7.2 Experimental Polarization Effects in Molecular Polaritons

**Figure 35:**
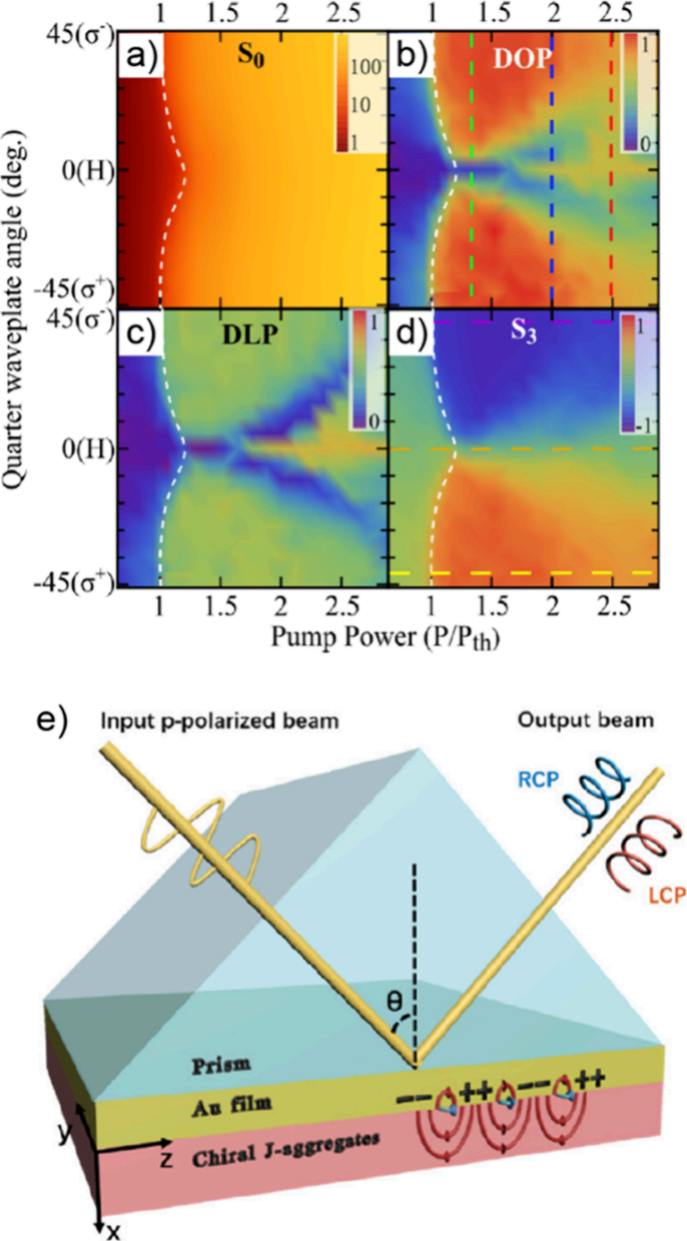
Experimental realization of polarization effect in exciton polaritons. (a) Total emission intensity S_0_ in arbitrary units. (b) Degrees of polarization (DOP). (c) Degrees of linear polarization (DLP). (d) S_3_ as a function of pump power P and ellipticity (QWP angle). QWP angles ∓45° and 0° correspond to right-, left-circular, and linear polarized excitation. White dashed lines mark the condensation threshold. (a–d) Reproduced with permission from ref (249). Copyright 2020 American Physical Society. (e) Circular dichroism in surface plasmon polaritons with J-aggregates. Reproduced with permission from ref (244). Copyright 2021 American Chemical Society.

A couple of endeavors have been deployed to experimentally manipulate exciton polariton polarization. In one such exciton polariton system, the exciton mode of InGaAs quantum wells strongly coupled to the GaAs microcavity mode^249^ (Figure 35a–d). Upon excitation, the linear (DLP, Figure 35c) and circular polarizations (Stokes parameter, S_3_, Figure 35d) of polariton condensates can be tuned in terms of excitation power density and polarization that is controlled by a quarter wave-plate (QWP). The authors attributed the evolution of the condensate polarization (pseudospin) properties to the interactions between polariton condensates and uncondensed exciton reservoirs with varying polarizations of optical excitation. The imbalance of polariton condensate pseudospin would induce synthetic effective in-plane and out-of-plane magnetic fields, which rendered pseudospin precession to convert from linear to circular polarization or vice versa.^238,239,249,252^ Another type of polarization manipulation was achieved in Guo et al.’s work.^244^ The exciton mode of chiral J-aggregates (TDBC) was strongly coupled to the surface plasmonic cavity mode of the Au film on the surface of a prism, thereby controlling the circular dichroism (CD) signal (Figure 35e). Notably, the CD signal strength could be tuned, from weakest to strongest, contingent upon the linear polarization of the incident light, from parallel to perpendicular relative to the orientation of the chiral emitters at the interface between metal and J-aggregates.

**Figure 36:**
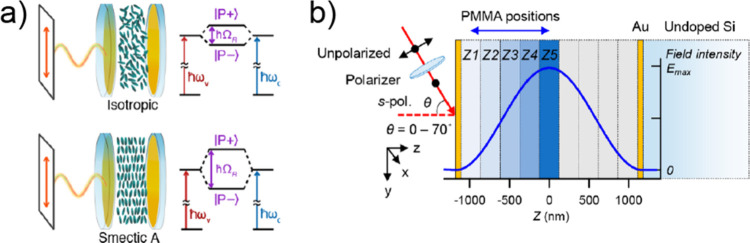
Experimental realization of polarization effect in molecular polaritons. (a) Coupling strength tunability of strongly coupled liquid crystal molecules via e-field induced anisotropy modification. Reproduced with permission from ref (242). Copyright 2022 American Chemical Society. (b) Coupling strength modulation by incident polarization and spatial distribution of molecules. Reproduced with permission from ref (245). Copyright 2018 American Chemical Society.

In contrast, the experimental exploration and manipulation of the molecular vibrational strong-coupling regime are in their infancy, with limited outcomes like the liquid crystal strong-coupling system.^117,242^ Yanagi et al.^242^ investigated the vibrational strong coupling between C≡N stretching mode of 4-cyano-4′-octylbiphenyl liquid crystal molecules and optical cavity mode in a gold-coated F–P cavity (Figure 36a). They revealed that when liquid crystal molecules transitioned from isotropic to smectic phases under an applied electric field, their orientations became near parallel to the polarization of incident light, resulting in a 30% increase in the coupling strength. To elucidate the relationship between coupling strength and the relative molecular orientation to the polarization of the external field, they introduced the concept of an effective orientation factor. This finding demostrated the effective number of vibrational oscillators that can be tuned under a fixed external optical field.

Another significant contribution by Simpkins et al.^245^ involved the precise control of the spatial distribution of the PMMA film to investigate the polarization effect in vibrational strong coupling (Figure 36b). Glass (methylsiloxane polymer, [H_3_CSiO_2_]_*m*_[SiO_2_]_*n*−*m*_, SOG) spacing layers were used to locate the PMMA layer to various positions in between the two gold cavity mirrors. In this way, they considered that the intensity of the cavity mode was spatially modulated and depended on polarizations. This design led to the conclusion that the coupling strength could be tuned by altering the relative position and orientation of the molecular vibrational oscillators and cavity field. These works serve as pioneering efforts in introducing polarization as a new factor for enhancing control over the molecular polariton systems.

### 7.3 Challenges and Opportunities

Significantly, most experimental realizations of polarization control are still operated within the linear polarized regime, even though theoretical predictions have hinted at compelling prospects within the realm of circular polarized light–matter strong coupling. The primary challenge stems from design and fabrication of circular polarized cavities, especially F–P cavities, which have traditionally been the vessels for strong-coupling experiments. However, given the diverse array of designs for circular polarized cavities and the advancements in photonic fabrication techniques, this field is expected to rapidly advance in the coming years.

Polariton polarization constitutes a pivotal degree of freedom in both molecular and photonic science. To facilitate precise manipulation of polariton responses, previous research has necessitated either the adjustment of cavity micro- or nanostructures^236,247,248^ or the alignment of molecules.^242,244,245^ These endeavors are poised to pave future exploration, encompassing polarization-dependent intramolecular energy distribution, single-molecule polaritons and strong coupling in the molecular surfaces and interfaces—domains where the molecular orientation matters. In this context, polariton polarization offers possibilities in quantum simulation leveraging molecular structural properties, such as chirality or birefringence, when hybridized with cavity photons, engendering polarization-induced optical switch, and controlling ultrafast molecular processes.

## 8 Conclusion and Outlook

The realm of strong light–matter coupling opens up a new dimension in which photons are harnessed to manipulate matter and, reciprocally, matter is employed to tailor photon characteristics—an arena giving rise to the intriguing domain of polariton chemistry. This is a realm of novel chemical control that capitalizes on swift energy exchange between a material and photonic constituents. It exhibits a certain resemblance to the well-established concept of plasmon-enabled chemistry, although the enhancements observed in plasmonics may stem from either electronic or photonic sources. In contrast, polaritons represent a purely photonic force capable of shaping material properties. Thus, it introduces a broad and innovative paradigm—photonic chemistry—that leverages novel photonic attributes to modulate molecular properties. Furthermore, the emergence of molecular polaritons stands to propel quantum technologies toward platforms founded on intricate molecular systems, augmenting the intricacy of quantum simulations. These developments can harness the progress made in photonics, encompassing nanofabrication techniques concurrently developed in the fields of communication and materials science, thereby charting a promising trajectory toward exerting unprecedented control over molecules and photons in temporal, spatial, and energy scales.
